# Long-term Effectiveness of mHealth Physical Activity Interventions: Systematic Review and Meta-analysis of Randomized Controlled Trials

**DOI:** 10.2196/26699

**Published:** 2021-04-30

**Authors:** Annette Mönninghoff, Jan Niklas Kramer, Alexander Jan Hess, Kamila Ismailova, Gisbert W Teepe, Lorainne Tudor Car, Falk Müller-Riemenschneider, Tobias Kowatsch

**Affiliations:** 1 Institute for Customer Insight University of St. Gallen St. Gallen Switzerland; 2 Institute for Mobility University of St. Gallen St. Gallen Switzerland; 3 Centre for Digital Health Interventions Institute of Technology Management University of St. Gallen St. Gallen Switzerland; 4 CSS Insurance Lucerne Switzerland; 5 Translational Neuromodeling Unit Institute for Biomedical Engineering University of Zurich and ETH Zurich Zurich Switzerland; 6 Centre for Digital Health Interventions Department of Management, Technology, and Economics ETH Zurich Zurich Switzerland; 7 Lee Kong Chian School of Medicine Nanyang Technological University Singapore Singapore; 8 School of Public Health Department of Primary Care and Public Health Imperial College London London United Kingdom; 9 Saw Swee Hock School of Public Health National University of Singapore Singapore Singapore; 10 Future Health Technologies Programme, Campus for Research Excellence and Technological Enterprise (CREATE) Singapore-ETH Centre Singapore Singapore

**Keywords:** mHealth, physical activity, systematic review, meta-analysis, mobile phone

## Abstract

**Background:**

Mobile health (mHealth) interventions can increase physical activity (PA); however, their long-term impact is not well understood.

**Objective:**

The primary aim of this study is to understand the immediate and long-term effects of mHealth interventions on PA. The secondary aim is to explore potential effect moderators.

**Methods:**

We performed this study according to the Cochrane and PRISMA (Preferred Reporting Items for Systematic Reviews and Meta-Analyses) guidelines. We searched PubMed, the Cochrane Library, SCOPUS, and PsycINFO in July 2020. Eligible studies included randomized controlled trials of mHealth interventions targeting PA as a primary outcome in adults. Eligible outcome measures were walking, moderate-to-vigorous physical activity (MVPA), total physical activity (TPA), and energy expenditure. Where reported, we extracted data for 3 time points (ie, end of intervention, follow-up ≤6 months, and follow-up >6 months). To explore effect moderators, we performed subgroup analyses by population, intervention design, and control group type. Results were summarized using random effects meta-analysis. Risk of bias was assessed using the Cochrane Collaboration tool.

**Results:**

Of the 2828 identified studies, 117 were included. These studies reported on 21,118 participants with a mean age of 52.03 (SD 14.14) years, of whom 58.99% (n=12,459) were female. mHealth interventions significantly increased PA across all the 4 outcome measures at the end of intervention (walking standardized mean difference [SMD] 0.46, 95% CI 0.36-0.55; *P*<.001; MVPA SMD 0.28, 95% CI 0.21-0.35; *P*<.001; TPA SMD 0.34, 95% CI 0.20-0.47; *P*<.001; energy expenditure SMD 0.44, 95% CI 0.13-0.75; *P*=.01). Only 33 studies reported short-term follow-up measurements, and 8 studies reported long-term follow-up measurements in addition to end-of-intervention results. In the short term, effects were sustained for walking (SMD 0.26, 95% CI 0.09-0.42; *P*=.002), MVPA (SMD 0.20, 95% CI 0.05-0.35; *P*=.008), and TPA (SMD 0.53, 95% CI 0.13-0.93; *P*=.009). In the long term, effects were also sustained for walking (SMD 0.25, 95% CI 0.10-0.39; *P*=.001) and MVPA (SMD 0.19, 95% CI 0.11-0.27; *P*<.001). We found the study population to be an effect moderator, with higher effect scores in sick and at-risk populations. PA was increased both in scalable and nonscalable mHealth intervention designs and regardless of the control group type. The risk of bias was rated high in 80.3% (94/117) of the studies. Heterogeneity was significant, resulting in low to very low quality of evidence.

**Conclusions:**

mHealth interventions can foster small to moderate increases in PA. The effects are maintained long term; however, the effect size decreases over time. The results encourage using mHealth interventions in at-risk and sick populations and support the use of scalable mHealth intervention designs to affordably reach large populations. However, given the low evidence quality, further methodologically rigorous studies are warranted to evaluate the long-term effects.

## Introduction

### Background

In recent decades, populations have become increasingly sedentary. The World Health Organization (WHO) recommends 150 minutes of moderate-intensity physical activity (PA) or 75 minutes of vigorous-intensity PA per week for adults and 60 minutes of moderate-to-vigorous physical activity (MVPA) for adolescents per day [1]. An estimated 28% of adults worldwide do not meet these guidelines [2]. The prevalence of inactivity is high in Latin America and many high-income countries, with approximately every second adult inactive in Brazil or Saudi Arabia, and 40% of adults insufficiently active in the United States [2].

According to the WHO, physical inactivity is 1 of the 4 core modifiable risk factors for noncommunicable diseases (NCDs). As such, it is as important to be addressed as tobacco use or obesity and proven to increase the risk of cancer, cardiovascular diseases, diabetes, dementia, and depression [3-6].

In response to the high prevalence and substantial risk posed by physical inactivity, the WHO has formulated a target to reduce physical inactivity by 10% by 2025 as part of its strategy against NCDs [7]. Scaling up PA interventions is key to achieving the WHO target. However, there are various barriers, including cost, resource restrictions, and poorly scalable intervention designs [8,9]. Owing to the increasing dissemination and ubiquity of mobile technology, mobile technology–based interventions, that is, mobile health (mHealth), have been discussed as a solution for overcoming scalability challenges [10,11]. There are only a few examples of nationwide mHealth programs such as the *NHS Diabetes Prevention Program* [12] in the United Kingdom, the 10,000 steps program in Australia [13], and the *National Steps Challenge* and *Live Healthy SG* in Singapore [14,15]. Most governments and health organizations are still hesitant about rolling out mHealth PA programs, as clear evidence for the effectiveness of mHealth interventions for sustainable behavior change is lacking [16,17].

Previous evidence for the effectiveness of mHealth interventions on PA is mixed (Multimedia Appendix 1 [18-31]). Most existing meta-analyses found significant positive effects on PA in sick and at-risk populations, with effect sizes ranging from small to large [18-28]. However, some studies did not find significant effects or reported conflicting results [29-31]. There is limited evidence for the sustainability of increased PA levels beyond the end of intervention. Only 2 studies quantitatively analyzed long-term effects: one review found that PA increases are maintained up to 3-4 years after the intervention [20] and the other did not find significant long-term results [31]. Kirk et al [25] and Romeo et al [30] found that shorter mHealth PA interventions (<16 weeks and <12 weeks, respectively) are more effective than longer ones, indicating that effects might not be maintained in the long term.

We also lack clarity on how population types, intervention design, and control group type moderate the impact of mHealth interventions on PA. Only 3 studies performed subgroup analyses according to population type with mixed results. A total of 2 studies found interventions to be equally effective in sick and healthy populations [23,30], and 1 review found mHealth interventions to be more effective in sick populations; however, the results were not statistically significant [27]. Most studies focused exclusively on sick or at-risk populations [21,22,24-26,28,31], making it difficult to draw clear conclusions.

The design of mHealth interventions influences the degree to which they are scalable. The promise of mHealth is that the technology itself (ie, without costly and limited human resources) promotes active lifestyles. However, these highly scalable interventions miss the element of human-to-human interaction, which is a potentially important active ingredient in behavior change interventions. Current evidence draws an inconclusive picture: existing studies have found no effects on PA when interventions are scalable [24,30] (ie, mHealth interventions without human-to-human interactions), stronger effects when interventions are nonscalable (ie, mHealth interventions with human-to-human interactions) [27,32], stronger effects in scalable interventions [20], or no moderating effects [22]. Thus, we need a comprehensive evaluation of scalable versus nonscalable designs to judge the potential of mHealth technologies in reaching large populations at low costs.

Furthermore, our current understanding of the effects of mHealth PA interventions is limited by the inclusion of different control groups in previous studies. Most previous studies included both minimal or no intervention control groups and control groups receiving an alternative intervention [18-20,22-29,31]. This makes it impossible to distinguish between the absolute effect of mHealth PA interventions on behavior and the degree to which mHealth interventions are superior to alternative nonmobile designs or the standard of care.

### Objectives

Accordingly, we sought to comprehensively collate and analyze trials evaluating mHealth interventions that promote PA in adult populations. Our primary aim is to understand the long-term impact of these interventions on PA. Our secondary aim is to explore potential effect moderators (population type, intervention design, and control group type), to understand which populations can benefit from mHealth interventions, to understand if scalable mHealth intervention designs are effective, and to understand if mHealth interventions produce superior results to nonmobile interventions.

## Methods

### Overview

This study was performed according to the Cochrane methodology, and the results were reported following the PRISMA (Preferred Reporting Items for Systematic Reviews and Meta-Analyses) guidelines. We searched PubMed, the Cochrane Library, SCOPUS, and PsycINFO for randomized controlled trials (RCTs) on mHealth interventions targeting PA increases (all search strategies are given in Multimedia Appendices 2 and 3) published from database inception to July 3, 2020. We also searched the reference lists of the relevant existing systematic reviews for eligible studies. This study was registered with PROSPERO (CRD 42019124716).

### Eligibility Criteria

Studies were eligible if they assessed the impact of mHealth interventions on PA as a primary study outcome in individuals aged 18 years or more and were published in English. Eligible study designs were RCTs or cluster RCTs. Eligible comparators comprised no or minimal interventions and alternative interventions that did not include mobile technologies.

#### Types of Interventions

mHealth interventions were defined as programs that fully or partly deliver interventions using mobile technology such as pedometers or accelerometers with displays, activity trackers, smartphones, or tablets. We excluded interventions where the use of a mobile device was unclear (eg, telephone or website interventions) or where increasing PA was not the primary outcome. This was to ensure that interventions genuinely aimed to increase PA and to avoid including studies measuring PA only as a supplemental outcome.

#### Types of Outcomes

Eligible outcome measures were walking, MVPA, total physical activity (TPA), and energy expenditure (EE), as these outcomes are most commonly reported. Multiple outcome units were eligible per outcome measure (eg, walking in minutes and walking in steps). Studies reporting objectively measured or self-reported outcome data were eligible.

### Data Collection Process

Abstracts of all identified papers were exported and uploaded into Covidence Systematic Review software (Veritas Health Innovation Ltd, version accessed July 2020) for screening. Two reviewers independently screened the abstracts for eligibility (AM, JNK, or KI). If reviewers doubted whether an article was potentially relevant, it was included for full inspection. Next, full texts of potentially eligible papers were uploaded into Covidence and screened by 2 independent reviewers (AM, JNK, or KI). Conflicts were resolved by discussion or where required by a third reviewer. We contacted authors of potentially relevant articles for further information when needed. All reviewers were trained during a full-day workshop on eligibility criteria and software before screening.

### Data Extraction and Management

Data for each study were extracted independently by 2 reviewers (AM, JNK, KI, AJH, or GWT) using standardized extraction forms. Conflicts were resolved by discussion between 2 primary reviewers or with a third, independent reviewer. All reviewers were trained to use the extraction form and Cochrane risk of bias criteria during a full-day workshop. Where reported, data were extracted for all 4 eligible outcome measures (walking, MVPA, TPA, and EE) and time points (end of intervention, short-term follow-up [≤6 months after the end of intervention], and long-term follow-up [>6 months after the end of intervention]). If studies reported both objectively measured and self-reported outcome data, the former were used for meta-analysis. If studies only reported self-reported outcome data, these were extracted, and the quality of evidence was rated as high risk of detection bias. Data were extracted as means and SDs per outcome measure and time point. If SDs were not reported, they were calculated using the RevMan calculator and following the Cochrane Handbook [33]. Respective authors were contacted for any missing data.

### Assessment of Risk of Bias in Included Studies

Two reviewers (AM, JNK, KI, AJH, or GWT) independently assessed the risk of bias for each study using the Cochrane Collaboration tool [34]. Additional criteria for cluster RCTs were assessed [35] and documented within the *other bias* domains of the Cochrane Collaboration tool. Discrepancies were resolved by consensus between reviewers or where needed by a third reviewer. We classified studies as overall high risk of bias if they scored high in any bias domain other than *performance bias*, as blinding of participants and personnel is almost impossible in mHealth intervention studies [23]. Blinding of outcome assessors was rated as high risk if outcomes were self-reported.

### Statistical Methods

We summarized the intervention and sample characteristics of all the included studies. We quantitatively analyzed the data using RevMan software (Cochrane, version 5.4) and a DerSimonian and Laird random effects model for our meta-analysis [36]. We reported all 4 outcome measures using standardized mean differences (SMDs) and 95% CI. Where appropriate (eg, if one mHealth intervention was compared with a minimal and alternative nonmobile intervention), we combined means and SDs of control or intervention groups following the Cochrane Handbook [33]. We classified populations into 3 groups based on the reported recruitment criteria: sick, at-risk, and healthy. The sick group included populations experiencing illnesses such as diabetes, cancer, chronic obstructive pulmonary disease, and coronary heart disease. The at-risk group included inactive or sedentary, older, overweight, and obese populations. We classified mHealth interventions into 2 designs: scalable and nonscalable. Scalability is defined as the ability to scale up an intervention without requiring human resources. Consequently, scalable mHealth interventions were defined as interventions that only leveraged automated components without any human-to-human interactions. Nonscalable mHealth interventions included human-to-human interactions, such as coaching, in-person feedback, or group activity sessions. We classified control groups into no or minimal interventions (no intervention or information material only) and alternative (nonmobile) interventions.

Following the recommendations of Richardson et al [37] and the Cochrane Handbook [33], a subgroup analysis was performed based on end-of-intervention values for all outcomes where at least 10 studies were available. We a priori defined 3 subgroup analyses following the population, intervention, comparison, and outcome framework [33] to identify possible effect moderators. Our aim is to understand the impact of population type (sick, at-risk, and healthy), intervention design (scalable and nonscalable), and control group type (no or minimal and alternative).

We present the primary results using forest plots for each outcome and time point. Subgroup analyses were displayed in forest plots using end-of-intervention data. We quantified inconsistencies between studies using the *I*^2^ statistics (ie, the varying effect estimates towing to heterogeneity rather than chance) [33]. We classified *I*^2^>50% as having substantial heterogeneity [38]. We examined the significance of heterogeneity using chi-square tests (*P*≤.05). We assessed subgroup differences following the guidelines given by Richardson et al [37], which recommend testing for significant subgroup differences (*P*≤.10) and covariate distribution and comparing heterogeneity and effect sizes between subgroups. Funnel plot analysis was used to detect sampling bias. We used end-of-intervention effect values in our funnel plot analyses, as all studies reported this time point.

We conducted 3 sensitivity analyses to evaluate the robustness of our primary results: first, we excluded outlier studies; second, we excluded studies with high risk of bias; and third, we excluded studies not reporting long-term follow-up measurements to keep the study sample consistent across all time points. We used the grading of recommendations, assessment, development, and evaluation (GRADE) framework to assess the quality of evidence at the outcome measure level for the end-of-intervention time point and to report the standardized quality of evidence profiles, following the study by Guyatt et al [39].

## Results

### Overview

**Figure 1:**
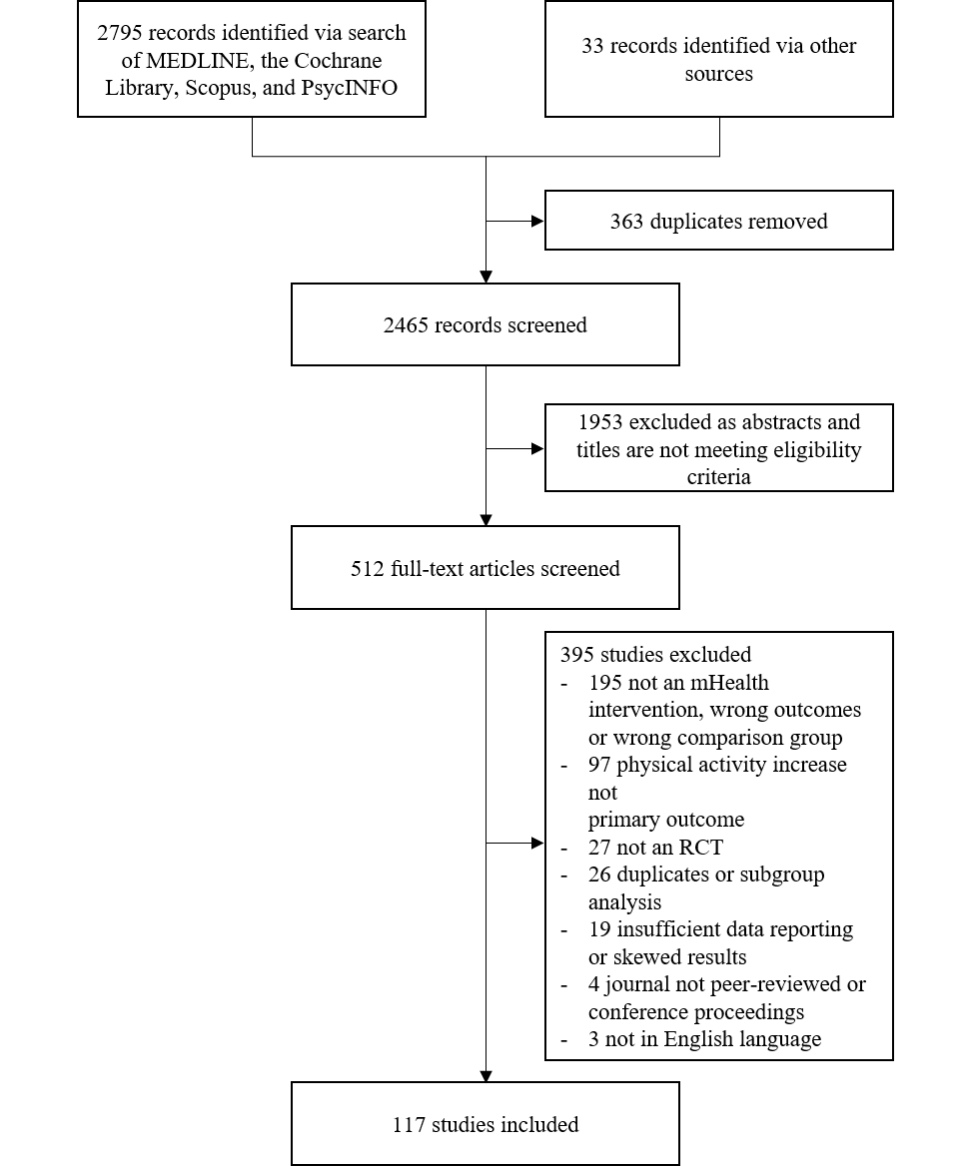
PRISMA (Preferred Reporting Items for Systematic Reviews and Meta-Analyses) chart. mHealth: mobile health; RCT: randomized controlled trial.

Of the 2828 identified studies, 512 full-text articles were screened, and 117 studies were included in the meta-analysis (Figure 1).

### Study Characteristics

Multimedia Appendix 4 [40-156] contains the included studies and their characteristics. The 117 trials represented 21,188 participants with a mean age of 52.03 years (SD 14.14), of whom 58.99% (12,459/21,118) were female. Most studies were conducted in high-income, developed regions such as North America (43/117, 36.8%), Europe (39/117, 33.3%), and Australia and New Zealand (24/117, 20.5%). Very few studies were conducted in Asia (7/117, 6.0%), Latin America (3/117, 2.6%), or Africa (1/117, 0.9%), limiting the representativeness of the evidence for low-income countries. Sample sizes ranged widely (from 15 to 1442), and the intervention duration ranged from 1 week to 2 years. All but one study [40] reported end-of-intervention results, 33 studies reported short-term follow-up results [40-72], and only 8 studies reported long-term follow-up results [61,72-78]. The mean time point for the short-term follow-up was 4.14 months (SD 2.08) after the end of intervention. The long-term follow-up measurement was taken on average after 13.96 months (SD 11.91).

Walking was the most reported outcome measure (77/117, 65.8%) [41,43,44,48,50-54,58-60,63-65,67-69,72-130], followed by MVPA (62/117, 53.0%) [42,44,46,47,49,51-55,57,59, 61-64,68,70-76,78-80,82,84-87,89-91,94,98,104,107,109,112,114, 117,125,126,131-147], TPA (33/117, 28.2%) [44,45,50,52,54, 56,64,66,72,74,76-78,84,85,89,96,110-112,114, 118,131,135,146,148-154,157], and EE (5/117, 4.3%) [61,103,137,155,156]. Most RCTs were conducted in at-risk (48/117, 41.0%) or sick populations (46/117, 39.3%). Only 19.7% (23/117) of the studies tested mHealth interventions in healthy populations within a preventative setting. In most interventions, mHealth technologies were leveraged in nonscalable intervention designs (71/117, 60.7%). Human-to-human interactions included individual coaching, group coaching, PA classes, and physical education classes. Of the 117 interventions, 45 (38.5%) used mHealth technologies without any human-to-human interactions and were thus classified as scalable. In 1 study [75], 2 mHealth interventions (scalable and nonscalable) were combined. Most mHealth interventions only leveraged basic technologies such as pedometers or accelerometers (86/117, 73.5%), text messages (20/117, 17.1%), or websites (20/117, 17.1%). Although some recent studies pioneered innovative mHealth technologies [49,96], overall, only a few studies used advanced mHealth technologies such as automated individualized feedback (19/117, 16.2%), mobile phone apps (15/117, 12.8%), social comparison (10/117, 8.5%), and automated coaching or virtual advisors (5/117, 4.3%). Most studies had no or minimal intervention control groups (83/117, 70.9%). Only a few trials had alternative intervention control groups (22/117, 18.8%). Different control group types were combined into one control group in 12 cases.

### Meta-analysis of mHealth Interventions on PA

**Figure 2:**
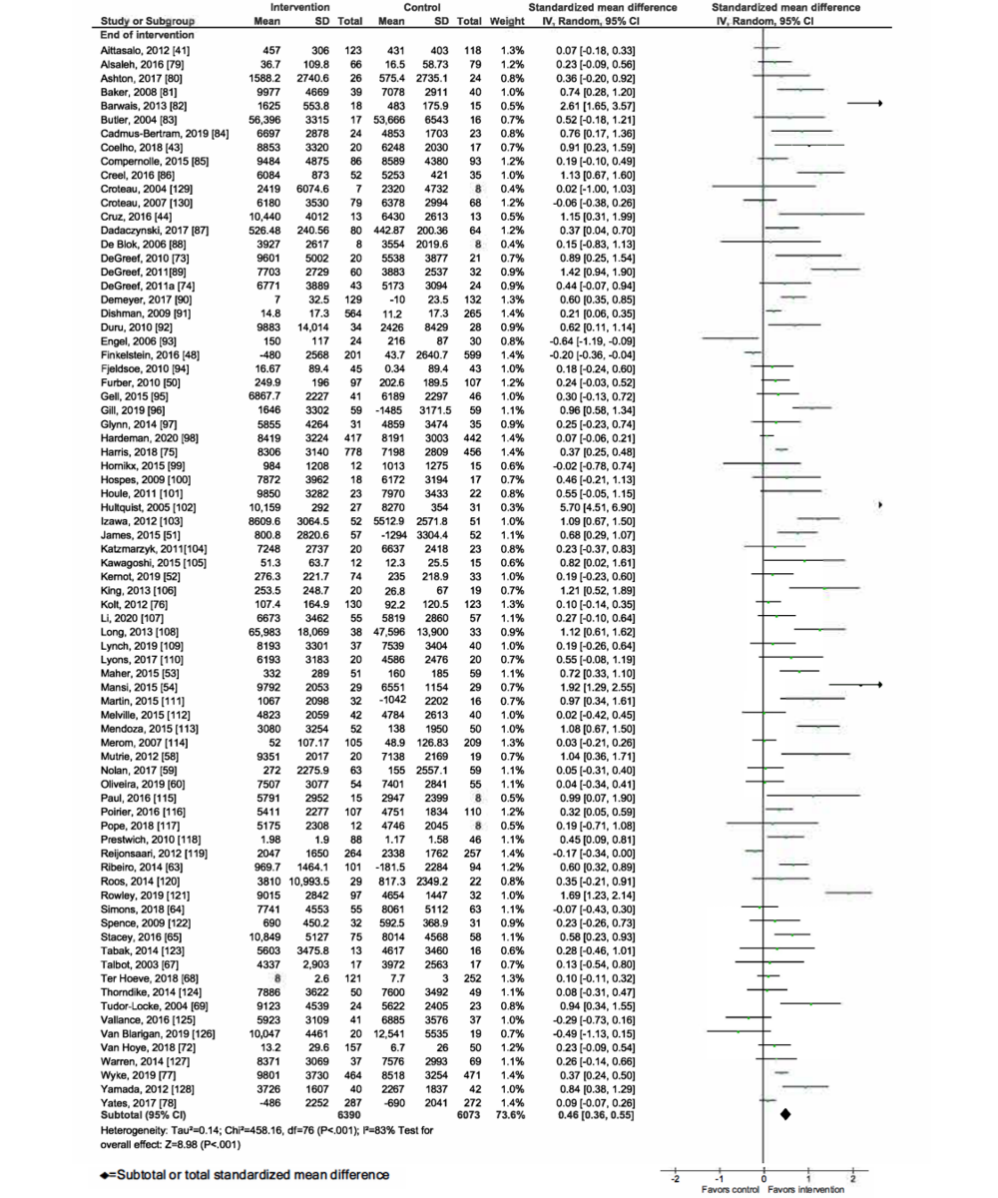
Primary outcome analysis for the outcome walking at timepoint end of intervention.

**Figure 3:**
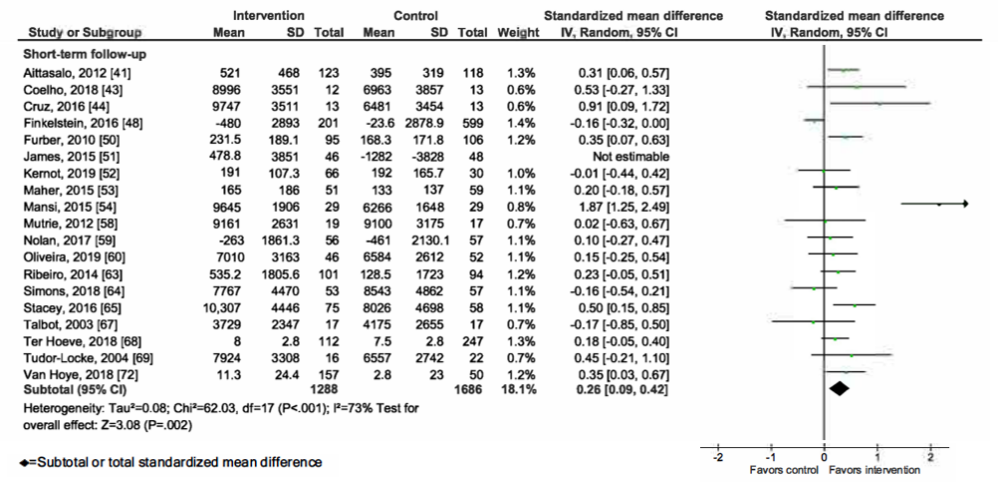
Primary outcome analysis for the outcome walking at timepoint short-term follow-up.

**Figure 4:**
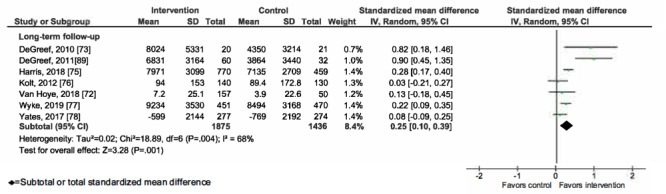
Primary outcome analysis for the outcome walking at timepoint long-term follow-up.

**Figure 5:**
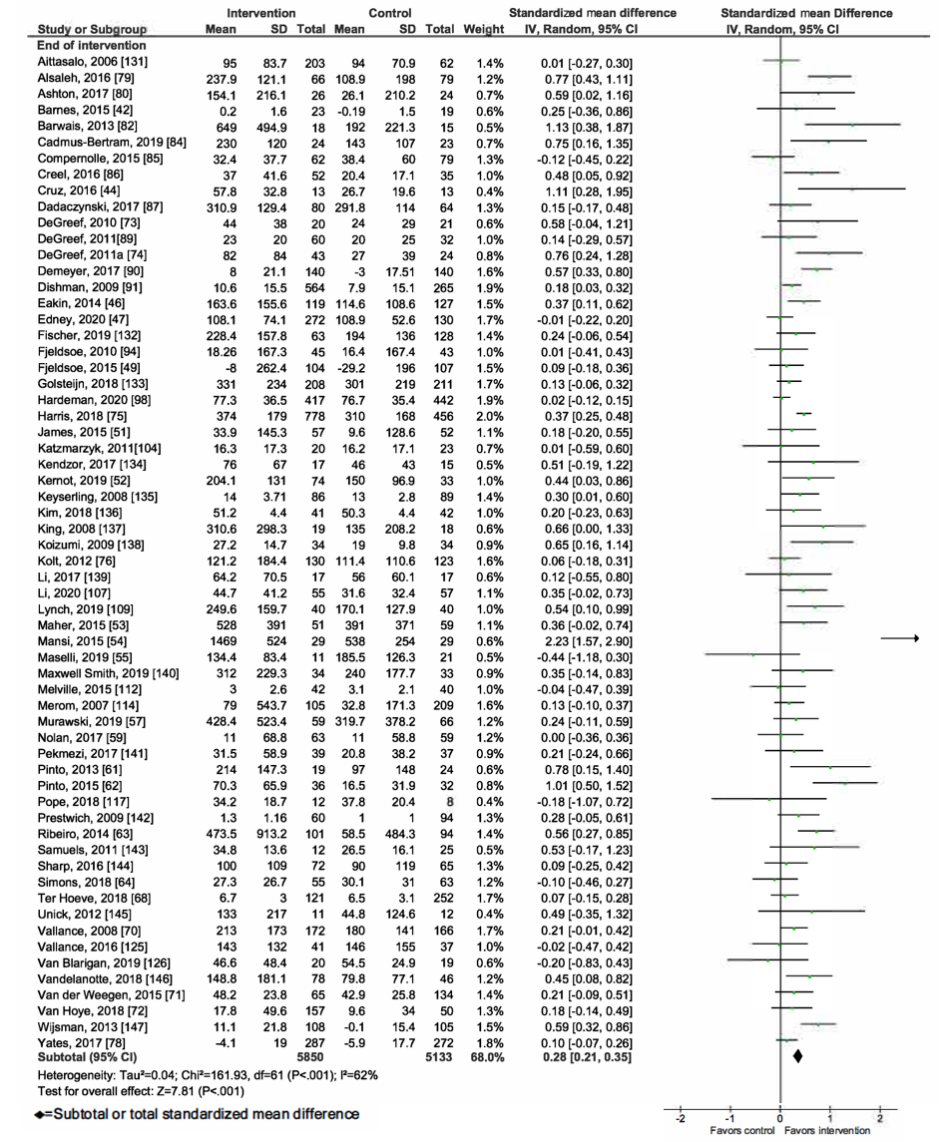
Primary outcome analysis for the outcome moderate-to-vigorous physical activity at timepoint end of intervention.

**Figure 6:**
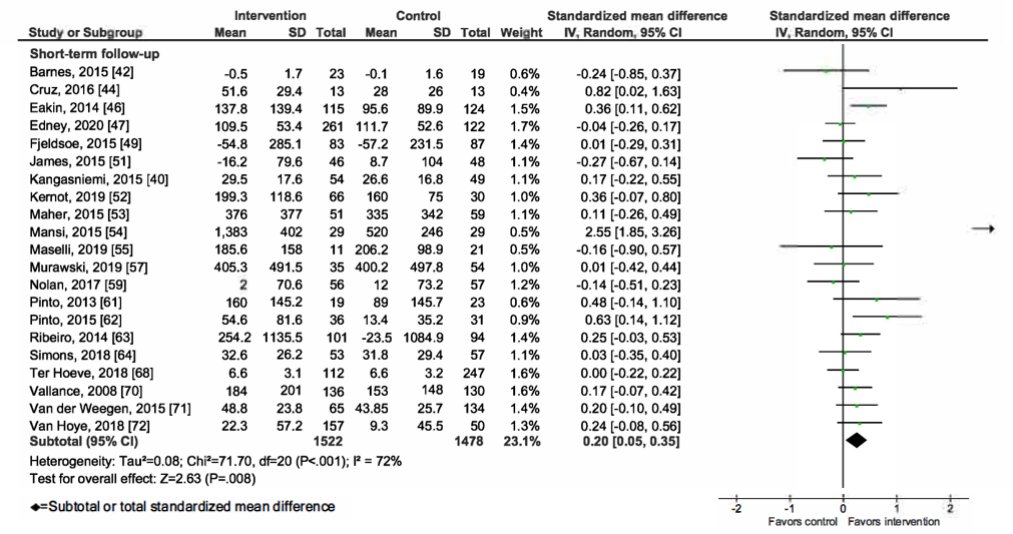
Primary outcome analysis for the outcome moderate-to-vigorous physical activity at timepoint short-term follow-up.

**Figure 7:**
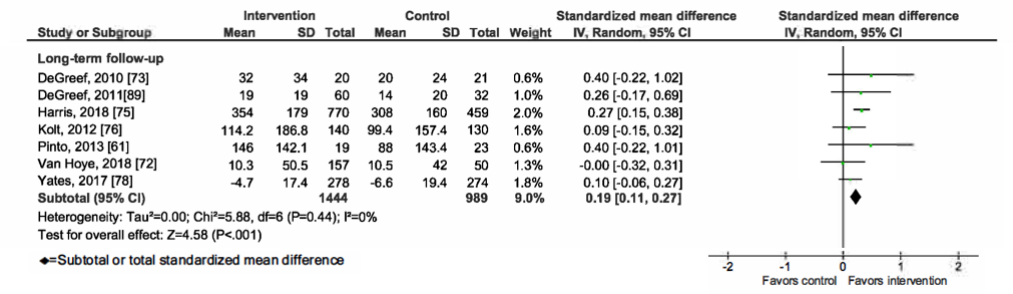
Primary outcome analysis for the outcome moderate-to-vigorous physical activity at timepoint long-term follow-up.

**Figure 8:**
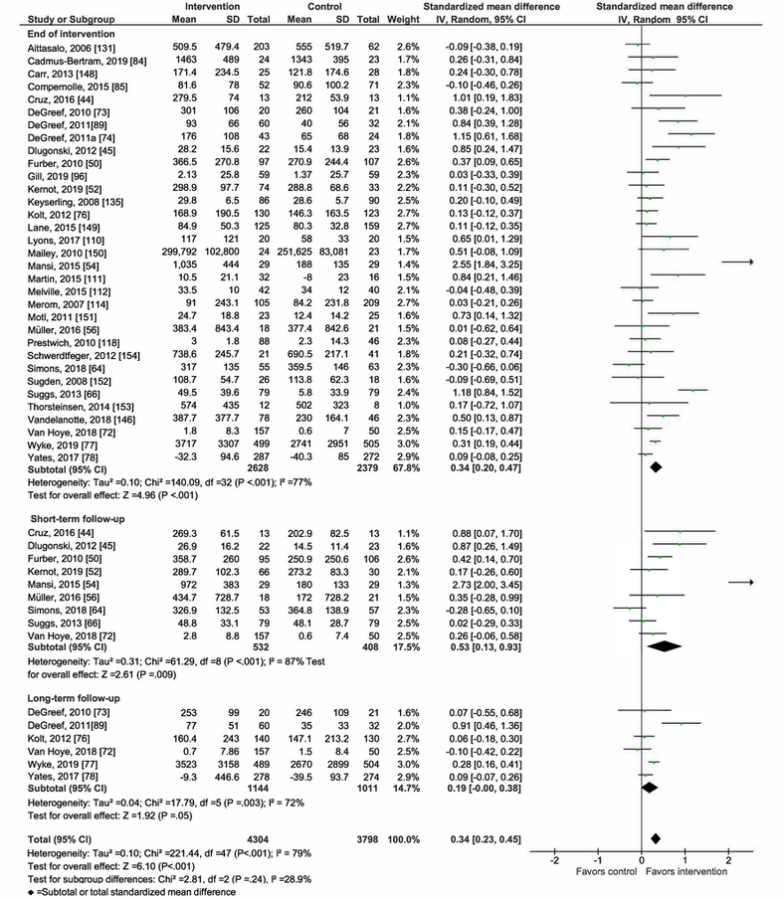
Primary outcome analysis by measurement time point for the outcome total physical activity.

**Figure 9:**
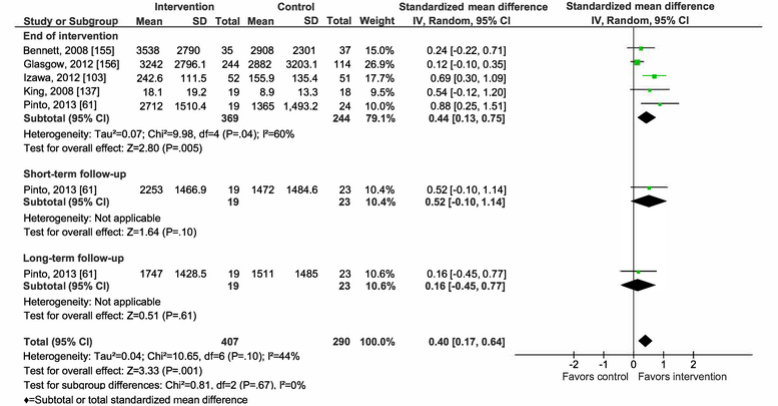
Primary outcome analysis by measurement time point for the outcome energy expenditure.

Overall, mHealth interventions significantly increased PA across all 4 outcome measures at the end of intervention: walking SMD 0.46 (95% CI 0.36-0.55; *P*<.001; *I*^2^=83%, *P*<.001); MVPA SMD 0.28 (95% CI 0.21-0.35; *P*<.001; *I*^2^=62%, *P*<.001); TPA SMD 0.34 (95% CI 0.20-0.47; *P*<.001; *I*^2^=77%, *P*<.001); and EE SMD 0.44 (95% CI 0.13-0.75; *P*=.005; *I*^2^=60%, *P*=.04; Figures 2-9). Short-term effects were sustained (≤6 months after the end of intervention) for 3 of 4 outcome measures: walking SMD 0.26 (95% CI 0.09-0.42; *P*=.002; *I*^2^=73%, *P*<.001); MVPA SMD 0.20 (95% CI 0.05-0.35; *P*=.008; *I*^2^=72%, *P*<.001); and TPA SMD 0.53 (95% CI 0.13-0.93; *P*=.009; *I*^2^=87%, *P*<.001). Only one study [61] reported short-term follow-up measurements for EE, and the results were not statistically significant. In addition, long-term (>6 months after the end of intervention) effects were sustained for 2 of 4 outcome measures: walking SMD 0.25 (95% CI 0.10-0.39; *P*=.001; *I*^2^=68%, *P*=.004) and MVPA SMD 0.19 (95% CI 0.11-0.27; *P*<.001; *I*^2^=0%, *P*=.44). TPA results were sustained, but the effects were just below the significance threshold (SMD 0.19, 95% CI 0.00-0.38; *P*=.05; *I*^2^=72%, *P*=.003). Again, only one study [61] reported long-term follow-up effects for EE, which were not statistically significant. Effect sizes decreased over time, from almost moderate at the end of intervention to small during the long-term follow-up measurement. We found substantial and significant heterogeneity across all outcomes and most time points, with *I*^2^ ranging from 60% to 83% for end-of-intervention measurements (Figures 2-9).

### Publication Bias Assessment

Publication bias was assessed using funnel plot analysis for end-of-intervention measurements, as all but one study [40] reported this time point (Multimedia Appendix 5 [54,82,102]). No systematic publication bias was observed. However, the funnel plot analysis revealed 3 outlier studies [54,82,102]. We identified unusually high adherence rates [54], possibly because the research team and the study participants were based on the same campus, and a short intervention duration (only 4 weeks) [82,102] as potential reasons for the high effect scores in the outlier studies. We conducted a sensitivity analysis and excluded these studies across all outcome measures. All effects were found to be stable, and heterogeneity was substantially reduced (Multimedia Appendix 6).

### Sensitivity Analyses

Sensitivity analysis by risk of bias was conducted for all outcome measures but for EE, as only one study [155] measuring EE classified as low risk of bias. Results at the end of intervention were found to be robust across outcome measures (Multimedia Appendix 6), with effect sizes substantially increasing for walking, MVPA, and TPA to moderate effect sizes. Short- and long-term follow-up effects were not statistically significant when only studies with low risk of bias were included. Heterogeneity increased and remained substantial. Sensitivity analysis of studies reporting long-term follow-up measurements was conducted for all outcome measures but for EE, as only one study [61] measuring EE reported a long-term follow-up measurement. The results across all time points were robust for all outcome measures.

### Subgroup Analysis by Population Type

We used subgroup analysis to evaluate the effect moderators. Table 1 summarizes all results, and Multimedia Appendices 7 [41-154], 8 [41-154], and 9 [41-154] provide detailed forest plots for each analysis. We found that population type moderates the effect of mHealth interventions on PA. The intervention design and control group type were not found to be significant effect moderators. Subgroup analysis by population type revealed statistically significant (*P*≤.10) quantitative subgroup effects for all outcome measures. The treatment effect at the end of intervention was greater in sick populations (walking SMD 0.44, 95% CI 0.29-0.60, *P*<.001, *I*^2^=71%, *P*<.001; MVPA SMD 0.33, 95% CI 0.21-0.45, *P*<.001, *I*^2^=55%, *P*<.001; TPA SMD 0.59, 95% CI 0.36-0.81, *P*<.001, *I*^2^=49%, *P*=.03; Multimedia Appendix 7) than in healthy populations (walking SMD 0.20, 95% CI 0.04-0.35, *P*=.01, *I*^2^=78%, *P*<.001; MVPA SMD 0.14, 95% CI 0.06-0.23, *P*=.001, *I*^2^=15%, *P*=.29; TPA SMD 0.29, 95% CI −0.10 to 0.67, *P*=.14, *I*^2^=85%, *P*<.001; Multimedia Appendix 7). Within the healthy subgroup, summary effects were only statistically significant for the outcome measures walking and MVPA. The results for at-risk populations were mixed. The outcome measures walking and MVPA exhibited high effect scores, similar to the high effect scores of sick populations (walking SMD 0.59, 95% CI 0.42-0.76, *P*<.001, *I*^2^=87%, *P*<.001; MVPA SMD 0.30, 95% CI 0.18-0.43, *P*<.001, *I*^2^=72%, *P*<.001; Multimedia Appendix 7), whereas effect scores for at-risk populations were lower for TPA (SMD 0.21, 95% CI 0.04-0.38; *P*=.02; *I*^2^=78%, *P*<.001). Although heterogeneity was somewhat reduced within most subgroups compared with the overall outcome heterogeneity, it remained high and significant. The covariate distribution between sick, at-risk, and healthy population subgroups was uneven, as fewer studies investigated preventative mHealth PA interventions in healthy populations.

**Table 1:** Summary of subgroup analyses results.

Outcome measure and time point				Studies, n (%)		SMD^a^ (95% CI)		*P* value		Heterogeneity					Test for subgroup differences (*P* value)	
										*I*^2^ (%)		*P* value				
**Population type**																
	**Walking (n=77)**															.003
		Healthy	14 (18)		0.20 (0.04 to 0.35)		.01		78		*<*.001					
		At-risk	30 (39)		0.59 (0.42 to 0.76)		*<*.001		87		*<*.001					
		Sick	33 (42)		0.44 (0.29 to 0.60)		*<*.001		71		*<*.001					
	**MVPA^b^ (n=62)**															.02
		Healthy	12 (19)		0.14 (0.06 to 0.23)		.001		15		.29					
		At-risk	25 (40)		0.30 (0.18 to 0.43)		*<*.001		72		*<*.001					
		Sick	25 (40)		0.33 (0.21 to 0.45)		*<*.001		55		*<*.001					
	**TPA^c^ (n=33)**															.03
		Healthy	6 (18)		0.29 (–0.10 to 0.67)		.14		85		*<*.001					
		At-risk	16 (48)		0.21 (0.04 to 0.38)		.02		78		*<*.001					
		Sick	11 (33)		0.59 (0.36 to 0.81)		*<*.001		49		.03					
**Intervention design**																
	**Walking (n=77)**															.35
		Scalable	31 (40)		0.54 (0.34 to 0.74)		*<*.001		89		*<*.001					
		Nonscalable	45 (58)		0.42 (0.31 to 0.54)		*<*.001		77		*<*.001					
		Combined	1 (1)		0.37 (0.25 to 0.48)		*<*.001		N/A^d,e^		N/A					
	**MVPA (n=62)**															.12
		Scalable	23 (37)		0.20 (0.08 to 0.32)		.001		67		*<*.001					
		Nonscalable	38 (61)		0.33 (0.24 to 0.43)		*<*.001		57		*<*.001					
		Combined	1 (2)		0.37 (0.25 to 0.48)		*<*.001		N/A		N/A					
	**TPA (n=33)**															.60
		Scalable	12 (36)		0.39 (0.06 to 0.73)		.02		89		*<*.001					
		Nonscalable	21 (64)		0.30 (0.18 to 0.42)		*<*.001		53		.002					
		Combined	—^f^		—		—		—		—					
**Control group type**																
	**Walking (n=77)**															.15
		No or minimal intervention	62 (81)		0.47 (0.36 to 0.59)		*<*.001		85		*<*.001					
		Alternative intervention	10 (13)		0.48 (0.12 to 0.83)		.009		80		*<*.001					
		Combined	5 (6)		0.23 (0.01 to 0.45)		.04		62		.03					
	**MVPA (n=62)**															.26
		No or minimal intervention	43 (69)		0.29 (0.21 to 0.38)		*<*.001		67		*<*.001					
		Alternative intervention	9 (15)		0.39 (0.14 to 0.65)		.002		62		.007					
		Combined	10 (16)		0.20 (0.08 to 0.32)		*<*.001		34		.13					
	**TPA (n=33)**															.006
		No or minimal intervention	24 (73)		0.34 (0.19 to 0.50)		*<*.001		76		*<*.001					
		Alternative intervention	6 (18)		0.48 (0.06 to 0.91)		.03		83		*<*.001					
		Combined	3 (9)		0.00 (−0.17 to 0.17)		.97		0		.59					

^a^SMD: standardized mean difference.

^b^MVPA: moderate-to-vigorous physical activity.

^c^TPA: total physical activity.

^d^N/A: not applicable.

^e^In subgroups where n=1, heterogeneity cannot be calculated.

^f^No studies with combined subgroups. Thus, no numbers reported.

### Subgroup Analysis by Intervention Design

Subgroup analysis by intervention design revealed no significant subgroup differences across 3 outcome measures (walking, *P*=.35; MVPA, *P*=.12; TPA, *P*=.60; Table 1; Multimedia Appendix 8) and did not identify mHealth intervention design as a significant effect moderator. Heterogeneity within subgroups was substantial and significant across all outcome measures (Table 1). Both scalable and nonscalable mHealth intervention designs significantly increased PA at similar levels (scalable walking SMD 0.54, 95% CI 0.34-0.74, *P*<.001, *I*^2^=89%, *P*<.001; scalable MVPA SMD 0.20, 95% CI 0.08-0.32, *P*=.001, *I*^2^=67%, *P*<.001; scalable TPA SMD 0.39, 95% CI 0.06-0.73, *P*=.02, *I*^2^=89%, *P*<.001; nonscalable walking SMD 0.42, 95% CI 0.31-0.54, *P*<.001, *I*^2^=77%, *P*<.001; nonscalable MVPA SMD 0.33, 95% CI 0.24-0.43, *P*<.001, *I*^2^=57%, *P*<.001; nonscalable TPA SMD 0.30, 95% CI 0.18-0.42, *P*<.001, *I*^2^=53%, *P*=.002; Multimedia Appendix 8).

### Subgroup Analysis by Control Group Type

Subgroup analysis by control group type found no statistically significant subgroup effect for the outcome measures walking (*P*=.15) and MVPA (*P*=.26). Subgroup differences were only significant for TPA (*P*=.006), where mHealth interventions led to larger effects in studies compared with alternative control groups (SMD 0.48, 95% CI 0.06-0.91; *P*=.03; *I*^2^=83%, *P*<.001; Multimedia Appendix 9) than in studies with no or minimal control groups (SMD 0.34, 95% CI 0.19-0.50; *P*<.001; *I*^2^=76%, *P*<.001; Multimedia Appendix 9). Subgroup analysis did not significantly reduce heterogeneity, and the covariate distribution between the no or minimal intervention subgroup and the alternative intervention subgroup was extremely uneven).

### Risk of Bias in Included Studies

**Figure 10:**
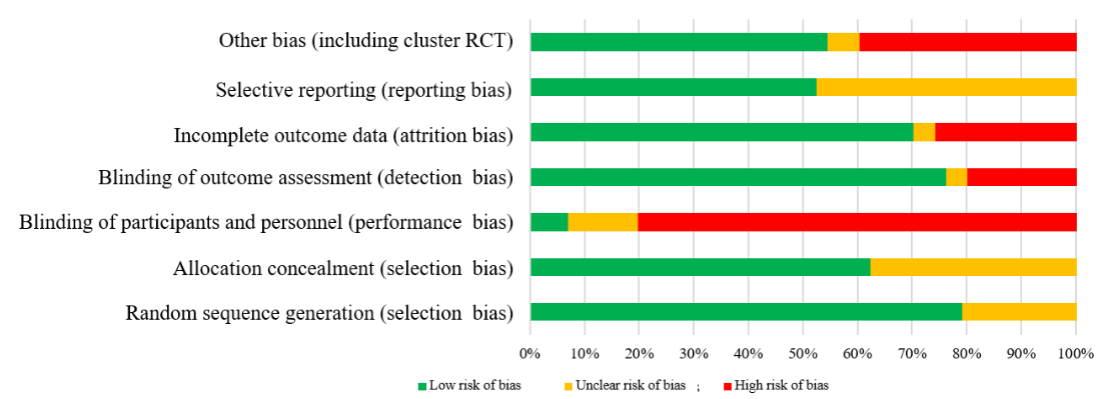
Summary of the overall risk of bias assessment for included studies. RCT: randomized controlled trial.

Figure 10 shows the overall risk of bias assessment across all included studies. Overall, 94 studies were classified as high risk because of selection bias (14/117, 11.9%), detection bias (37/117, 31.6%), attrition bias (42/117, 35.9%), reporting bias (13/117, 11.1%), and other biases (56/117, 47.9%). These mostly included baseline group indifferences or biases resulting from the respective study design (including potential cluster RCT biases) [35]. Multimedia Appendix 10 [40-156] displays the individual risk of bias assessment by study. GRADE analysis of all 4 outcomes (Multimedia Appendix 11) revealed no evidence of publication bias but evidence of inconsistency for the outcome measure EE. Thus, the overall quality of evidence rating ranged from low (walking, MPVA, and TPA) to very low (EE).

## Discussion

### Principal Findings

This systematic review is the most comprehensive study to date of mHealth PA interventions in adult populations. The aims of this study are to understand the long-term impacts of mHealth interventions on PA and to identify important effect moderators.

Overall, our analysis confirms the potential of mHealth interventions to increase PA at the end of intervention. We found small to moderate positive effects (SMD 0.28-0.46), which concur with previous research that reported small to large effect sizes [19,21-24,27,28]. Transforming our results into mean differences based on a representative low risk of bias study [109], we found mHealth interventions to result in 1566 incremental steps per day and an additional 36 minutes of MVPA per week. Previous research found that 1000 incremental steps per day can result in a 10% lower risk of having metabolic syndrome (MetS) and a 6% risk reduction of all-cause mortality, substantiating that mHealth interventions can result in significant health benefits [158,159]. This study is among the first to find that activity increases are sustained beyond the end of intervention. Increased PA levels remained significant in short-term follow-ups taken on average 4.14 months after the end of intervention for the outcome measures walking, MVPA, and TPA. Long-term follow-up measurements, taken on average after 13.96 months, confirmed these results. However, effect sizes decreased over time and ranged from 0.19 to 0.25 at the long-term follow-up time point, which is equivalent to an incremental 851 steps per day and 24 minutes per week of MVPA. Our results concur with the recent review by Chaudhry et al [20], who also found maintained but decreasing effects of step-count monitoring interventions on PA; however, as Chaudhry et al [20] defined time frames from the start of intervention and this study looks at follow-up measurements after the end of intervention, absolute effect scores cannot be compared. Given the inverse relationship of PA with the prevalence of MetS [158], it can be assumed that mHealth interventions still yield health benefits in the long term. These observations are encouraging and provide initial evidence that mHealth interventions can support sustainable behavioral changes. However, our follow-up effects were not robust when only low risk of bias studies were analyzed because of the limited number of high-quality studies with longitudinal designs. Thus, as the current evidence base for studies with long-term follow-up measurements is very limited, further primary research is needed to confirm the sustained effects of mHealth on PA beyond the end of intervention.

Our analysis of effect moderators found that population type moderates the effect of mHealth on PA, whereas intervention design and control group type were not found to be effect moderators. Our evidence suggests that mHealth interventions might be most effective when targeting sick or at-risk populations, thereby supporting the indicative results by Smith et al [27]—effect sizes in sick and at-risk populations were about twice as high as in healthy populations. However, we still found mHealth interventions to be effective in all population types. These results challenge previous findings by Gal et al [23] and Romeo et al [30], who found no differences in effectiveness by population type, likely owing to the small number of studies reviewed. Previous studies found that baseline activity levels are negatively correlated with activity increases in mHealth interventions [144,160,161]. An underlying driver for the higher effectiveness of mHealth interventions in sick and at-risk populations could thus be lower baseline activity levels usually seen within these populations. However, there could also be further underlying factors, such as higher expectations that increases in PA lead to improved health outcomes (outcome expectancy). Further research is thus needed to understand the variety of underlying factors driving higher effectiveness in sick and at-risk populations. Our results provide helpful guidance to policy makers developing scaled-up mHealth intervention programs. Our results suggest that technology-enabled preventative, population-wide programs (eg, *The National Steps Challenge* [14]) might maximize their public health impact if they specifically target at-risk populations (eg, older or overweight groups). Focusing on at-risk groups should also increase the cost-effectiveness of large-scale mHealth programs.

mHealth technologies are cost-effective and scalable. However, this holds true only if technologies are effective without additional nonscalable intervention components (eg, face-to-face coaching). Previous research has found no effects in scalable mHealth intervention designs [24,30], stronger effects in nonscalable designs that combined technology with human-to-human interactions [27,32], and stronger effects when technology was used stand-alone [20]. We found preliminary evidence that mHealth interventions could be effective in scalable intervention designs. Our analysis found no significant subgroup differences between scalable and nonscalable intervention designs, suggesting that both designs can be equally effective in increasing PA. These results are promising and encourage the development of scalable mHealth intervention designs to efficiently increase PA in large population groups. Within our sample, most scalable mHealth interventions leveraged basic technologies (eg, texting, pedometers, or accelerometers), without taking advantage of more advanced mobile technologies (eg, automated individualized coaching, social comparison, and mobile apps), which could have further increased intervention effectiveness [162,163].

Our analysis is among the first to explore whether mHealth PA interventions produce results superior to alternative nonmobile interventions. We found that across the outcome measures walking, MPVA, and TPA, mHealth interventions led to increased levels of PA compared with alternative nonmobile interventions and no or minimal control groups, which accords with previous findings [21]. These results encourage the addition of mHealth technology to nonmobile PA interventions to increase their effectiveness.

### Strengths and Limitations

The strengths of this study are the large number of mHealth interventions analyzed and its rigorous methodology. However, this study has several limitations. First, in line with other studies [164], we encountered large and significant heterogeneity in our results, despite performing several subgroup analyses. Our wide inclusion criteria led us to expect high heterogeneity because of the diverse multicomponent interventions, settings, and intervention durations. In addition, the uneven covariate distribution between subgroups limits the validity of our findings on effect moderators. Second, most of the included studies were classified as having a high risk of bias, and the overall quality of evidence was graded low to very low. The quality of evidence could be improved if future research agreed on standardized reporting of PA outcomes (eg, MVPA in minutes per day) and objective outcome measurement [21]. When replicating our primary results with low risk of bias studies, we could not confirm the effectiveness of mHealth interventions to increase PA beyond the end of interventions, as the available high-quality evidence was limited. Third, we did not attempt to identify unpublished reports or gray literature. Previous research has shown that excluding gray literature might exaggerate the results of a meta-analysis [165]. We tried to mitigate this limitation by conducting a funnel plot analysis to detect potential publication bias. Furthermore, we performed sensitivity analyses to assess the robustness of our results. We detected no systematic publication bias and sensitivity analyses that excluded outlier studies, confirming that our results were robust. Fourth, some studies included in this study allowed intervention participants to keep mHealth devices after the end of intervention. This might have positively skewed the follow-up effects of our review. Finally, although this study provides initial evidence on the long-term effects of mHealth interventions, it only presents results for follow-up measurements taken on average 13.96 months after intervention, and our analysis included only 8 studies. We found that summary effects decrease over time, thus raising the question about the sustainability of positive effects. Further research is required to evaluate whether behavior change—toward a more active lifestyle—is truly sustainable in long term.

### Conclusions

We conclude that mHealth interventions can moderately increase PA in adults at the end of intervention, both compared with alternative nonmobile control groups and no or minimal control groups. PA increases are maintained in follow-up measurements taken after intervention but decrease over time. Population type seems to moderate the effect of mHealth intervention on PA, with higher effectiveness in sick and at-risk populations compared with healthy population samples. mHealth interventions with scalable and nonscalable intervention designs seem to be equivalent in terms of effectiveness. Further high-quality studies investigating scalable mHealth interventions with long-term follow-up measurements are needed to confirm our results. This study concludes that mHealth technologies might not only support sustainable behavior change toward more active lifestyles but also contribute to preventing and controlling chronic disease risk.

## Appendix

Multimedia Appendix 1 Overview of existing meta-analyses on the effect of mobile health interventions on physical activity.

Multimedia Appendix 2 Overview of the search strategy and keywords.

Multimedia Appendix 3 Search algorithms.

Multimedia Appendix 4 Overview of the study characteristics.

Multimedia Appendix 5 Funnel plot analysis to detect publication bias.

Multimedia Appendix 6 Sensitivity analysis.

Multimedia Appendix 7 Subgroup analysis by population type.

Multimedia Appendix 8 Subgroup analysis by intervention design.

Multimedia Appendix 9 Subgroup analysis by control group type.

Multimedia Appendix 10 Study-specific risk of bias judgments.

Multimedia Appendix 11 Grading of recommendations, assessment, development, and evaluation quality of evidence profile.

## Abbreviations

EE: energy expenditure
GRADE: grading of recommendations, assessment, development, and evaluation
MetS: metabolic syndrome
mHealth: mobile health
MVPA: moderate-to-vigorous physical activity
NCD: noncommunicable disease
PA: physical activity
RCT: randomized controlled trial
SMD: standardized mean difference
TPA: total physical activity
WHO: World Health Organization
