# Supplementary material for: Long-term Effectiveness of mHealth Physical Activity Interventions: Systematic Review and Meta-analysis of Randomized Controlled Trials
Source: J Med Internet Res. 2021 Apr 30;23(4):e26699. doi: 10.2196/26699 (PMC8122296; doi:10.2196/26699)
Supplement: Multimedia Appendix 1 [file jmir_v23i4e26699_app1.pdf]

Multimedia Appendix 1. Overview of existing meta-analyses on the effect of mobile health interventions on physical activity.

Review	NOS	N	Inter-vention duration, wks	CGT	Population	Results	Analysis of long-term effects	Subgroup analysis by population type	Subgroup analysis by CGT	Subgroup analysis by mHealth intervention design	Limitations
Bravata et al. (2007) [18]	26	2767	18 (24)	Active	Outpatient, mostly at-risk and sick	Pedometers significantly increase PA by 2491 steps. Significant decrease in BMI and systolic blood pressure.	No	No	No	Yes 10 000 step goal significant predictor of step increase, activity diary predictor, PA counselling no predictor but due to heterogeneity.	Numbers refer to RCTs only. Short duration and small sample size. High heterogeneity in effects, interventions and samples.
Brickwood et al. (2019) [19]	26	3636	20.0 (21.0)	Mixed	Mixed mostly at-risk or sick	Significant increase in daily step count (SMD 0.24, 627 steps), energy expenditure (SMD 0.28, 300kcal), and MVPA (SMD 0.27, 75min per day). No significant effect on sedentary behavior.	No	No	No	Yes Comparison of wearable only (scalable) and multi-faceted (nonscalable) interventions. For walking, both significant positive effects. For MVPA, only the multi-faceted interventions produce significant effects.	Large heterogeneity of samples, interventions.
Chaudhry et al. (2020) [20]	70	12491	NR	Mixed	Mixed, healthy and at-risk; but all community dwelling	Significant short- and long-term PA increases (≤4 months +1126 steps/day; 1 year +464 steps/day). Newer devices (accelerometers, smartphone apps) are less advantageous than simpler pedometers. Adding coaching or financial incentives to step tracking interventions did not lead to further step increases.	Yes At ≤4 months follow-up increases of 1126 steps/day; +1050 at 6mths; +464 steps/day after 1 year; +121 steps/day at 2 years (but non-significant); +434 stpes/day at 3-4 years follow-ups.	No	No	Yes Comparison by type of monitor and intervention intensity. Studies with body-worn trackers or apps reported lower step increases than pedometer interventions (-927 steps/day). Interventions that combined tracking devices with counseling or financial incentives led to lower step increases than simple step tracking interventions (-931 steps/day).	Large degree of heterogeneity, limited sample of studies with long-term follow-ups, sub-group results/meta-regression is a between trial analysis and results are thus less rigorous than within trial comparisons.
De Vries et al. (2016) [21]	14	1157	16.6 (11.6)	Mixed	At-risk, all overweight or obese	Significant increase vs. waitlist control group or usual care for walking (SMD 0.9) and MVPA (SMD 0.5). Significant increase vs. alternative intervention found for walking (MD 282 MET-minutes per week) and energy expenditure (SMD 0.45), no significant effect on MVPA.	No	n/a	Yes, differentiated between waitlist/usual care and alternative intervention.	No	Small sample size, high level of heterogenicity between studies, potential of publication bias.

Appendix 1 Continued

**Review**	**NOS**	**N**	**Inter-vention duration, wks**	**CGT**	**Population**	**Results**	**Analysis of long-term effects**	**Subgroup analysis by population type**	**Subgroup analysis by CGT**	**Subgroup analysis by mHealth intervention design**	**Limitations**
Direito et al. (2017) [29]	21	1701	15.5 (13.7)	Mixed	Mixed, mostly at-risk	No significant differences found for PA, MVPA, walking. Moderate, significant effect on reduction of sedentary behavior (SMD -0.26)	No	No	No	No	Small sample size, limited duration, high levels of heterogeneity, all studies performed in high income countries.
Franssen et al. (2020) [22]	2858	35	22 (17)	Mixed	Sick and at-risk, adults with chronic diseases, obese or elderly adults	Significant increase in PA by 2123 steps/day. Larger effects found in younger populations. Duration and type of intervention (tracker only or combined) had no impact on the effect size.	No	No<br>Analysis by chronic diseases type and risk-group. CVD patients had lower effect compared to other populations.	No	Yes<br>Intervention type (tracker only or tracker combined) had no impact on effect size.	High heterogeneity, inclusion of studies with high risk of bias and small sample sizes, publication bias detected for some populations.
Gal et al. (2018) [23]	18	2734	13.7 (11.6)	Mixed	Mixed, healthy, at-risk and sick	Small to moderate increase in objectively measured PA (SMD 0.43) and moderate increase in objectively measured walking (SMD 0.51). No significant effect found on subjectively measured MVPA.	No	Yes<br>No differences found between healthy and sick study populations.	No	No	Small sample size, potential of publication bias, short duration of studies, large heterogeneity between studies.
Hodkinson et al. (2019) [24]	5208	36	30 (-)	Mixed	Sick & at-risk, adults w/ cardio-metabolic conditions	Significant small to medium effect (walking SMD 0.52, 1703 steps/day; MVPA SMD 0.22) Improvements in PA levels of patients with cardiometabolic conditions.	No<br>Narrative synthesis of long-term follow-up studies.	n/a<br>Adults with cardiometabolic conditions only	No<br>Sensitivity analysis for usual care group.	Yes<br>Face-to-face sessions with facilitators increased PA. No results found for simple self-monitored (ie, scalable) accelerometer interventions.	Heterogeneity amongst studies remained substantial, limiting the validity of the meta-regression results, potential miss of studies not explicitly stating cardiometabolic conditions.
Kirk et al. (2019) [25]	35	4528	20.5 (15.9)	Mixed	Sick, chronic cardio-metabolic diseases	Large effect on walking and small effect on MVPA. Statistically significant increases in walking measured in steps/day (MD 2 592) and MVPA measured in min/week (MD 36.31).	No	n/a	No	Yes<br>Health coaching had no further impact on MVPA or steps/day.	Large heterogeneity between studies.

Appendix 1
Continued

Review	NOS	N	Inter-vention duration, wks	CGT	Population	Results	Analysis of long-term effects	Subgroup analysis by population type	Subgroup analysis by CGT	Subgroup analysis by mHealth intervention design	Limitations
Qiu et al. (2014) [26][a]	7	861	23.9 (15.3)	Mixed	Sick, Type 2 diabetes patients	Significant increase in physical activity (walking) by 1822 steps/day. When step counter was combined with goal setting, effect increased to 3 200 steps/day.	No	n/a	No	Yes<br>mHealth plus goal setting increases effect size.	Potential publication bias, small study sample, large heterogenicity between studies, interventions had multiple components making it difficult to evaluate the contribution of the step counter.
Romeo et al. (2019) [30]	9	1740	13.2 (14.9)	Mini-mal or no-interven tion	Mixed	No significant impact of smartphone apps on PA (MVPA and walking) found. When only interventions of less than 3 months were included, significant effect on walking in steps/day was found (2 075 steps). When apps targeting only PA were used, significant effects on walking in steps/day were found (717 steps).	No	Yes<br>Subgroup analysis performed for healthy vs. sick populations but no significant differences were found. For both groups, results were insignificant.	n/a<br>Only waitlist and minimal intervention.	n/a<br>Only scalable interventions included (smartphone app only).	Small sample, high level of heterogeneity.
Smith et al. (2020) [27]	8742	59	-	Mixed	Mixed	Text message interventions led to significantly greater objectively measured steps/day (SMD 0.38), effects for MVPA were not statistically significant (SMD 0.31). Interventions with more components and interventions in medical populations led to larger effect sizes, however differences were not statistically significant.	No	Yes<br>Text interventions were more effective in sick populations, yet results were not statistically significant.	No	Yes<br>Interventions with more components or tailored text messages were more effective, yet results were not statistically significant.	Small sample, significant heterogeneity in included studies, quality of evidence moderate.
Vaes et al. (2013) [28][a]	8	633	21.1 (15.3)	Mixed	Sick, Patients with diabetes	Large significant effect (SMD 0.81) on walking. Analysis showed that activity monitor-based interventions in combination with counseling resulted in a significantly greater number of steps/day (2 042 steps).	No	n/a<br>Diabetes patients only.	No	No	Small sample, large heterogeneity of study samples in duration and intervention design, among others.

Appendix 1
Continued

Review	NOS	N	Inter-vention duration, wks	CGT	Population	Results	Analysis of long-term effects	Subgroup analysis by population type	Subgroup analysis by CGT	Subgroup analysis by mHealth intervention design	Limitations
Yerra-kalva et al. (2019) [31]	486	6	14.4 (5.4)	Mixed	At-risk, elderly adults	Mobile app interventions may be effective in increasing PA in trials 3 months or shorter and in trials 6 months or longer, but results cannot be concluded with certainty. No statistically significant increases in PA short- and long-term were found, but results indicate short-term and long-term increase (506 and 753 steps/day respectively).	Yes Quantitative analysis of follow-up measure-ments, however very small sample.	No	No	No	Small sample, limited generalizability, review includes non-randomized trials.

Abbreviations: NOS: number of studies included in the review; N: number of participants included in the review, CGT: control group type; MD: mean difference; SMD: standardized mean difference; PA: physical activity; MVPA: moderate to vigorous PA; TPA: total physical activity; EE: energy expenditure. *Comments:* at-risk populations include elderly, sedentary overweight and obese populations. Duration: mean duration and standard deviation of duration of studies included in the respective review. [a]We only display results relevant to PA, as these reviews also cover other health related outcomes.
