# Supplementary material for: Long-term Effectiveness of mHealth Physical Activity Interventions: Systematic Review and Meta-analysis of Randomized Controlled Trials
Source: J Med Internet Res. 2021 Apr 30;23(4):e26699. doi: 10.2196/26699 (PMC8122296; doi:10.2196/26699)
Supplement: Multimedia Appendix 2 [file jmir_v23i4e26699_app2.pdf]

Multimedia Appendix 2. Overview of the search strategy and keywords.

Outcomes	Interventions	Exclusion
physical activity, walking, steps, moderate to vigorous physical activity, moderate-to-vigorous physical activity, exercise, leisure time activity, leisure-time activity, fitness, running, sitting, sedentary, sedentary behavior, sedentary behaviour, inactive, inactivity, active lifestyle, sedentary lifestyle, training, sport*, cycling, bicycle, aerobic*, aerobic exercise, aerobic physical activity	smartphone application, smartphone app, mobile app, mobile application, app, mobile phone, smartphone*, mobile device, PDA, tablet, cell phone, text message*, sms, short message service, mobile health, mHealth, m-health, internet, telehealth, telemedicine, eHealth, e-health iPod, Fitbit, Garmin, Jawbone, Nike, Withings, ambulatory monitoring, ambulatory assessment, wireless technology, accelerometer, pedometer, wearable*, wearable activity tracker, intervention, program*, support, education, therapy	child*, adolescent*, protocol
