# Supplementary material for: Long-term Effectiveness of mHealth Physical Activity Interventions: Systematic Review and Meta-analysis of Randomized Controlled Trials
Source: J Med Internet Res. 2021 Apr 30;23(4):e26699. doi: 10.2196/26699 (PMC8122296; doi:10.2196/26699)
Supplement: Multimedia Appendix 3 [file jmir_v23i4e26699_app3.pdf]

Multimedia Appendix 3. Search algorithms.

PubMed search strategy

((physical activity [Title/Abstract]) OR (steps[Title/Abstract]) OR (exercise[Title/Abstract]) OR (fitness[Title/Abstract]) OR (running[Title/Abstract]) OR (sitting[Title/Abstract]) OR (sedentary*[Title/Abstract]) OR (inactive[Title/Abstract]) OR (inactivity[Title/Abstract]) OR (active lifestyle[Title/Abstract]) OR (training[Title/Abstract])) **AND** ((mobile application[Title/Abstract]) OR (app[Title/Abstract]) OR (mobile phone[Title/Abstract]) OR (smartphone[Title/Abstract]) OR (mobile device[Title/Abstract]) OR PDA[Title/Abstract]) OR (tablet[Title/Abstract]) OR (cell phone[Title/Abstract]) OR (text messag*[Title/Abstract]) OR sms[Title/Abstract]) OR (short message service[Title/Abstract]) OR (mobile health[Title/Abstract]) OR (mHealth[Title/Abstract]) OR (internet[Title/Abstract]) OR (telehealth[Title/Abstract]) OR (telemedicine[Title/Abstract]) OR (eHealth[Title/Abstract]) OR (iPod[Title/Abstract]) OR (Fitbit[Title/Abstract]) OR (accelerometer[Title/Abstract]) OR (pedometer[Title/Abstract]) OR (wearable*[Title/Abstract])) **AND** ((intervention[Title/Abstract]) OR (program*[Title/Abstract]) OR (support[Title/Abstract]) OR (education[Title/Abstract]) OR (therapy[Title/Abstract])) **NOT** ((child*[Title]) OR (adolescent*[Title]) OR (protocol[Title]))

Limitations applied: Clinical Trial, Randomized Controlled Trial, Humans

Cochrane search strategy

(physical activity or steps or exercise or fitness or running or sitting or sedentary* or inactive or inactivity or active lifestyle or training):ti AND (physical activity or steps or exercise or fitness or running or sitting or sedentary* or inactive or inactivity or active lifestyle or training):ab AND (mobile application or app or mobile phone or smartphone or mobile device or PDA or tablet or cell phone or text messag* or sms or short message service or mobile health or mHealth or internet or telehealth or telemedicine or eHealth or iPod or Fitbit or accelerometer or pedometer or wearable*):ti AND (mobile application or app or mobile phone or smartphone or mobile device or PDA or tablet or cell phone or text messag* or sms or short message service or mobile health or mHealth or internet or telehealth or telemedicine or eHealth or iPod or Fitbit or accelerometer or pedometer or wearable*):ab AND (Intervention or program* or support or education or therapy):ti AND (Intervention or program* or support or education or therapy):ab NOT (child* or adolescent* or protocol):ti

Limitations applied: EMBASE database

PsychInfo search strategy

TI (physical activity or steps or exercise or fitness or running or sitting or sedentary* or inactive or inactivity or active lifestyle or training) AND AB (physical activity or steps or exercise or fitness or running or sitting or sedentary* or inactive or inactivity or active lifestyle or training) **AND** TI (mobile application or app or mobile phone or smartphone or mobile device or PDA or tablet or cell phone or text messag* or sms or short message service or mobile health or mHealth or internet or telehealth or telemedicine or eHealth or iPod or Fitbit or accelerometer or pedometer or wearable*) AND AB (mobile application or app or mobile phone or smartphone or mobile device or PDA or tablet or cell phone or text messag* or sms or short message service or mobile health or mHealth or internet or telehealth or telemedicine or eHealth or iPod or Fitbit or accelerometer or pedometer or wearable*) **AND** TI (Intervention or program* or support or education or therapy) AND AB (Intervention or program* or support or education or therapy) **NOT** TI (child* or adolescent* or protocol)

Limitations applied: no limits

Scopus search strategy

((TITLE ("physical activity" OR steps OR exercise OR fitness OR running OR sitting OR sedentary* OR inactivity OR inactive OR "active lifestyle" OR training))) AND ((TITLE ("mobile application" OR app OR "mobile phone" OR smartphone OR "mobile device" OR pda OR tablet OR "cell phone" OR "text messag*" OR sms OR "short message service" OR "mobile health" OR mhealth OR internet OR telehealth OR telemedicine OR ehealth OR ipod OR fitbit OR accelerometer OR pedometer OR wearable*))) AND ((TITLE (intervention OR program* OR support OR education OR therapy))) AND NOT ((TITLE (child* OR adolescent* OR protocol))) AND (LIMIT-TO (EXACTKEYWORD,"Human") OR LIMIT-TO (EXACTKEYWORD,"Humans") OR LIMIT-TO (EXACTKEYWORD,"Controlled Study"))

Limitations applied: Human, Humans, Controlled Trial
