# Supplementary material for: Long-term Effectiveness of mHealth Physical Activity Interventions: Systematic Review and Meta-analysis of Randomized Controlled Trials
Source: J Med Internet Res. 2021 Apr 30;23(4):e26699. doi: 10.2196/26699 (PMC8122296; doi:10.2196/26699)
Supplement: Multimedia Appendix 4 [file jmir_v23i4e26699_app4.pdf]

Multimedia Appendix 4. Overview of the study characteristics.

	Study design, key sample demographics	Outcome	Intervention and control description	Follow-up Measure-ment	Popu-lation type	Ease of Scala-bility
Aittasalo 2006 [131]	RCT; Finland; n=265; Age: 47 (SD 11); 76% female; Duration: 26wks	MVPA & TPA (in min/week; questionnaire)	I: Pedometer & PA-log.<br><br>C: Combined; usual care group and counseling group.	Short-term after 6mths	At-risk	High
Aittasalo 2012 [41]	RCT; Finland; n=241; Age: 44.6 (SD 9.2); 68.4% female; Duration: 24wks	Walking (in min/week; IPAQ)	I: Pedometer use, step-log and monthly e-mail messages from occupational health care provider.<br><br>C: No intervention; waitlist.	-	At-risk	High
Alsaleh 2016 [79]	RCT; Jordan; n=156; Age: 57.8 (SD 9.5); 46.2% female; Duration: 26wks	Walking & MVPA (in min/week; IPAQ)	I: Usual care plus 6-month behavioral intervention by a cardiac nurse, PA diary, 6 telephone consultations, mobile text alerts.<br><br>C: Minimal intervention; information from physician about general benefits of PA.	-	Sick	Low
Ashton 2017 [80]	RCT; Australia; n=50; Age: 22.1 (SD 2); 0% female; Duration: 12wks	Walking (in steps/day vs. baseline; Yamax Digi-Walker SW200); MVPA (in min/week; GLTEQ)	I: Website, wearable device, Facebook support group, face-to-face sessions (group and individual), personalized food and nutrient report, home-based resistance training equipment and portion control tool.<br><br>C: No intervention; waitlist.	-	Healthy	Low
Baker 2008 [81]	RCT; United Kingdom; n=79; Age: 49.2 (SD 8); 79.7% female; Duration: 12wks	Walking (in steps/day; Omron HJ-109E Step-O-Meter)	I: PA consultation, followed by 12-week pedometer-based walking program.<br><br>C: Minimal intervention; instruction to maintain normal walking levels.	-	At-risk	High
Barnes 2015 [155]	RCT; Australia; n=40; Age: 39.1 (SD 4.8); 100% female; Duration: 8wks	MVPA (in % MVPA/time tracked vs. baseline; ActiGraph GT3X and GT3X+)	I: Pedometer, 5-minute mothers and daughter education sessions, 60-minutes PA session.<br><br>C: No intervention; waitlist.	Short-term after 3mths	At-risk	Low
Barwais 2013 [82]	RCT; Australia; n=30; Age: 27.7 (SD 4.1); 33.3% female; Duration: 4wks	Walking & MVPA (in min/week; IPAQ-SF)	I: Personal activity monitor (Gruve Solution), goal setting, motivational emails.<br><br>C: No intervention; waitlist.	-	Healthy	High
Bennett 2008 [156]	RCT; New Zealand; n=72; Age: 57.9 (SD 11.2); 90% female; Duration: 24wks	EE (in kcal/week; CHAMPS)	I: Pedometer, telephone coaching with motivational interviewing.<br><br>C: Alternative intervention; equal number of telephone calls but no motivational interviewing.	-	At-risk	Low
Butler 2004 [83]	RCT; Australia; n=33; Age: 52 (SD 1.2); 84.8% female; Duration: 4wks	Walking (in steps/week; pedometer)	I: Pedometer, information package, goal setting.<br><br>C: Minimal intervention; blinded pedometer, information package, goal setting.	-	At-risk	High
Cadmus-Bertram 2019 [84]	RCT; USA; n=50; Age: 54.4 (SD 11.2); 96% female; Duration: 12wks	Walking (steps/day; ActiGraph); MVPA & TPA (min/week; ActiGraph)	I: Fitbit tracker, educational handbook, in-person coaching, social support, email coaching, monitoring by clinic.<br><br>C: Minimal intervention; educational handbook, emails.	-	Sick	Low
Carr 2013 [148]	RCT; USA; n=66; Age: 37.6 (SD 11.9); 75.4% female; Duration: 24wks	TPA (min/week; PAR)	I: Pedometer, website (step into motion), immediate, individually tailored PA messages generated by a computerized expert system.<br><br>C: Alternative intervention; list of 6 reputable PA websites, email prompts on same schedule as IG.	-	At-risk	High
Coelho 2018 [43]	RCT; Brazil; n=37; Age: 45.9 (SD 16.7); 86.4% female; Duration: 12wks	Walking (in steps/day; Yamax Digi-Walker SW200)	I: Pedometer, individualized daily step target, weekly phone consult.<br><br>C: No intervention; only usual asthma follow-ups and telephone check-ins.	Short-term after 2.8mths	Sick	High

	Study design, key sample demographics	Outcome	Intervention and control description	Follow-up Measure-ment	Popu-lation type	Ease of Scala-bility
Compernolle 2015 [85]	Cluster-RCT; Belgium; n=274; Age: 42 (SD 11); 62.5% female; Duration: 12wks	Walking (in steps/day; Omron HJ-203-ED); MVPA & TPA (in min/week; IPAQ)	I: Pedometer, information booklet, opportunity to request online tailored advice.<br><br>C: No intervention.	-	Healthy	High
Creel 2016 [86]	RCT; USA; n=150; Age: 43.2 (SD 11.2); 84% female; Duration: 26wks	Walking (in steps/day; ActiGraph GT3X); MVPA (in min/week; ActiGraph GT3X)	I: Pedometer, information sheet, printed manual, PA goals, exercise counseling.<br><br>C: Minimal intervention; usual care, educational materials.	-	At-risk	Low
Croteau 2004 [129]	RCT; USA; n=15; Age: 80.7 (SD 7.3); 93.3% female; Duration: 4wks	Walking (in steps/week; Yamax Digi-Walker SW200)	I: Counseling session, pedometer, weekly follow-ups.<br><br>C: No intervention; instruction to continue with daily activities.	-	At-risk	Low
Croteau 2007 [130]	RCT; USA; n=179; Age: 72.8 (SD 8.8); 78.2% female; Duration: 12wks	Walking (in stpes/day; Yamax Digi-Walker SW 200)	I: Counseling, pedometer, self-monitoring.<br><br>C: No intervention; waitlist.	-	At-risk	Low
Cruz 2016 [44]	RCT; Portugal; n=32; Age: 66.4 (SD 8.4); 15.6% female; Duration: 12wks	Walking (in steps/day; ActiGraph GT3X+); MVPA & TPA (in min/day; ActiGraph GT3X+)	I: Pulmonary rehabilitation (exercise training, weekly 90-min psychosocial support and education sessions); PA intervention (pedometer, log diary, goal setting, individual feedback by therapists).<br><br>C: Alternative intervention same pulmonary rehabilitation as IG.	Short-term after 3mths	Sick	Low
Dadaczynski 2017 [87]	RCT; Germany; n=232; -; 35% female; Duration: 6wks	Walking & MVPA (in min /week; IPAQ-SF)	I: Online-based program (goal setting, quizzes, PA challenges, social comparison), Fitbit.<br><br>C: No intervention; waitlist.	-	Healthy	High
De Blok 2006 [88]	RCT; Netherlands; n=21; Age: 64 (SD 11.3); 57.1% female; Duration: 9wks	Walking (in steps/day; Yamax Digi-Walker SW200)	I: Pedometer, 4 individual PA counselling sessions, pulmonary rehabilitation program (exercise training, dietary intervention, psycho-educational modules).<br><br>C: Minimal intervention; usual care, same pulmonary rehabilitation program as IG.	-	Sick	Low
De Greef 2010 [73]	RCT; Belgium; n=41; Age: 61.3 (SD -); 31.7% female; Duration: 12wks	Walking (in steps/day; Yamax Digi-Walker SW200); MVPA & TPA (in min/day; Yamax Digi-Walker SW200)	I: Pedometer, 5 cognitive-behavioral group coaching sessions, pedometer diary.<br><br>C: Minimal intervention; informational materials, usual endocrinologist visits.	Long-term after 9.2mths	Sick	Low
De Greef 2011 [74]	RCT; Belgium; n=92; Age: 62 (SD 9); 31.5% female; Duration: 24wks	Walking (in steps/day; accelerometer); MVPA & TPA (in min/day; IPAQ)	I: Face-to-face session, pedometer, diary, 7 telephone follow-ups.<br><br>C: Minimal intervention; usual diabetes care.	Long-term after 6.5mths	Sick	Low
De Greef 2011 [89]	RCT; Belgium; n=67; Age: 67.4 (SD 9.3); 29.9% female; Duration: 12wks	Walking (in steps/day; Yamax Digi-Walker SW200); MVPA & TPA (in min/day, IPAQ)	I: Pedometer, 3 counselling session (delivered by general practitioner or in groups), diary, goal setting.<br><br>C: No intervention.	-	Sick	Low
Demeyer 2017 [90]	RCT; Belgium, Greece, UK, Switzerland, Netherlands; n=343; Age: 66.5 (SD 8); 36.2% female; Duration: 12wks	Walking & MVPA (in min/day vs. baseline; Dynaport Movemonitor & ActiGraph GT3x)	I: Usual care, tele-coaching intervention (pedometer, one-to-one interview with investigator, automated coaching app, individualized automated goal setting, booklet containing home exercises, weekly text message with activity proposals, telephone contacts when non-compliant or failure to progress.<br><br>C: Minimal intervention; educational materials explaining importance of PA in COPD patients, 10-min fact-to-face discussion with investigator.	-	Sick	Low

	Study design, key sample demographics	Outcome	Intervention and control description	Follow-up Measure-ment	Popu-lation type	Ease of Scala-bility
Dishman 2009 [91]	RCT; USA; n=1442; Age: 36.2 (SD 9.8); 69% female; Duration: 12wks	Walking & MVPA (in MET-hrs/week; IPAQ)	I: Pedometer, individual goal setting, handbook, management endorsement, employee–management steering committees, group and organizational goal setting, environmental prompts.<br><br>C: Minimal intervention; CDC health risk appraisal, monthly newsletter.	-	Healthy	High
Dlugonski 2012 [45]	RCT; USA; n=45; Age: 46.6 (SD 9.7); 86.7% female; Duration: 12wks	TPA (in MET-min/week; GLTEQ)	I: Website with educational videos, pedometer, self-monitoring & goal setting, logbook, CD with instructional videos, 7 individual video coaching sessions.<br><br>C: No intervention; waitlist.	Short-term after 3mths	Sick	Low
Duru 2010 [92]	RCT; USA; n=71; Age: 72.8 (SD 8.1); 100% female; Duration: 8wks	Walking (in steps/week vs. baseline; Yamax Digi-Walker SW200)	I: Goal setting, pedometer self-monitoring, exercise classes.<br><br>C: Alternative intervention; lectures on non-PA topics (e.g. memory loss), exercise classes.	-	At-risk	Low
Eakin 2014 [46]	RCT; Australia; n=302; Age: 58 (SD 8.6); 43.7% female; Duration: 78wks	MVPA (in min/week; ActiGraph)	I: Pedometer, telephone counselling (up to 27 calls), motivational interviewing, digital scale, self-monitoring and goal setting.<br><br>C: Minimal intervention; usual primary care visits, educational brochure.	Short-term after 6mths	Sick	Low
Edney 2020 [47]	RCT; Australia; n=444; Age: 41.3 (SD 11.6); 74.1% female; Duration: 39wks	MVPA (in min/day; GENEActiv)	I: Combined intervention group; Active Team app and a wrist-worn pedometer; or a weekly e-mail and basic app.<br><br>C: Minimal intervention; educational materials.	Short-term after 6mths	At-risk	High
Engel 2006 [93]	RCT; Australia; n=57; Age: 62.4 (SD 7.2); 48.1% female; Duration: 26wks	Walking (in min/week; weekly logbook)	I: Pedometer, coaching (goal setting, education and motivational tactics).<br><br>C: Alternative intervention; same coaching as IG.	-	Sick	Low
Finkelstein 2016 [48]	Cluster-RCT; Singapore; n=800; Age: 35.7 (SD 8.5); 53.7% female; Duration: 26wks	Walking (in steps/day vs. baseline; ActiGraph GT-3x+); MVPA (in min/week vs. baseline; ActiGraph GT-3x+)	I: Combined intervention group; all participants received Fitbit and educational booklets; 1/3rd received charity incentives; 1/3rd received cash incentives.<br><br>C: Minimal intervention; educational materials.	Short-term after 6mths	Healthy	High
Fischer 2019 [132]	RCT; Switzerland; n=288; Age: 42.2 (SD 11.4); 68.4% female; Duration: 26wks	MVPA (in min/day; ActiGraph wGT3X-BT)	I: Telephone coaching, text messages.<br><br>C: Combined; telephone coaching or educational materials.	-	At-risk	Low
Fjeldsoe 2010 [94]	RCT; Australia; n=88; Age: 29.5 (SD 6.2); 100% female; Duration: 12wks	Walking & MVPA (in min/week vs. baseline; AWAS)	I: Face-to-face PA goal setting consultation, a goal setting magnet, 3-5 personally tailored SMS/week, a nominated support person who received two SMS weekly.<br><br>C: Minimal intervention; one face-to-face PA goal setting consultation and educational materials.	-	At-risk	Low
Fjeldsoe 2015 [49]	RCT; Australia; n=263; Age: 31.9 (SD 9.5); 100% female; Duration: 13wks	MVPA (in min/week vs. baseline; ActiGraph GT1M)	I: Face-to-face coaching, individually tailored text messages, telephone coaching, educational materials, website, Facebook group.<br><br>C: Alternative intervention, educational materials, website, Facebook group.	Short-term after 6mths	Healthy	Low
Furber 2010 [50]	RCT; Australia; n=222; Age: 66 (SD 11.1); 29.8% female; Duration: 6wks	Walking & TPA (in min/week; AAS)	I: Pedometer, step calendar for self-monitoring, telephone support including goal setting, informational brochures.<br><br>C: Minimal intervention; informational brochures.	Short-term after 4.5mths	Sick	Low

	Study design, key sample demographics	Outcome	Intervention and control description	Follow-up Measure-ment	Popu-lation type	Ease of Scala-bility
Gell 2015 [95]	RCT; USA; n=87; Age: 47 (SD 11); 100% female; Duration: 24wks	Walking (in steps/day; Omron HJ-720ITC)	I: Informational website, map with 5 suggested walking routes, 3 text messages per week.<br><br>C: Alternative intervention, informational website, map with 5 suggested walking routes.	-	Healthy	High
Gill 2019 [96]	RCT; Canada; n=118; Age: 57.7 (SD 13.5); 78.8% female; Duration: 26wks	Walking (in steps/day vs. baseline; Yamax Digi-Walker SW200); TPA (in MET-min/week vs. baseline; IPAQ)	I: In-person coaching, pedometer, personalized feedback, goal setting, social online community, telephone coaching, smartphone app, virtual coach, website.<br><br>C: No intervention; provided publicly available resources related to healthy lifestyles.	-	At-risk	Low
Glasgow 2012 [156]	RCT; USA; n=463; Age: 58.4 (SD 9.2); 49.8% female; Duration: 52wks	EE (in cals/week; CHAMPS)	I: Combined intervention group; educational website, pedometer, goal setting, self-monitoring, motivational calls, follow-up calls, group coaching.<br><br>C: Alternative intervention; educational website, computer-based health risk appraisal, feedback and recommended preventive care behaviors.	-	Sick	Low
Glynn 2014 [97]	RCT; Ireland; n=139; Age: 44.1 (SD 11.5); 64% female; Duration: 8wks	Walking (in steps/day; smartphone)	I: PA app, goal setting, PA brochure.<br><br>C: No intervention; waitlist.	-	Healthy	High
Golsteijn 2018 [133]	RCT; Netherlands; n=510; Age: 66.4 (SD 7.6); 13% female; Duration: 12wks	MVPA (in min/week; ActiGraph GT3X-BT)	I: Computer-tailored PA advice, pedometer, access to informational website.<br><br>C: No intervention; waitlist.	-	Sick	High
Hardeman 2020 [98]	RCT; United Kingdom; n=1007; Age: 56.1 (SD 9.5); 61.7% female; Duration: 13wks	Walking (in steps/day; ActiGraph GT3X); MVPA (in min/day; ActiGraph GT3X)	I: NHS health check, 5-min.coaching, educational materials, pedometer, step chart.<br><br>C: Minimal intervention; NHS health check.	-	Healthy	Low
Harris 2018 [75]	RCT; United Kingdom; n=1321; Age: 60.8 (SD 3.3); 61.8% female; Duration: 12wks	Walking (in steps/day; ActiGraph GT3X+); MVPA (in min/week; ActiGraph GT3X+)	I: Combined intervention; pedometer, activity diary, informational leaflet, goal setting; or 3-4 in-person consultations with nurse, pedometer, activity diary, informational leaflet, goal setting.<br><br>C: Minimal intervention; usual primary care.	Long-term after 45.2mths	At-risk	Com-bined
Hornikx 2015 [99]	RCT; Belgium; n=30; Age: 67 (SD 6.5); 43.3% female; Duration: 4wks	Walking (in steps/day vs. baseline; Dynaport MoveMonitor)	I: Fitbit ultra, telephone PA counselling (3x per week), goal setting.<br><br>C: Minimal intervention; PA consultation.	-	Sick	Low
Hospes 2009 [100]	RCT; Netherlands; n=39; Age: 62.2 (SD 8.6); 40% female; Duration: 12wks	Walking (in steps/day; Yamax Digi-Walker SW200)	I: Pedometer, 5 counselling sessions with motivational interviewing.<br><br>C: Minimal intervention; usual care.	-	Sick	Low
Houle 2011 [101]	RCT; Canada; n=65; Age: 58.5 (SD 8.5); 21.5% female; Duration: 52wks	Walking (in steps/day; Yamax Digi-Walker NL-2000)	I: Pedometer, activity diary, 5 coaching sessions.<br><br>C: Minimal intervention; standard PA and medication advice at discharge, access to center-based cardiac rehabilitation program or health professional.	-	Sick	Low
Hultquist 2005 [102]	RCT; USA; n=62; Age: 45 (SD 6); 100% female; Intervention duration: 4wks	Walking (in steps/day; New Lifestyles NL-2000)	I: Pedometer, daily step log, goal setting.<br><br>C: Minimal intervention; sealed pedometer, daily step log, goal setting.	-	At-risk	High

	Study design, key sample demographics	Outcome	Intervention and control description	Follow-up Measure-ment	Popu-lation type	Ease of Scala-bility
Izawa 2012 [103]	RCT; Japan; n=126; Age: 59.2 (SD 10.7); 20% female; Duration: 3wks	Walking (in steps/day; Kenz Lifecorder EXa1); EE (in cal/day; Kenz Lifecorder EXa1)	I: Accelerometer, group exercise sessions (5x weekly), dietary and medication advice, self-monitoring.<br><br>C: Alternative intervention; group exercise sessions (5x weekly), dietary and medication advice.	-	Sick	Low
James 2015 [51]	RCT; Australia; n=176; Age: 57 (SD 12); 77.4% female; Duration: 8wks	Walking (in steps/day vs. baseline; Yamax Digi-Walker SW200); MVPA (in min/week vs. baseline; AAS)	I: Pedometer and activity log, 6 educational sessions on nutrition and activity, gymstick for resistance training, goal setting, information about community-based programs and support groups.<br><br>C: No intervention; waitlist.	Short-term after 2.8mths	Sick	Low
Kangasniemi 2015 [40]	RCT; Finland; n=138; Age: 43.5 (SD 5); 82.3% female; Duration: 9wks	Walking (in steps/day; ActiGraph GT1M, GT3X); MVPA (in min/day; questionnaire)	I: Pedometer, PA diary, written feedback, 6 group sessions including goal setting and mindfulness exercises.<br><br>C: Alternative intervention; written feedback on PA, PA diary during measurement periods.	Short-term after 6mths	At-risk	Low
Katzmarzyk 2011 [104]	RCT; USA; n=43; Age: 51.4 (SD 8.2); 83.7% female; Duration: 1wk	Walking (in steps/day; ActiGraph GT3X); MVPA (in min/day; ActiGraph GT3X)	I: Informational brochure, pedometer, 10-min introductory walk and coaching on walking strategies.<br><br>C: Minimal intervention; educational brochure.	-	At-risk	Low
Kawagoshi 2015 [105]	RCT; Japan, n=39; Age: 74.6 (SD 8.4); 11.1% female; Duration 52wks	Walking (in min/day vs. baseline; A-MES accelerometer)	I: Pedometer and pulmonary rehab (education program including lectures about equipment use, nutrition, stress management, relaxation techniques, home exercises and the benefits of PA).<br><br>C: Alternative intervention; pulmonary rehab.	-	Sick	Low
Kendzor 2017 [134]	RCT; USA; n=32; Age: 48.4 (SD 8.1); 25% female; Duration: 4wks	MVPA (in min/day; ActiGraph GT3X)	I: Pedometer, automated personalized informational newsletters, inspirational testimonial, fruit/vegetable snack.<br><br>C: No intervention; waitlist.	-	Healthy	Low
Kernot 2019 [52]	RCT; Australia; n=120; Age: 31.8 (SD 4.5); 100% female; Duration: 6wks.	Walking (in min/week; AAS); MVPA (in min/week; ActiGraph GT3X+); TPA (in total activity counts; ActiGraph GT3X+)	I: Combined group: moms step it up Facebook app, daily PA tip, emails, walking challenge; or pedometer and step-log.<br><br>C: Minimal intervention; PA advice emails.	Short-term after 4.6mths	Healthy	High
Keyserling 2008 [135]	RCT; USA; n=236; Age: 53 (SD 1.2); 100% female; Duration: 52wks	MVPA & TPA (in PAA score; New Leaf PAA)	I: Pedometer, individual and group counselling, counselling calls, reinforcement mailings, information about community support services.<br><br>C: Minimal intervention; informational brochures.	-	Healthy	Low
Kim 2018 [136]	Cluster-RCT; USA; n=187; Age: 20.2 (SD 1.7); 62% female; Duration: 15wks	MVPA (in min/week; ActiGraph Actitrainer)	I: Activity tracker, course on PA and nutrition (1 semester).<br><br>C: Alternative intervention; course on PA and nutrition (1 semester).	-	Healthy	Low
King 2008 [137]	RCT; USA; n=37; Age: 60.2 (SD 7.1); 43.2% female; Intervention duration: 8wks	MVPA (in min/week; CHAMPS); EE (in kcal/kg/week; CHAMPS)	I: Educational materials, PDA, pedometer.<br><br>C: Minimal intervention, educational materials.	-	At-risk	High

	Study design, key sample demographics	Outcome	Intervention and control description	Follow-up Measure-ment	Popu-lation type	Ease of Scala-bility
King 2013 [106]	RCT; USA; n=40; Age: 68.3 (SD 8.2); 72% female; Duration: 17wks	Walking (in min/week vs. baseline; CHAMPS)	I: Virtual advisor, pedometer, accompanied walks, goal setting, monetary incentives to walk (raffle).<br><br>C: Minimal intervention; general health program.	-	At-risk	Low
Koizumi 2009 [138]	RCT; Japan; n=68; Age: 66.5 (SD 4); 100% female; Duration: 12wks	MVPA (in min/day; Kenz Lifecorder)	I: Accelerometer, in person meetings at community center to set step goals and get PA feedback.<br><br>C: No intervention; blinded accelerometer.	-	At-risk	Low
Kolt 2012 [76]	RCT; New Zealand; n=330; Age: 74.1 (SD 6.1); 53.9% female; Duration: 12wks	Walking, MVPA & TPA (in min/week; AHSPAQ)	I: Pedometer, initial face-to-face advice on PA from physician, 3 telephone counselling sessions by trained PA counsellors, goal setting.<br><br>C: Alternative intervention; initial face-to-face advice on PA from physician, 3 telephone counselling sessions by trained PA counsellors, goal setting.	Long-term after 9.2mths	At-risk	Low
Lane 2015 [149]	Cluster-RCT; Ireland; n=402; Age: 52.6 (SD -); 100% female; Intervention duration: 9wks	TPA (in min/week; IPAQ)	I: Pedometer, informational booklets, PA classes, individualized training plans.<br><br>C: Minimal intervention informational booklet on healthy eating.	-	Healthy	Low
Li 2017 [139]	RCT; Canada; n=34; Age: 55.5 (SD 8.6); 82% female; Duration: 8wks	MVPA (in MET-in/day; SenseWear Mini)	I: Group education session about PA, Fitbit Flex, individual weekly activity counselling with a physical therapist by telephone.<br><br>C: No intervention; delayed group.	-	Sick	Low
Li 2020 [107]	RCT; Canada; n=118; Age: 53.3 (SD 13.6); 89% female; Duration: 8wks	Walking (in steps/day; SenseWear) MVPA (in min/day; SenseWear)	I: Education and counselling, Fitbit, web-application, feedback, 4 follow-up calls.<br><br>C: Minimal intervention; monthly emails.	-	Sick	Low
Long 2013 [108]	RCT; United Kingdom; n=89; Age: 47.3 (SD 7); 100% female; Duration: 16wks	Walking (in steps/week; New Lifestyles NL 1000)	I: PA consultation by practitioner (including booklet), pedometer, weekly prompts (telephone, e-mail or texts).<br><br>C: Minimal intervention; advisory leaflet.	-	At-risk	Low
Lynch 2019 [109]	RCT; Australia; n=83; Age: 61.6 (SD 6.4); 100% female; Duration: 8wks	Walking (in steps/day; ActivPAL); MVPA (in min/week; ActiGraph GT3X+)	I: Garmin Vivofit 2 activity monitor, feedback, goal-setting session, telephone coaching sessions, app, individualized automated feedback.<br><br>C: No intervention; waitlist.	-	Sick	Low
Lyons 2017 [110]	RCT; USA; n=40; Age: 61.5 (SD 5.6); 85% female; Duration: 12wks	Walking (in steps/day; ActivPAL); TPA (in stepping-min/day; ActivPAL)	I: Mini tablet mobile device, wearable activity monitor, app, orientation visit, weekly telephone counselling.<br><br>C: No intervention; waitlist.	-	At-risk	Low
Maher 2015 [53]	RCT; Australia; n=110; 74.5% female; Duration: 8wks	Walking & MVPA (in min/week; AAS)	I: Pedometer, Facebook app (active team) used in group of 3-8 Facebook friends, calendar to log daily (team) step counts, dashboard, team tally board, social comparison, gamification features.<br><br>C: No intervention; waitlist	Short-term after 2.8mths	At-risk	High
Mailey 2010 [150]	RCT; USA; n=51; Age: 25 (SD -); 68.1% female; Duration: 10wks	TPA (in activity counts/day; ActiGraph)	I: Pedometer, website, activity log, reminder email to submit an activity log, 2 monthly meetings with PA counsellor.<br><br>C: Minimal intervention; mental health counseling, waitlist.	-	Sick	Low

	Study design, key sample demographics	Outcome	Intervention and control description	Follow-up Measure-ment	Popu-lation type	Ease of Scala-bility
Mansi 2015 [54]	RCT; New Zealand; n=58; Age: 41.5 (SD 13.6); 58.6% female; Duration: 12wks	Walking (in steps/day; Yamax Digi-Walker SW200); MVPA & TPA (in MET-min/week; IPAQ-SF)	I: Pedometer, goal setting, feedback, educational material, self-monitoring.<br><br>C: Minimal intervention; educational materials.	Short-term after 3mths	At-risk	High
Martin 2015 [111]	RCT; USA; n=48; Age: 58 (SD 8); 46% female; Duration: 5wks	Walking (in steps/day vs. baseline; Fitbug Orb); TPA (in min/day vs. baseline; Fitbug Orb)	I; Fitbug orb+, smartphone and web interface, smart texts (half of group).<br><br>C: Minimal intervention; blinded Fitbug Orb.	-	Sick	High
Maselli 2019 [55]	RCT; Italy; n=33; Age: 22 (SD 2); 60.6% female; Duration: 12wks	MVPA (in min/week; ActiGraph)	I: MyWellness Key accelerometer, goal setting.<br><br>C: Combined; counseling and goal setting or waitlist.	Short-term after 3mths	At-risk	High
Maxwell-Smith 2019 [140]	RCT; Australia; n=68; Age: 64.1 (SD 7.9); 50% female; Duration: 12wks	MVPA (in min/week; ActiGraph GT9X)	I: Wearable tracker, group coaching, supportive phone calls.<br><br>C: Minimal intervention; educational materials.	-	Sick	Low
Melville 2015 [112]	Cluster-RCT; United Kingdom; n=102; Age: 46.2 (SD 13.0); 44% female; Duration: 12wks	Walking (in steps/day; ActiGraph GT3X); MVPA & TPA (in percentage time/day; ActiGraph GT3X)	I: Pedometer, individual PA consultations, structured walking program, booklet, goal setting, choice-based involvement of advisors.<br><br>C: No intervention; waitlist.	-	Sick	Low
Mendoza 2015 [113]	RCT; Chile; n=102; Age: 68.6 (SD 8.5); 39.2% female; Duration: 12wks	Waling (in steps/day vs. baseline; Tanita PD724)	I: Pedometer, daily diary, regular monthly COPD visit.<br><br>C: Alternative intervention; received counselling at each COPD visit and were advised to walk for at least 30-min per day, diary.	-	Sick	High
Merom 2007 [114]	RCT; Australia; n=369; Age: 49.1 (SD 9.3); 85% female; Duration: 12wks	Walking, MVPA & TPA (in min/week vs. baseline; AAS)	I: Pedometer, brochure, diary, booklet.<br><br>C: Combined; self-help walking program and weekly diaries; or no intervention.	-	At-risk	High
Motl 2011 [151]	RCT; USA; n=54; Age: 45.8 (SD 9.7); 89.6% female; Duration: 12wks	TPA (in MET-min/week; GLTEQ)	I: Informational video, text messages, pedometer, automated email announcements about new information, chats with PA coaches.<br><br>C: No intervention; waitlist.	-	Sick	Low
Müller 2016 [56]	RCT; Malaysia; n=43; Age: 63.3 (SD 4.5); 74% female; Duration: 12wks	TPA (in MET-min/week; IPAQ)	I: Exercise booklet, personal exercise instruction, 60 text messages, home visits.<br><br>C: Minimal intervention; exercise booklet, personal exercise instructions.	Short-term after 2.8mths	At-risk	Low
Murawski 2019 [57]	RCT; Australia; n=160; Age: 41.7 (SD 9.9); 80.0% female; Duration: 12wks	MVPA (in min/week; AAS)	I: Balanced smartphone app, personalized feedback, educational materials, text messages, emails, pedometer.<br><br>C: No intervention; waitlist.	Short-term after 3.2mths	Healthy	High
Mutrie 2012 [58]	RCT; United Kingdom; n=41; Age: 70.8 (SD 5.2); 68.3% female; Duration: 12wks	Walking (in steps/day; ActivPAL)	I: PA consultations, walking program (booklet and pedometer), option to participate in walking groups.<br><br>C: No intervention; waitlist.	Short-term after 2.8mths	At-risk	Low
Nolan 2017 [59]	RCT; United Kingdom; n=152; Age: 68 (SD 9); 28% female; Duration: 8wks	Walking (in steps/day vs. baseline; SenseWear); MVPA (in MET-min/day vs. baseline)	I: Pulmonary rehabilitation, pedometer, step-count diary.<br><br>C: Minimal intervention; pulmonary rehabilitation.	Short-term after 6mths	Sick	High

	Study design, key sample demographics	Outcome	Intervention and control description	Follow-up Measure-ment	Popu-lation type	Ease of Scala-bility
Oliveira 2019 [60]	RCT; Australia; n=131; Age: 71.5 (SD 6.5); 71% female; Duration: 26wks	Walking (in steps/day; ActiGraph GT3X-BT)	I: Fact-to-face health coaching, continuous telephone coaching, goal setting, pedometer, individualized advice, educational materials.<br><br>C: Minimal intervention; educational materials.	Short-term after 6mths	At-risk	Low
Paul 2016 [115]	RCT; United Kingdom; n=24; Age: 56 (SD 10.0); 52% female; Intervention duration: 6wks	Walking (in steps/day; ActivPAL)	I: Smartphone, Starfish app, pedometer.<br><br>C: Minimal intervention; usual care.	-	Sick	High
Pekmezi 2017 [141]	RCT; USA; n=84; Age: 57 (SD 4.7); 100% female; Duration: 26wks	MVPA (in min/week; ActiGraph GT3X)	I: Pedometer, regular mailings, computer generated individualized feedback-reports addressing psychosocial and environmental factors affecting PA.<br><br>C: Minimal intervention; cancer prevention, information on topics other than PA.	-	At-risk	High
Pinto 2013 [61]	RCT; USA; n=46; Age: 57.3 (SD 9.7); 54% female; Duration: 12wks	MVPA (in in/week; 7-day PAR); EE (in cals/week; CHAMPS)	I: Pedometer, walking log, weekly phone calls.<br><br>C: Alternative intervention; weekly phone calls.	Short-term after 3mths, Long-term after 9mths	Sick	Low
Pinto 2015 [62]	RCT; USA; n=76; Age: 55.6 (SD 9.6); 100% female; Duration: 12wks	MVPA (in min/week; ActiGraph GT3X)	I: Telephone-based PA counseling, pedometer, heart rate monitor, information booklets, frequent feedback reports.<br><br>C: Alternative intervention, 12 calls, informational booklets, PA tip sheets.	Short-term after 2.8mths	Sick	Low
Poirier 2016 [116]	RCT; USA; n=265; Age: 39.9 (SD 11.7); 66% female; Duration: 6wks	Walking (in steps/day; Pebble+)	I: Activity tracker, internet-based program (Walkadoo).<br><br>C: Minimal intervention; asked to continue usual routine.	-	Healthy	High
Pope 2018 [117]	RCT; USA; n=32; Age: 52.6 (SD 9.3); 100% female; Duration: 10wks	Walking (in steps/day; ActiGraph GT3X+); MVPA (in min/day; ActiGraph GT3X+)	I: Polar M400 smartwatch, Facebook page, strength and aerobic training program via Facebook.<br><br>C: Alternative intervention; Facebook group, strength and aerobic training program via Facebook, weekly tips.	-	Sick	Low
Prestwich 2009 [142]	RCT; United Kingdom; n=155; Age: 23.8 (SD 4.6); 58% female; Duration: 4wks	MVPA (in exercise frequency, questionnaire)	I: Motivational text messages, implementation intention plan.<br><br>C: Combined; motivational messages, implementation intention plan; or no intervention.	-	At-risk	High
Prestwich 2010 [118]	RCT; United Kingdom; n=149; Age: 23.4 (SD 5.6); 66% female; Duration: 4wks	Walking (in days/week w/>30min; SWET); TPA (in days/week w/>30min exercise; SWET)	I: PA guidelines, implementation intention plan, website, specific PA plan, SMS reminders (on goals or plan).<br><br>C: Minimal intervention; PA guidelines.	-	At-risk	High
Reijonsaari 2012 [119]	RCT; Finland; n=544; Age: 43.5 (SD 10); 64% female; Duration: 52wks	Walking (in MET-min/week; IPAQ-SF)	I: Accelerometer, goal setting, online service helping to track their activity levels, telephone or web counselling.<br><br>C: Minimal intervention; fitness test informational leaflet on PA.	-	Healthy	Low
Ribeiro 2014 [63]	RCT; Brazil; n=195; Age: 45 (SD 3); 100% female; Duration: 13wks	Walking (in steps/week vs. baseline; Digi-Walker PW610); MVPA (in moderate intensity steps/week vs. baseline; Digital-Walker PW 610)	I: Counseling sessions (individual or group), booklet, pedometer, diary.<br><br>C: Combined; counseling sessions, booklet; or aerobic exercise sessions (twice per week).	Short-term after 3mths	At-risk	Low

	Study design, key sample demographics	Outcome	Intervention and control description	Follow-up Measure-ment	Popu-lation type	Ease of Scala-bility
Roos 2014 [120]	RCT; South Africa; n=84; Age: 39.1 (SD 9.2); 79% female; Duration: 52wks	Walking (in steps/day vs. baseline; Yamax Digi-Walker SW200)	I: Pedometer, activity diary, contact sessions, SMS.<br><br>C: Minimal intervention; standard HIV care, monthly call from principal investigator.	-	Sick	Low
Rowley 2019 [121]	RCT; USA; n=170; Age: 67.3 (SD 6.3); 79.4% female; Duration: 12wks	Walking (in steps/day; Omron HJ-720ITC)	I: Combined; individually tailored feedback through interactive website, goal setting, pedometer; or pedometer and goal setting.<br><br>C: No intervention; instruction to maintain current behavior.	-	At-risk	High
Samuels 2011 [143]	RCT; USA; n=50; Age: 48.7 (SD 9.1); 81% female; Duration: 4wks	MVPA (in min/day; ActiGraph 7164)	I: Pedometers, instructed to achieve 10 000 steps daily, weekly meetings, activity logs.<br><br>C: Combined; sealed pedometers, weekly meetings with principal investigator, activity logs, instructed to engage in 30-minutes of MVPA daily; or instructed to accumulate 30-minutes of MVPA in bouts of at least 10-minutes or longer on a daily basis.	-	At-risk	Low
Schwerdt-feger 2012 [154]	RCT; Austria; n=63; Age: 23.7 (SD 4); 68% female; Duration: 1wk	TPA (in counts/min; ActiGraph GT1M)	I: Individual PA information and planning sessions, goal setting, daily SMS reminders (only half of participants).<br><br>C: Minimal intervention: government PA guidelines.	-	At-risk	Low
Sharp 2016 [144]	RCT; Canada; n=184; Age: 18 (SD 0.7); 53% female; Duration: 12wks	MVPA (in min/week, GLTEQ)	I: Pedometer, monthly tracking logs, follow-up e-mails.<br><br>C: No intervention; instruction to continue with usual activity.	-	Healthy	High
Simons 2018 [64]	Cluster-RCT; Belgium; n=130; Age: 25 (SD 3.0); 51.5% female; Duration: 9wks	Walking (in steps/day; ActiGraph GT3X+); MVPA & TPA (in min/day; ActiGraph GT3X+)	I: Active Coach app, Fitbit, goal setting, educational materials.<br><br>C: Minimal intervention; educational materials.	Short-term after 3.2mths	At-risk	High
Spence 2009 [122]	RCT; Canada; n=63; -; 100% female; Duration: 1wk	Walking (in min/week; IPAQ-SF)	I: Pedometer, log sheet, walking intentions and self-efficacy questionnaire encouraging 12 500 steps/day (only half of participants).<br><br>C: Combined; walking intentions and self-efficacy questionnaire encouraging 12 500 steps/day; or no intervention.	-	Healthy	High
Stacey 2016 [65]	RCT; Australia; n=174; Age: 57 (SD 12); 77% female; Duration: 8wks	Walking (in steps/day; Yamax Digi-Walker SW200)	I: Group education sessions, workbook, pedometer, gymstick.<br><br>C: No intervention; waitlist.	Short-term after 2.8mths	Sick	Low
Sugden 2008 [152]	RCT; United Kingdom; n=54; Age: 76 (SD -); 100% female; Intervention duration: 12wks	TPA (in activity count/day; Stay Healthy RT3)	I: Pedometer, individualized activity action plans, daily activity diary, counseling session and phone calls.<br><br>C: Alternative intervention; individualized activity action plans, daily activity diary, counseling session and phone calls.	-	At-risk	Low
Suggs 2013 [66]	RCT; United Kingdom; n=331; Age: 39.5 (SD 14.8); 82.5% female; Duration: 12wks	TPA (in MET hrs/week; IPAQ-L)	I: 12 Emails, 24 text messages.<br><br>C: Alternative intervention; 12 emails.	Short-term after 0.9mths	Healthy	High
Tabak 2014 [123]	RCT; Netherlands; n=34; Age: 66.6 (SD 7.4); 37% female; Duration: 3wks	Walking (in steps/ay; Yamax Digi-Walker SW200)	I: Activity coach (smartphone and accelerometer), web portal, feedback text messages, usual COPD rehabilitation care (medication and physiotherapy).<br><br>C: Minimal intervention; usual COPD rehabilitation care (medication and physiotherapy).	-	Sick	High
Talbot 2003 [67]	RCT; USA; n=34; Age: 70.2 (SD 5.8); 76.5% female; Duration: 12wks	Walking (in steps/day; Yamax Digi-Walker SW200)	I: Arthritis self-management education, pedometer, activity logs, brief individual counseling, exercise booklet.<br><br>C: Alternative intervention; 12-hour arthritis self-management program.	Short-term after 2.8mths	Sick	Low

	Study design, key sample demographics	Outcome	Intervention and control description	Follow-up Measure-ment	Popu-lation type	Ease of Scala-bility
Ter Hoeve 2018 [68]	RCT; Netherlands; n=731; Age: 58.7 (SD 8.7); 18% female; Duration: 52wks	Walking (in steps/wear time; ActiGraph); MVPA (in MVPA/wear time; Actigraph)	I: Pedometer, group exercise and counseling sessions, booklet.<br><br>C: Combined; group exercise sessions; or exercise sessions and 9 month telephonic after-care program.	Short-term after 6mths	Sick	Low
Thorndike 2014 [124]	RCT; USA; n=103; Age: 29 (SD -); 54% female; Duration: 12wks	Walking (in steps/day; Fitbit)	I: Fitbit, Fitbit website.<br><br>C: No intervention; blinded Fitbit.	-	Healthy	High
Thorsteinsen 2014 [153]	RCT; Norway; n=21; Age: 55.3 (SD 11.2); 47.6% female; Duration: 12wks	TPA (in min/week; questionnaire)	I: Information meeting, website, goal setting, personalized text messages, game elements (competition and social comparison, group goal setting).<br><br>C: Minimal intervention; information meeting, daily reporting of PA levels through survey.	-	Healthy	High
Tudor-Locke 2004 [69]	RCT; USA; n=60; Age: 52.7 (SD 5.2); 45% female; Duration: 16wks	Walking (in steps/day; Yamax Digi-Walker SW200)	I: Group meetings, pedometers, program manual, calendars, postcards.<br><br>C: No intervention; waitlist.	Short-term after 1.8mths	Sick	Low
Unick 2012 [145]	RCT; USA; n=29; Age: 42.3 (SD 9.8); 82% female; Duration: 26wks	MVPA (in min/week; SenseWear)	I: Body media FIT system (mobile tracker, website), group meetings, exercise and nutrition goals, paper diaries, feedback.<br><br>C: Alternative intervention; group meetings, exercise and nutrition goals, paper diaries, feedback.	-	Sick	Low
Vallance 2008 [70]	RCT; Canada; n=377; Age: 58 (SD -); 100% female; Duration: 12wks	MVPA (in min/week; GLTEQ)	I: Pedometer, step calendar, PA guidebook (only half of participants), instruction to perform 30-min MVPA 5x per week.<br><br>C: Combined; instruction to perform 30min MVPA 5x per week; or instructions plus PA guidebook.	Short-term after 6mths	Sick	High
Vallance 2016 [125]	RCT; Canada; n=95; Age: 52.8 (SD 9.8); 100% female; Duration: 13wks	Walking (in steps/day; StepsCount SC-01); MVPA (in min/week; GLTEQ)	I: Informational materials, pedometer, activity diary, goal setting.<br><br>C: Minimal intervention; informational materials.	-	Sick	High
Van Blarigan 2019 [126]	RCT; USA; n=42; Age: 54 (SD 11); 58.5% female; Duration: 21wks	Walking (in steps/day ActiGraph GTX3+); MVPA in min/day; ActiGraph GTX3+)	I: Print materials on A after cancer, Fitbit Flex, daily text messages.<br><br>C: Minimal intervention; print materials on PA after cancer.	-	Sick	High
Vandelanotte 2018 [146]	RCT; Australia; n=243; Age: 51.5 (SD 11.1); 74.9% female; Duration: 13wks	MVPA & TPA (in min/week; AAS)	I: Fitbit; Taylor Active web-based intervention, educational materials, goal setting, individualized feedback.<br><br>C: Alternative intervention; TaylorActive web-based intervention, educational materials, goal setting, individualized feedback.	-	At-risk	High
Van der Weegen 2015 [71]	Cluster-RCT; Netherlands; n=199; Age: 57.8 (SD 7.7); 51.2% female; Duration: 26wks	MVPA (in min/day; Personal Activity Monitor AM300)	I: It's LiFe! smartphone and web-app, monitoring, self-management support program including feedback, booklet, and consultation sessions.<br><br>C: Combined; self-management support program including feedback, booklet, and consultation sessions; or usual primary care.	Short-term after 3mths	Sick	Low
Van Hoye 2018 [72]	RCT; Belgium; n=227; Age: 42.4 (SD 10.4); 17% female; Duration: 4wks	Walking (in steps/day vs. baseline; SenseWear); MVPA (in min/day vs. baseline; SenseWear); TPA (in METs/day vs. baseline; SenseWear)	I: Combined intervention group; 1/3rd pedometer & feedback, 1/3rd feedback on daily steps, daily minutes of MVPA and total daily EE in real-time from a SWA display; 1/3rd feedback on daily steps, daily minutes of MVPA and total daily EE in real-time from a SenseWear display plus weekly face-to-face coaching.<br><br>C: Minimal intervention; information on the energy expenditure of familiar activities (e.g. housework, walking, cycling).	Short-term after 6mths, Long-term after 12mths	At-risk	Low

	Study design, key sample demographics	Outcome	Intervention and control description	Follow-up Measure-ment	Popu-lation type	Ease of Scala-bility
Warren 2014 [127]	RCT; United Kingdom; n=131; Age: 59.9 (SD 9.3); 33% female; Duration: 12wks	Walking (in steps/day; New Lifestyles VL-800)	I: Pedometer, informational booklet, PA advice by general practitioner to walk at least 1 mile a day, self-monitoring.<br><br>C: Combined; informational booklet and sealed pedometer; or PA advice by general practitioner to walk at least 1 mile a day and sealed pedometer.	-	At-risk	High
Wijsman 2013 [147]	RCT; Netherlands; n=235; Age: 64.8 (SD 2.9); 41% female; Duration: 12wks	MVPA (in min/day; GENEActiv)	I: Web-based physical activity program (accelerometer and monitor, personal website and eCoach).<br><br>C: No intervention; waitlist	-	At-risk	Low
Wyke 2019 [77]	RCT; England, Netherlands, Norway, Protugal; n=1113; Age: 45.8 (SD 8.8); 0% female; Duration: 12wks	Walking (in steps/day; ActivPAL); TPA (in MET-min/week; IPAQ-SF)	I: Educational materials, group coaching at soccer club, pocket-worn tracking device (SitFIT), game-based app (MatchFIT).<br><br>C: Minimal intervention; educational materials.	Long-term after 9.2mths	At-risk	Low
Yamada 2012 [128]	RCT; Japan; n=87; Age: 75.6 (SD 6.7); 46% female; Duration: 26wks	Walking (in steps/day; Yamax Power-Walker EX-510)	I: Pedometer, step log grid, feedback.<br><br>C: No intervention.	-	At-risk	High
Yates 2017 [78]	Cluster-RCT; United Kingdom; n=808; Age: 63.1 (SD 8.2); 36.4% female; Duration: 104wks	Walking (in steps/day vs. baseline; ActiGraph GT3X); MVPA (in min/day vs. baseline; ActiGraph GT3X); TPA (in 1000 counts/day vs. baseline; ActiGraph GT3X)	I: Pedometer, action plan and step diary, yearly group sessions, telephone contact.<br><br>C: Minimal intervention; informational booklet.	Long-term after 12mths	At-risk	Low

Abbreviations: RCT, randomized controlled trial; SD, standard deviation; MVPA, moderate to vigorous physical activity; TPA, total physical activity; EE, energy expenditure; PA, physical activity; GLTEQ, Godin Leisure-Time Exercise Questionnaire; IPAQ-L or IPAQ, International Physical Activity Questionnaire long-form; IPAQ-SF, International Physical Activity Questionnaire short-form; AAS, Active Australia Survey, PAR, 7-day Physical Activity Recall; CHAMPS, Community Health Activities Model Program for Seniors; SWET, Self-report Walking and Exercise Tables; AWAS, Australian Women's Activity Study; AHSPAQ, Auckland Heart Physical Activity Questionnaire; CDC, Center for Disease Control and Prevention; NHS, National Health Service; COPD, Chronic Obstructive Pulmonary Disease; MET, metabolic equivalent of task .

Comments: At-risk group includes elderly, obese, and inactive populations; sick group includes populations that currently experienced or previously experienced illnesses such as diabetes, cancer, chronic obstructive pulmonary disease, coronary heart disease or others; High ease of scalability defined as the ability to scale-up an intervention without human resource requirement; short-term follow up includes follow ups performed ≤6mths after end of intervention; long-term follow-up defined as measurements taken >6mths after end of intervention.
