# Supplementary material for: Long-term Effectiveness of mHealth Physical Activity Interventions: Systematic Review and Meta-analysis of Randomized Controlled Trials
Source: J Med Internet Res. 2021 Apr 30;23(4):e26699. doi: 10.2196/26699 (PMC8122296; doi:10.2196/26699)
Supplement: Multimedia Appendix 5 [file jmir_v23i4e26699_app5.pdf]

# Multimedia Appendix 5: Funnel plot analysis to detect publication bias.

**Studies reporting Walking as outcome (N=77)**

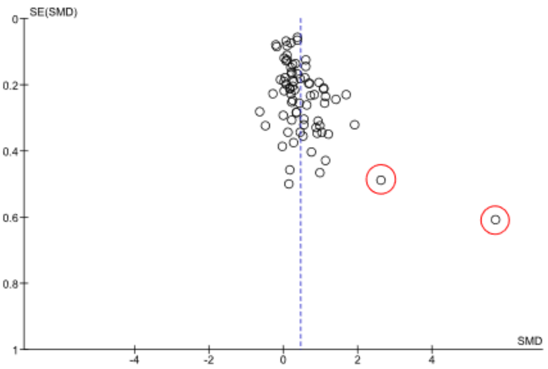


*No publication bias detected apart from outlier.*

*Outlier was visually identified and marked in red: Barwais et al., 2013*[82]*; Hultquist et al., 2013.*[102]

**Studies reporting MVPA as outcome (N=62)**

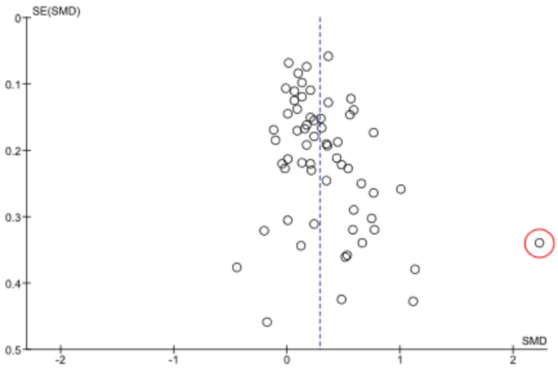


*No publication bias detected apart from outliers.*

*Outliers were visually identified and marked in red: Mansi et al., 2015.*[54]

**Studies reporting Total Physical Activity as outcome (N=33)**

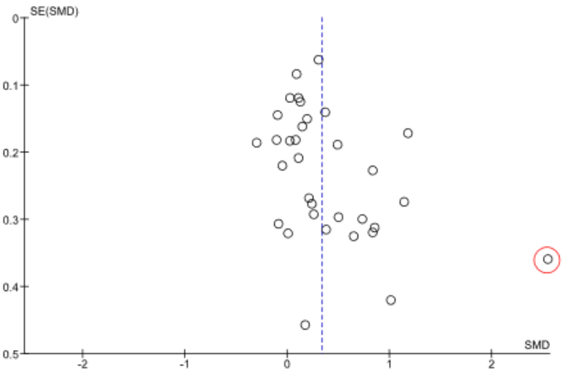


*No publication bias detected apart from outlier.*

*Outlier was visually identified and marked in red: Mansi et al., 2015.* [54]

**Studies reporting Energy Expenditure as outcome (N=5)**

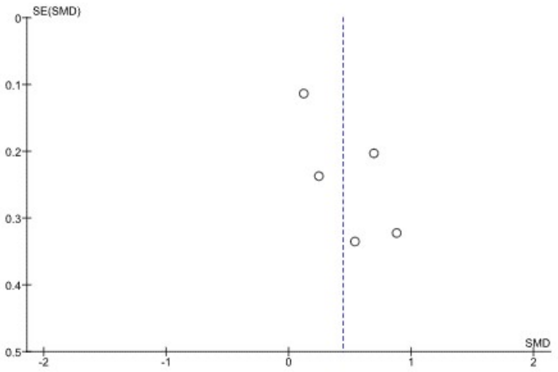


*No publication bias detected, small sample.*
