# Supplementary material for: Long-term Effectiveness of mHealth Physical Activity Interventions: Systematic Review and Meta-analysis of Randomized Controlled Trials
Source: J Med Internet Res. 2021 Apr 30;23(4):e26699. doi: 10.2196/26699 (PMC8122296; doi:10.2196/26699)
Supplement: Multimedia Appendix 6 [file jmir_v23i4e26699_app6.pdf]

Multimedia Appendix 6. Sensitivity analysis.

Scenarios	Outcome Measure	Timepoint	No. of studies	SMD 95% CI	P-value	*Heterogeneity*	
						I^2	P-value
**Baseline scenario**	Walking	End of intervention	77	0.46 [0.36, 0.55]	*P*<.001	83%	*P*<.001
		Short-term follow-up (≤6 months)	19	0.26 [0.09, 0.42]	*P*=.002	73%	*P*<.001
		Long-term follow-up (>6 months)	7	0.25 [0.10, 0.39]	*P*=.001	68%	*P*=.004
	MVPA	End of intervention	62	0.28 [0.21, 0.35]	*P*<.001	62%	*P*<.001
		Short-term follow-up (≤6 months)	21	0.20 [0.05, 0.35]	*P*=.008	72%	*P*<.001
		Long-term follow-up (>6 months)	7	0.19 [0.11, 0.27]	*P*<.001	0%	*P*=.44
	TPA	End of intervention	33	0.34 [0.20, 0.47]	*P*<.001	77%	*P*<.001
		Short-term follow-up (≤6 months)	9	0.53 [0.13, 0.93]	*P*=.009	87%	*P*<.001
		Long-term follow-up (>6 months)	6	0.19 [-0.00, 0.38]	*P*=.05	72%	*P*=.003
	EE	End of intervention	5	0.44 [0.13, 0.75]	*P*=.05	60%	*P*=.04
		Short-term follow-up (≤6 months)	1	0.52 [-0.10, 1.14]	*P*=.10	-	-
		Long-term follow-up (>6 months)	1	0.16 [-0.45, 0.77]	*P*=.61	-	-
**Without outlier studies**	Walking	End of intervention	74	0.39 [0.30, 0.48]	*P*<.001	78%	*P*<.001
		Short-term follow-up (≤6 months)	18	0.18 [0.06, 0.31]	*P*=.004	51%	*P*=.008
		Long-term follow-up (>6 months)	7	0.25 [0.10, 0.39]	*P*=.001	68%	*P*=.004
	MVPA	End of intervention	60	0.25 [0.19, 0.32]	*P*<.001	52%	*P*<.001
		Short-term follow-up (≤6 months)	20	0.12 [0.03, 0.22]	*P*=.008	28%	*P*=.12
		Long-term follow-up (>6 months)	7	0.19 [0.11, 0.27]	*P*<.001	0	*P*=.44
	TPA	End of intervention	32	0.28 [0.17, 0.40]	*P*<.001	69%	*P*<.001
		Short-term follow-up (≤6 months)	8	0.26 [0.03, 0.50]	*P*=.03	60%	*P*=.01
		Long-term follow-up (>6 months)	6	0.19 [-0.00, 0.38]	*P*=.05	72%	*P*=.003
	EE	End of intervention	5	0.44 [0.13, 0.75]	*P*=.005	60%	*P*=.04
		Short-term follow-up (≤6 months)	1	0.52 [-0.10, 1.14]	*P*=.10	-	-
		Long-term follow-up (>6 months)	1	0.16 [-0.45, 0.77]	*P*=.61	-	-
**Only low risk of bias studies**	Walking	End of intervention	17	0.59 [0.37, 0.82]	*P*<.001	84%	*P*<.001
		Short-term follow-up (≤6 months)	4	0.58 [-0.04, 1.21]	*P*=.07	87%	*P*<.001
		Long-term follow-up (>6 months)	2	0.38 [-0.33, 1.10]	*P*=.29	79%	*P*=.03
	MVPA	End of intervention	15	0.51 [0.29, 0.72]	*P*<.001	83%	*P*<.001
		Short-term follow-up (≤6 months)	5	0.56 [-0.13, 1.25]	*P*=.11	92%	*P*<.001
		Long-term follow-up (> 6 months)	2	0.12 [-0.04, 0.28]	*P*=.13	0%	*P*=.36
	TPA	End of intervention	5	0.66 [0.10, 1.23]	*P*=.02	91%	*P*<.001
		Short-term follow-up (≤6 months)	2	1.55 [-0.71, 3.81]	*P*=.18	97%	*P*<.001
		Long-term follow-up (>6 months)	2	0.09 [-0.07, 0.25]	*P*=.27	0%	*P*=.93
	EE	End of intervention	5	0.44 [0.13, 0.75]	*P*=.005	60%	*P*=.04
		Short-term follow-up (≤6 months)	1	0.52 [-0.10, 1.14]	*P*=.10	-	-
		Long-term follow-up (>6 months)	1	0.16 [-0.45, 0.77]	*P*=.61	-	-
**Only studies reporting long-term follow-ups**	Walking	End of intervention	7	0.39 [0.19, 0.59]	*P*<.001	83%	*P*<.001
		Short-term follow-up (≤6 months)	1	0.35 [0.03, 0.67]	*P*=.03	-	-
		Long-term follow-up (>6 months)	7	0.25 [0.10, 0.39]	*P*=.001	68%	*P*=.004
	MVPA	End of intervention	7	0.24 [0.08, 0.39]	*P*=.003	55%	*P*=.04
		Short-term follow-up (≤6 months)	2	0.29 [0.00, 0.57]	*P*=.05	0%	*P*=.50
		Long-term follow-up (>6 months)	7	0.19 [0.11, 0.27]	*P*<.001	0%	*P*=.44
	TPA	End of intervention	6	0.25 [0.09, 0.42]	*P*=.003	61%	*P*=.03
		Short-term follow-up (≤6 months)	1	0.26 [-0.06, 0.58]	*P*=.11	-	-
		Long-term follow-up (>6 months)	6	0.19 [-0.00, 0.38]	*P*=.05	72%	*P*=.003
	EE	End of intervention	1	0.88 [0.25, 1.51]	*P*=.006	-	-
		Short-term follow-up (≤6 months)	1	0.52 [-0.10, 1.14]	*P*=.10	-	-
		Long-term follow-up (>6 months)	1	0.16 [-0.45, 0.77]	*P*=.61	-	-
