# Supplementary material for: Long-term Effectiveness of mHealth Physical Activity Interventions: Systematic Review and Meta-analysis of Randomized Controlled Trials
Source: J Med Internet Res. 2021 Apr 30;23(4):e26699. doi: 10.2196/26699 (PMC8122296; doi:10.2196/26699)

Multimedia Appendix 8. Subgroup analysis by intervention design.

Subgroup analysis by intervention design for the outcome walking.

Study or Subgroup	Intervention Mean	Intervention SD	Intervention Total	Control Mean	Control SD	Control Total	Weight	Std. Mean Difference IV, Random, 95% CI
**4.1.1 Scalable (no human interaction)**								
Aittasalo, 2012 [41]	457	306	123	431	403	118	1.7%	0.07 [-0.18, 0.33]
Baker, 2008 [81]	9,977	4,669	39	7,078	2,911	40	1.3%	0.74 [0.28, 1.20]
Barwais, 2013 [82]	1,625	553.8	18	483	175.9	15	0.7%	2.61 [1.65, 3.57]
Butler, 2004 [83]	56,396	3,315	17	53,666	6,543	16	1.0%	0.52 [-0.18, 1.21]
Coelho, 2018 [43]	8,853	3,320	20	6,248	2,030	17	1.0%	0.91 [0.23, 1.59]
Compernolle, 2015 [85]	9,484	4,875	86	8,589	4,380	93	1.6%	0.19 [-0.10, 0.49]
Dadaczynski, 2017 [87]	526.5	240.6	80	442.9	200.4	64	1.5%	0.37 [0.04, 0.70]
Dishman, 2009 [91]	14.8	17.3	564	11.2	17.3	265	1.8%	0.21 [0.06, 0.35]
Finkelstein, 2016 [48]	-480	2,568	201	43.7	2,640.7	599	1.8%	-0.20 [-0.36, -0.04]
Gell, 2015 [95]	6,867.7	2,227	41	6,189	2,297	46	1.4%	0.30 [-0.13, 0.72]
Glynn, 2014 [97]	5,855	4,264	31	4,859	3,474	35	1.3%	0.25 [-0.23, 0.74]
Hultquist, 2005 [102]	10,159	292	27	8,270	354	31	0.5%	5.70 [4.51, 6.90]
Kernot, 2019 [52]	276.3	221.7	74	235	218.9	33	1.4%	0.19 [-0.23, 0.60]
Maher, 2015 [53]	332	289	51	160	185	59	1.4%	0.72 [0.33, 1.10]
Mansi, 2015 [54]	9,792	2,053	29	6,551	1,154	29	1.1%	1.92 [1.29, 2.55]
Martin, 2015 [111]	1,067	2,098	32	-1,042	2,202	16	1.1%	0.97 [0.34, 1.61]
Mendoza, 2015 [113]	3,080	3,254	52	138	1,950	50	1.4%	1.08 [0.67, 1.50]
Merom, 2007 [114]	52	107.2	105	48.9	126.8	209	1.7%	0.03 [-0.21, 0.26]
Nolan, 2017 [59]	272	2,275.9	63	155	2,557.1	59	1.5%	0.05 [-0.31, 0.40]
Paul, 2016 [115]	5,791	2,952	15	2,947	2,399	8	0.7%	0.99 [0.07, 1.90]
Poirier, 2016 [116]	5,411	2,277	107	4,751	1,834	110	1.6%	0.32 [0.05, 0.59]
Prestwich, 2010 [118]	1.98	1.9	88	1.2	1.6	46	1.5%	0.43 [0.07, 0.79]
Rowley, 2019 [121]	9,015	2,842	97	4,654	1,447	32	1.3%	1.69 [1.23, 2.14]
Simons, 2018 [64]	7,741	4,553	55	8,061	5,112	63	1.5%	-0.07 [-0.43, 0.30]
Spence, 2009 [122]	690	450.2	32	592.5	368.9	31	1.3%	0.23 [-0.26, 0.73]
Talbot, 2003 [67]	4,337	2,903	17	3,972	2,563	17	1.0%	0.13 [-0.54, 0.80]
Thorndike, 2014 [124]	7,886	3,622	50	7,600	3,492	49	1.4%	0.08 [-0.31, 0.47]
Vallance, 2016 [125]	5,923	3,109	41	6,885	3,576	37	1.3%	-0.29 [-0.73, 0.16]
Van Blarigan, 2019 [126]	10,047	4,461	20	12,541	5,535	19	1.0%	-0.49 [-1.13, 0.15]
Warren, 2014 [127]	8,371	3,069	37	7,576	2,993	69	1.4%	0.26 [-0.14, 0.66]
Yamada, 2012 [128]	3,726	1,607	40	2,267	1,837	42	1.3%	0.84 [0.38, 1.29]
**Subtotal (95% CI)**			**2252**			**2317**	**40.5%**	**0.54 [0.34, 0.74]**
Heterogeneity: Tau^2 = 0.26; Chi^2 = 266.67, df = 30 (P < 0.00001); I^2 = 89%								
Test for overall effect: Z = 5.27 (P < 0.00001)								
**4.1.2 Non-scalable (mHealth combined with human interactions)**								
Alsaleh, 2016 [79]	36.7	109.8	66	16.5	58.73	79	1.5%	0.23 [-0.09, 0.56]
Ashton, 2017 [80]	1,588.2	2,740.6	26	575.4	2,735.1	24	1.2%	0.36 [-0.20, 0.92]
Cadmus-Bertram, 2019 [84]	6,697	2,878	24	4,853	1,703	23	1.1%	0.76 [0.17, 1.36]
Creel, 2016 [86]	6,084	873	52	5,253	421	35	1.3%	1.13 [0.67, 1.60]
Croteau, 2004 [129]	2,419	6,074.6	7	2,320	47,312	8	0.6%	0.00 [-1.01, 1.02]
Croteau, 2007 [130]	6,180	3,530	79	6,378	2,994	68	1.5%	-0.06 [-0.38, 0.26]
Cruz, 2016 [44]	10,440	4,012	13	6,430	2,613	13	0.8%	1.15 [0.31, 1.99]
De Blok, 2006 [88]	3,927	2,617	8	3,554	2,019.6	8	0.7%	0.15 [-0.83, 1.13]
DeGreef, 2010 [73]	9,601	5,002	20	5,538	3,877	21	1.0%	0.89 [0.25, 1.54]
DeGreef, 2011[89]	7,703	2,729	60	3,883	2,537	32	1.3%	1.42 [0.94, 1.90]
DeGreef, 2011a [74]	6,771	3,889	43	5,173	3,094	24	1.3%	0.44 [-0.07, 0.94]
Demeyer, 2017 [90]	7	32.5	129	-10	23.5	132	1.7%	0.60 [0.35, 0.85]
Duru, 2010 [92]	9,883	14,014	34	2,426	8,429	28	1.2%	0.62 [0.11, 1.14]
Engel, 2006 [93]	150	117	24	216	87	30	1.2%	-0.64 [-1.19, -0.09]
Fjeldsoe, 2010 [94]	16.7	89.4	45	0.3	89.4	43	1.4%	0.18 [-0.24, 0.60]
Furber, 2010 [50]	249.9	196	97	202.6	189.5	107	1.6%	0.24 [-0.03, 0.52]
Gill, 2019 [96]	1,646	3,302	59	-1,485	3,171.5	59	1.5%	0.96 [0.58, 1.34]
Hardeman, 2020 [98]	8,419	3,224	417	8,191	3,003	442	1.8%	0.07 [-0.06, 0.21]
Hornikx, 2015 [99]	984	1,208	12	1,013	1,275	15	0.9%	-0.02 [-0.78, 0.74]
Hospes, 2009 [100]	7,872	3,962	18	6,172	3,194	17	1.0%	0.46 [-0.21, 1.13]
Houle, 2011 [101]	9,850	3,282	23	7,970	3,433	22	1.1%	0.55 [-0.05, 1.15]
Izawa, 2012 [103]	8,609.6	3,064.5	52	5,512.9	2,571.8	51	1.4%	1.09 [0.67, 1.50]
James, 2015 [51]	800.8	2,820.6	57	-1,294	3,304.4	52	1.4%	0.68 [0.29, 1.07]
Katzmarzyk, 2011[104]	7,248	2,737	20	6,637	2,418	23	1.1%	0.23 [-0.37, 0.83]
Kawagoshi, 2015 [105]	51.3	63.7	12	12.3	25.5	15	0.8%	0.82 [0.02, 1.61]
King, 2013 [106]	253.5	248.7	20	26.8	67	19	1.0%	1.21 [0.52, 1.89]
Kolt, 2012 [76]	107.4	164.9	130	92.2	120.5	123	1.7%	0.10 [-0.14, 0.35]
Li, 2020 [107]	6,673	3,462	55	5,819	2,860	57	1.5%	0.27 [-0.10, 0.64]
Long, 2013 [108]	65,983	18,069	38	47,596	13,900	33	1.3%	1.12 [0.61, 1.62]
Lynch, 2019 [109]	8,193	3,301	37	7,539	3,404	40	1.3%	0.19 [-0.26, 0.64]
Lyons, 2017 [110]	6,193	3,183	20	4,586	2,476	20	1.1%	0.55 [-0.08, 1.19]
Melville, 2015 [112]	4,823	2,059	42	4,784	2,613	40	1.4%	0.02 [-0.42, 0.45]
Mutrie, 2012 [58]	9,351	2,017	20	7,138	2,169	19	1.0%	1.04 [0.36, 1.71]
Oliveira, 2019 [60]	7,507	3,077	54	7,401	2,841	55	1.5%	0.04 [-0.34, 0.41]
Pope, 2018 [117]	5,175	2,308	12	4,746	2,045	8	0.7%	0.19 [-0.71, 1.08]
Reijonsaari, 2012 [119]	2,047	1,650	264	2,338	1,762	257	1.8%	-0.17 [-0.34, 0.00]
Ribeiro, 2014 [63]	969.7	1,464.1	101	-181.5	2,284	94	1.6%	0.60 [0.32, 0.89]
Roos, 2014 [120]	3,810	10,993.5	29	817.3	2,349.2	22	1.2%	0.35 [-0.21, 0.91]
Stacey, 2016 [65]	10,849	5,127	75	8,014	4,568	58	1.5%	0.58 [0.23, 0.93]
Tabak, 2014 [123]	5,603	3,475.8	13	4,617	3,460	16	0.9%	0.28 [-0.46, 1.01]
Ter Hoeve, 2018 [68]	8	2.6	121	7.7	3	252	1.7%	0.10 [-0.11, 0.32]
Tudor-Locke, 2004 [69]	9,123	4,539	24	5,622	2,405	23	1.1%	0.94 [0.34, 1.55]
Van Hoye, 2018 [72]	13.2	29.6	157	6.7	26	50	1.6%	0.23 [-0.09, 0.54]
Wyke, 2019 [77]	9,801	3,730	464	8,518	3,254	471	1.8%	0.37 [0.24, 0.50]
Yates, 2017 [78]	-486	2,252	287	-690	2,041	272	1.8%	0.09 [-0.07, 0.26]
**Subtotal (95% CI)**			**3360**			**3300**	**57.7%**	**0.42 [0.31, 0.54]**
Heterogeneity: Tau^2 = 0.10; Chi^2 = 188.72, df = 44 (P < 0.00001); I^2 = 77%								
Test for overall effect: Z = 7.19 (P < 0.00001)								
**4.1.3 Combined intervention**								
Harris, 2018 [75]	8,306	3,140	778	7,198	2,809	456	1.8%	0.37 [0.25, 0.48]
**Subtotal (95% CI)**			**778**			**456**	**1.8%**	**0.37 [0.25, 0.48]**
Heterogeneity: Not applicable								
Test for overall effect: Z = 6.16 (P < 0.00001)								
**Total (95% CI)**			**6390**			**6073**	**100.0%**	**0.45 [0.36, 0.55]**
Heterogeneity: Tau^2 = 0.14; Chi^2 = 458.02, df = 76 (P < 0.00001); I^2 = 83%								
Test for overall effect: Z = 8.98 (P < 0.00001)								
Test for subgroup differences: Chi^2 = 2.11, df = 2 (P = 0.35), I^2 = 5.2%								

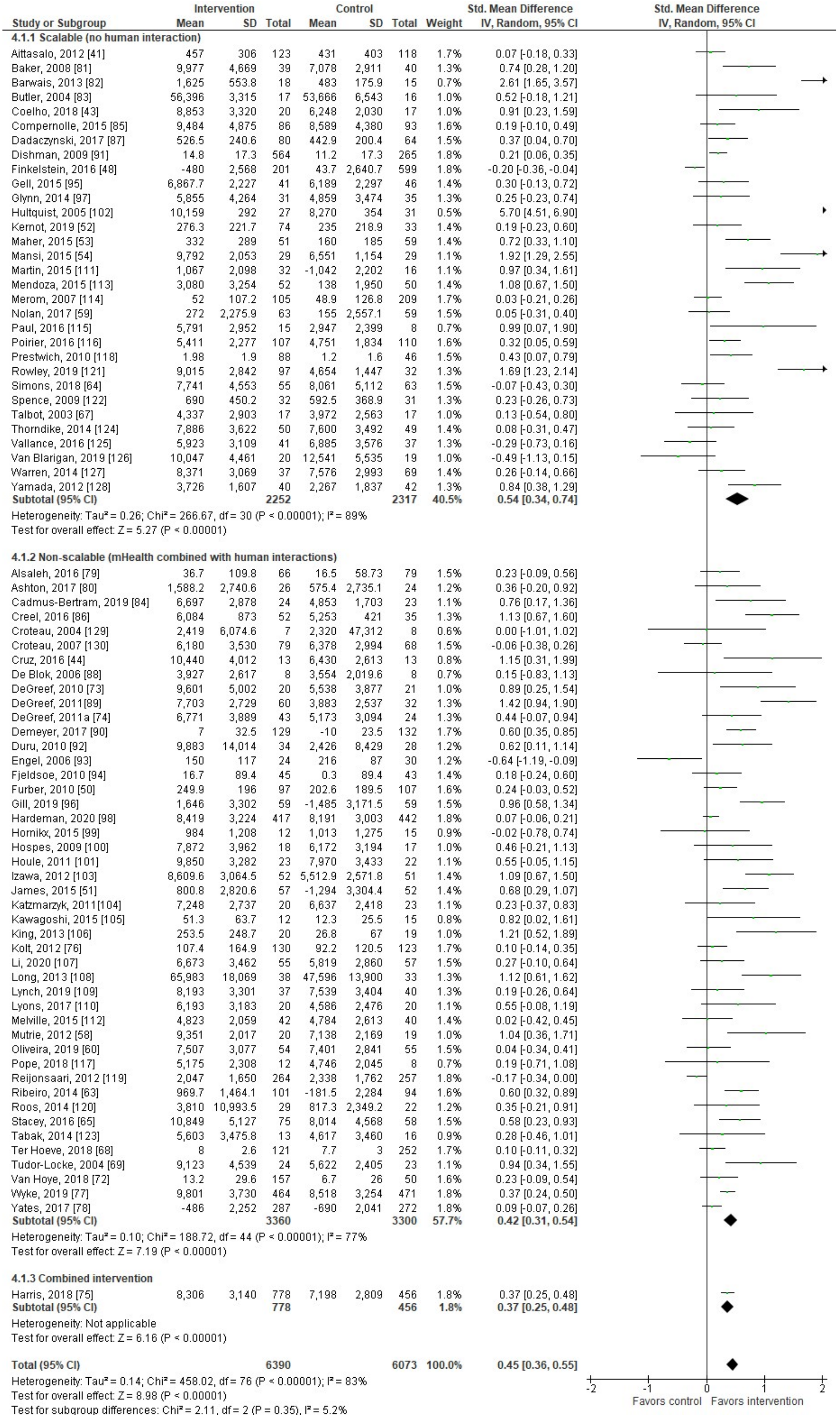

Subgroup analysis by intervention design for the outcome moderate-to-vigorous physical activity.

Study or Subgroup	Intervention Mean	Intervention SD	Intervention Total	Control Mean	Control SD	Control Total	Weight	Std. Mean Difference IV, Random, 95% CI
**4.2.1 Scalable (no human interaction)**								
Aittasalo, 2006 [131]	95	83.7	203	94	70.9	62	2.1%	0.01 [-0.27, 0.30]
Barwais, 2013 [82]	649	494.9	18	192	221.3	15	0.7%	1.13 [0.38, 1.87]
Compernolle, 2015 [85]	32.4	37.7	62	38.4	60	79	1.9%	-0.12 [-0.45, 0.22]
Dadaczynski, 2017 [87]	310.9	129.4	80	291.8	114	64	1.9%	0.15 [-0.17, 0.48]
Dishman, 2009 [91]	10.6	15.5	564	7.9	15.1	265	2.8%	0.18 [0.03, 0.32]
Edney, 2020 [47]	108.1	74.1	272	108.9	52.6	130	2.5%	-0.01 [-0.22, 0.20]
Golsteijn, 2018 [133]	331	234	208	301	219	211	2.6%	0.13 [-0.06, 0.32]
Kernot, 2019 [52]	204.1	131	74	150	96.9	33	1.5%	0.44 [0.03, 0.86]
King, 2008 [137]	310.6	298.3	19	135	208.2	18	0.8%	0.66 [0.00, 1.33]
Maher, 2015 [53]	528	391	51	391	371	59	1.7%	0.36 [-0.02, 0.74]
Mansi, 2015 [54]	1,469	524	29	538	254	29	0.8%	2.23 [1.57, 2.90]
Maselli, 2019 [55]	134.4	83.4	11	185.5	126.3	21	0.7%	-0.44 [-1.18, 0.30]
Merom, 2007 [114]	79	543.7	105	32.8	171.3	209	2.4%	0.13 [-0.10, 0.37]
Murawski, 2019 [57]	428.4	523.4	59	319.7	378.2	66	1.8%	0.24 [-0.11, 0.59]
Nolan, 2017 [59]	11	68.8	63	11	58.8	59	1.8%	0.00 [-0.36, 0.36]
Pekmezi, 2017 [141]	31.5	58.9	39	20.8	38.2	37	1.4%	0.21 [-0.24, 0.66]
Prestwich, 2009 [142]	1.3	1.2	60	0.97	1	94	1.9%	0.30 [-0.02, 0.63]
Sharp, 2016 [144]	100	109	72	90	119	65	1.9%	0.09 [-0.25, 0.42]
Simons, 2018 [64]	27.3	26.7	55	30.1	31	63	1.7%	-0.10 [-0.46, 0.27]
Vallance, 2008 [70]	213	173	172	180	141	166	2.5%	0.21 [-0.01, 0.42]
Vallance, 2016 [125]	143	132	41	146	155	37	1.4%	-0.02 [-0.47, 0.42]
Van Blarigan, 2019 [126]	46.6	48.4	20	54.5	24.9	19	0.9%	-0.20 [-0.83, 0.43]
Vandelanotte, 2018 [146]	148.8	181.1	78	79.8	77.1	46	1.7%	0.45 [0.08, 0.82]
**Subtotal (95% CI)**			**2355**			**1847**	**39.2%**	**0.20 [0.08, 0.32]**
Heterogeneity: Tau² = 0.05; Chi² = 66.16, df = 22 (P < 0.00001); I² = 67%								
Test for overall effect: Z = 3.30 (P = 0.0010)								
**4.2.2 Non-scalable (mHealth combined with human interactions)**								
Alsaleh, 2016 [79]	237.9	121.1	66	108.9	198	79	1.8%	0.77 [0.43, 1.11]
Ashton, 2017 [80]	154.1	216.1	26	26.1	210.2	24	1.0%	0.59 [0.02, 1.16]
Barnes, 2015 [42]	0.2	1.6	23	-0.2	1.5	19	0.9%	0.25 [-0.36, 0.86]
Cadmus-Bertram, 2019 [84]	230	120	24	143	107	23	1.0%	0.75 [0.16, 1.35]
Creel, 2016 [86]	37	41.6	52	20.4	17.1	35	1.4%	0.48 [0.05, 0.92]
Cruz, 2016 [44]	57.8	32.8	13	26.7	19.6	13	0.6%	1.11 [0.28, 1.95]
DeGreef, 2010 [73]	44	38	20	24	29	21	0.9%	0.58 [-0.04, 1.21]
DeGreef, 2011[89]	23	20	60	20	25	32	1.5%	0.14 [-0.29, 0.57]
DeGreef, 2011a [74]	82	84	43	27	39	24	1.2%	0.76 [0.24, 1.28]
Demeyer, 2017 [90]	8	21.1	140	-3	17.5	140	2.3%	0.57 [0.33, 0.80]
Eakin, 2014 [46]	163.6	155.6	119	114.6	108.6	127	2.3%	0.37 [0.11, 0.62]
Fischer, 2019 [132]	228.4	157.8	63	194	136	128	2.0%	0.24 [-0.06, 0.54]
Fjeldsoe, 2010 [94]	18.3	167.3	45	16.4	167.4	43	1.5%	0.01 [-0.41, 0.43]
Fjeldsoe, 2015 [49]	-8	262.4	104	-29.2	196	107	2.2%	0.09 [-0.18, 0.36]
Hardeman, 2020 [98]	77.3	36.5	417	76.7	35.4	442	2.8%	0.02 [-0.12, 0.15]
James, 2015 [51]	33.9	145.3	57	9.6	128.6	52	1.7%	0.18 [-0.20, 0.55]
Katzmarzyk, 2011[104]	16.3	17.3	20	16.2	17.1	23	1.0%	0.01 [-0.59, 0.60]
Kendzor, 2017 [134]	76	67	17	46	43	15	0.8%	0.51 [-0.19, 1.22]
Keyserling, 2008 [135]	14	3.7	86	13	2.8	89	2.0%	0.30 [0.01, 0.60]
Kim, 2018 [136]	51.2	4.4	41	50.3	4.4	42	1.5%	0.20 [-0.23, 0.63]
Koizumi, 2009 [138]	27.2	14.7	34	19	9.8	34	1.3%	0.65 [0.16, 1.14]
Kolt, 2012 [76]	121.2	184.4	130	111.4	110.6	123	2.3%	0.06 [-0.18, 0.31]
Li, 2017 [139]	64.2	70.5	17	56	60.1	17	0.8%	0.12 [-0.55, 0.80]
Li, 2020 [107]	44.7	41.2	55	31.6	32.4	57	1.7%	0.35 [-0.02, 0.73]
Lynch, 2019 [109]	249.6	159.7	40	170.1	127.9	40	1.4%	0.54 [0.10, 0.99]
Maxwell Smith, 2019 [140]	312	229.3	34	240	177.7	33	1.3%	0.35 [-0.14, 0.83]
Melville, 2015 [112]	3	2.6	42	3.1	2.1	40	1.4%	-0.04 [-0.47, 0.39]
Pinto, 2013 [61]	214	147.3	19	97	148	24	0.9%	0.78 [0.15, 1.40]
Pinto, 2015 [62]	70.3	65.9	36	16.5	31.9	32	1.2%	1.01 [0.50, 1.52]
Pope, 2018 [117]	34.2	18.7	12	37.8	20.4	8	0.5%	-0.18 [-1.07, 0.72]
Ribeiro, 2014 [63]	473.5	913.2	101	58.5	484.3	94	2.1%	0.56 [0.27, 0.85]
Samuels, 2011 [143]	34.8	13.6	12	26.5	16.1	25	0.8%	0.53 [-0.17, 1.23]
Ter Hoeve, 2018 [68]	6.7	3	121	6.5	3.1	252	2.4%	0.07 [-0.15, 0.28]
Unick, 2012 [145]	133	217	11	44.8	124.6	12	0.6%	0.49 [-0.35, 1.32]
Van der Weegen, 2015 [71]	48.2	23.8	65	42.9	25.8	134	2.0%	0.21 [-0.09, 0.51]
Van Hoye, 2018 [72]	17.8	49.6	157	9.6	34	50	1.9%	0.18 [-0.14, 0.49]
Wijsman, 2013 [147]	11.1	21.8	108	-0.1	15.4	105	2.1%	0.59 [0.32, 0.86]
Yates, 2017 [78]	-4.1	19	287	-5.9	17.7	272	2.7%	0.10 [-0.07, 0.26]
**Subtotal (95% CI)**			**2717**			**2830**	**57.9%**	**0.33 [0.24, 0.43]**
Heterogeneity: Tau² = 0.04; Chi² = 85.05, df = 37 (P < 0.0001); I² = 56%								
Test for overall effect: Z = 7.16 (P < 0.00001)								
**4.2.3 Combined intervention**								
Harris, 2018 [75]	374	179	778	310	168	456	2.9%	0.37 [0.25, 0.48]
**Subtotal (95% CI)**			**778**			**456**	**2.9%**	**0.37 [0.25, 0.48]**
Heterogeneity: Not applicable								
Test for overall effect: Z = 6.15 (P < 0.00001)								
**Total (95% CI)**			**5850**			**5133**	**100.0%**	**0.28 [0.21, 0.35]**
Heterogeneity: Tau² = 0.04; Chi² = 162.03, df = 61 (P < 0.00001); I² = 62%								
Test for overall effect: Z = 7.82 (P < 0.00001)								
Test for subgroup differences: Chi² = 4.23, df = 2 (P = 0.12), I² = 52.8%								

Std. Mean Difference
IV, Random, 95% CI

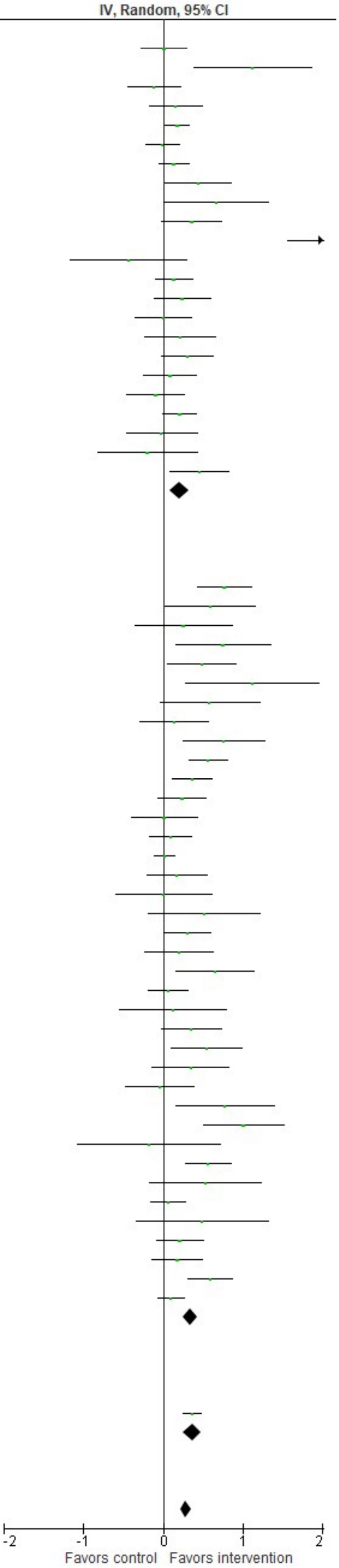

Subgroup analysis by intervention design for the outcome total physical activity.

Study or Subgroup	Intervention Mean	Intervention SD	Intervention Total	Control Mean	Control SD	Control Total	Weight	Std. Mean Difference IV, Random, 95% CI
**4.3.1 Scalable (no human interaction)**								
Aittasalo, 2006 [131]	509.5	479.4	203	555	519.7	62	3.8%	-0.09 [-0.38, 0.19]
Carr, 2013 [148]	171.4	234.5	25	121.8	174.6	28	2.6%	0.24 [-0.30, 0.78]
Compernolle, 2015 [85]	81.6	78	52	90.6	100.2	71	3.4%	-0.10 [-0.46, 0.26]
Kernot, 2019 [52]	298.9	97.7	74	288.8	68.6	33	3.2%	0.11 [-0.30, 0.52]
Mansi, 2015 [54]	1,035	444	29	188	135	29	2.0%	2.55 [1.84, 3.25]
Martin, 2015 [111]	10.5	21.1	32	-8	23	16	2.3%	0.84 [0.21, 1.46]
Merom, 2007 [114]	91	243.1	105	84.2	231.8	209	4.0%	0.03 [-0.21, 0.26]
Prestwich, 2010 [118]	3	1.79	88	2.3	14.3	46	3.5%	0.08 [-0.27, 0.44]
Simons, 2018 [64]	317	135	55	359.5	146	63	3.4%	-0.30 [-0.66, 0.06]
Suggs, 2013 [66]	49.5	39.6	79	5.8	33.9	79	3.5%	1.18 [0.84, 1.52]
Thorsteinsen, 2014 [153]	574	435	12	502	323	8	1.5%	0.17 [-0.72, 1.07]
Vandelanotte, 2018 [146]	387.7	377.7	78	230	164.1	46	3.4%	0.50 [0.13, 0.87]
**Subtotal (95% CI)**			**832**			**690**	**36.7%**	**0.39 [0.06, 0.73]**
Heterogeneity: Tau² = 0.30; Chi² = 97.59, df = 11 (P < 0.00001); I² = 89%								
Test for overall effect: Z = 2.28 (P = 0.02)								
**4.3.2 Non-scalable (mHealth combined with human interactions)**								
Cadmus-Bertram, 2019 [84]	1,463	489	24	1,343	395	23	2.5%	0.26 [-0.31, 0.84]
Cruz, 2016 [44]	279.5	74	13	212	53.9	13	1.7%	1.01 [0.19, 1.83]
DeGreef, 2010 [73]	301	106	20	260	104	21	2.3%	0.38 [-0.24, 1.00]
DeGreef, 2011[89]	93	66	60	40	56	32	3.0%	0.84 [0.39, 1.28]
DeGreef, 2011a [74]	176	108	43	65	68	24	2.6%	1.15 [0.61, 1.68]
Dlugonski, 2012 [45]	28.2	15.6	22	15.4	13.9	23	2.3%	0.85 [0.24, 1.47]
Furber, 2010 [50]	366.5	270.8	97	270.9	244.4	107	3.8%	0.37 [0.09, 0.65]
Gill, 2019 [96]	2.13	25.8	59	1.4	25.7	59	3.4%	0.03 [-0.33, 0.39]
Keyserling, 2008 [135]	29.8	6.5	86	28.6	5.7	90	3.7%	0.20 [-0.10, 0.49]
Kolt, 2012 [76]	168.9	190.5	130	146.3	163.5	123	4.0%	0.13 [-0.12, 0.37]
Lane, 2015 [149]	84.9	50.3	125	80.3	32.8	159	4.0%	0.11 [-0.12, 0.35]
Lyons, 2017 [110]	117	121	20	58	33	20	2.2%	0.65 [0.01, 1.29]
Mailey, 2010 [150]	299,792	102,800	24	251,625	83,081	23	2.5%	0.51 [-0.08, 1.09]
Melville, 2015 [112]	33.5	10	42	34	12	40	3.1%	-0.04 [-0.48, 0.39]
Motl, 2011 [151]	24.7	18.8	23	12.4	14.2	25	2.4%	0.73 [0.14, 1.32]
Müller, 2016 [56]	383.4	843.4	18	377.4	842.6	21	2.3%	0.01 [-0.62, 0.64]
Schwerdtfeger, 2012 [154]	738.6	245.7	21	690.5	217.1	41	2.7%	0.21 [-0.32, 0.74]
Sugden, 2008 [152]	108.7	54.7	26	113.822	62.337	18	2.4%	-0.09 [-0.69, 0.51]
Van Hoye, 2018 [72]	1.8	8.3	157	0.6	7	50	3.6%	0.15 [-0.17, 0.47]
Wyke, 2019 [77]	3,717	3,307	499	2,741	2,951	505	4.4%	0.31 [0.19, 0.44]
Yates, 2017 [78]	-32.3	94.6	287	-40.3	85	272	4.3%	0.09 [-0.08, 0.25]
**Subtotal (95% CI)**			**1796**			**1689**	**63.3%**	**0.30 [0.18, 0.42]**
Heterogeneity: Tau² = 0.03; Chi² = 42.43, df = 20 (P = 0.002); I² = 53%								
Test for overall effect: Z = 4.99 (P < 0.00001)								
**Total (95% CI)**			**2628**			**2379**	**100.0%**	**0.34 [0.20, 0.47]**
Heterogeneity: Tau² = 0.10; Chi² = 140.11, df = 32 (P < 0.00001); I² = 77%								
Test for overall effect: Z = 4.96 (P < 0.00001)								
Test for subgroup differences: Chi² = 0.27, df = 1 (P = 0.60), I² = 0%								

Std. Mean Difference
IV, Random, 95% CI

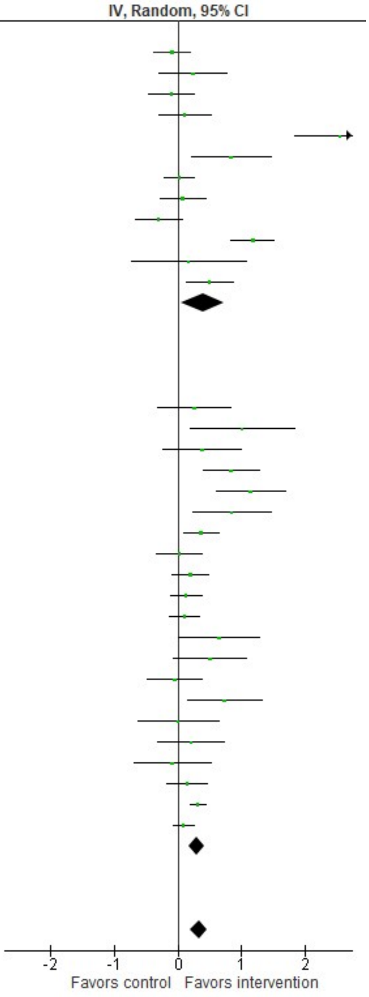

Supplement: Multimedia Appendix 8 [file jmir_v23i4e26699_app8.pdf]
