# Supplementary material for: Long-term Effectiveness of mHealth Physical Activity Interventions: Systematic Review and Meta-analysis of Randomized Controlled Trials
Source: J Med Internet Res. 2021 Apr 30;23(4):e26699. doi: 10.2196/26699 (PMC8122296; doi:10.2196/26699)

Multimedia Appendix 9. Subgroup analysis by control group type.

Subgroup analysis by control group type for the outcome walking.

Study or Subgroup	Intervention Mean	Intervention SD	Intervention Total	Control Mean	Control SD	Control Total	Weight	Std. Mean Difference IV, Random, 95% CI
**3.1.1 No/minimal intervention control group**								
Aittasalo, 2012 [41]	457	306	123	431	403	118	1.7%	0.07 [-0.18, 0.33]
Alsaleh, 2016 [79]	36.7	109.8	66	16.5	58.7	79	1.5%	0.23 [-0.09, 0.56]
Ashton, 2017 [80]	1,588.2	2,740.6	26	575.4	2,735.1	24	1.2%	0.36 [-0.20, 0.92]
Baker, 2008 [81]	9,977	4,669	39	7,078	2,911	40	1.3%	0.74 [0.28, 1.20]
Barwais, 2013 [82]	1,625	553.8	18	483	175.9	15	0.7%	2.61 [1.65, 3.57]
Butler, 2004 [83]	56,396	3,315	17	53,666	6,543	16	1.0%	0.52 [-0.18, 1.21]
Cadmus-Bertram, 2019 [84]	6,697	2,878	24	4,853	1,703	23	1.1%	0.76 [0.17, 1.36]
Coelho, 2018 [43]	8,853	3,320	20	6,248	2,030	17	1.0%	0.91 [0.23, 1.59]
Compernolle, 2015 [85]	9,484	4,875	86	8,589	4,380	93	1.6%	0.19 [-0.10, 0.49]
Creel, 2016 [86]	6,084	873	52	5,253	421	35	1.3%	1.13 [0.67, 1.60]
Croteau, 2004 [129]	2,419	6,074.6	7	2,320	4,732	8	0.6%	0.02 [-1.00, 1.03]
Croteau, 2007 [130]	6,180	3,530	79	6,378	2,994	68	1.5%	-0.06 [-0.38, 0.26]
Dadaczynski, 2017 [87]	526.5	240.6	80	442.9	200.4	64	1.5%	0.37 [0.04, 0.70]
De Blok, 2006 [88]	3,927	2,617	8	3,554	2,019.6	8	0.7%	0.15 [-0.83, 1.13]
DeGreef, 2010 [73]	9,601	5,002	20	5,538	3,877	21	1.0%	0.89 [0.25, 1.54]
DeGreef, 2011[89]	7,703	2,729	60	3,883	2,537	32	1.3%	1.42 [0.94, 1.90]
DeGreef, 2011a [74]	6,771	3,889	43	5,173	3,094	24	1.3%	0.44 [-0.07, 0.94]
Demeyer, 2017 [90]	7	32.5	129	-10	23.5	132	1.7%	0.60 [0.35, 0.85]
Dishman, 2009 [91]	14.8	17.3	564	11.2	17.3	265	1.8%	0.21 [0.06, 0.35]
Finkelstein, 2016 [48]	-480	2,568	201	43.7	2,640.7	599	1.8%	-0.20 [-0.36, -0.04]
Fjeldsoe, 2010 [94]	16.7	89.4	45	0.3	89.4	43	1.4%	0.18 [-0.24, 0.60]
Furber, 2010 [50]	249.9	196	97	202.6	189.5	107	1.6%	0.24 [-0.03, 0.52]
Gill, 2019 [96]	1,646	3,302	59	-1,485	3,171.5	59	1.5%	0.96 [0.58, 1.34]
Glynn, 2014 [97]	5,855	4,264	31	4,859	3,474	35	1.3%	0.25 [-0.23, 0.74]
Hardeman, 2020 [98]	8,419	3,224	417	8,191	3,003	442	1.8%	0.07 [-0.06, 0.21]
Harris, 2018 [75]	8,306	3,140	778	7,198	2,809	456	1.8%	0.37 [0.25, 0.48]
Hornikx, 2015 [99]	984	1,208	12	1,013	1,275	15	0.9%	-0.02 [-0.78, 0.74]
Hospes, 2009 [100]	7,872	3,962	18	6,172	3,194	17	1.0%	0.46 [-0.21, 1.13]
Houle, 2011 [101]	9,850	3,282	23	7,970	3,433	22	1.1%	0.55 [-0.05, 1.15]
Hultquist, 2005 [102]	10,159	292	27	8,270	354	31	0.5%	5.70 [4.51, 6.90]
James, 2015 [51]	800.8	2,820.6	57	-1,294	3,304.4	52	1.4%	0.68 [0.29, 1.07]
Katzmarzyk, 2011[104]	7,248	2,737	20	6,637	2,418	23	1.1%	0.23 [-0.37, 0.83]
Kernot, 2019 [52]	276.3	221.7	74	235	218.9	33	1.4%	0.19 [-0.23, 0.60]
King, 2013 [106]	253.5	248.7	20	26.8	67	19	1.0%	1.21 [0.52, 1.89]
Li, 2020 [107]	6,673	3,462	55	5,819	2,860	57	1.5%	0.27 [-0.10, 0.64]
Long, 2013 [108]	65,983	18,069	38	47,596	13,900	33	1.3%	1.12 [0.61, 1.62]
Lynch, 2019 [109]	8,193	3,301	37	7,539	3,404	40	1.3%	0.19 [-0.26, 0.64]
Lyons, 2017 [110]	6,193	3,183	20	4,586	2,476	20	1.1%	0.55 [-0.08, 1.19]
Maher, 2015 [53]	332	289	51	160	185	59	1.4%	0.72 [0.33, 1.10]
Mansi, 2015 [54]	9,792	2,053	29	6,551	1,154	29	1.1%	1.92 [1.29, 2.55]
Martin, 2015 [111]	1,067	2,098	32	-1,042	2,202	16	1.1%	0.97 [0.34, 1.61]
Melville, 2015 [112]	4,823	2,059	42	4,784	2,613	40	1.4%	0.02 [-0.42, 0.45]
Mutrie, 2012 [58]	9,351	2,017	20	7,138	2,169	19	1.0%	1.04 [0.36, 1.71]
Nolan, 2017 [59]	272	2,275.9	63	155	2,557.1	59	1.5%	0.05 [-0.31, 0.40]
Oliveira, 2019 [60]	7,507	3,077	54	7,401	2,841	55	1.5%	0.04 [-0.34, 0.41]
Paul, 2016 [115]	5,791	2,952	15	2,947	2,399	8	0.7%	0.99 [0.07, 1.90]
Poirier, 2016 [116]	5,411	2,277	107	4,751	1,834	110	1.6%	0.32 [0.05, 0.59]
Prestwich, 2010 [118]	1.98	1.9	88	1.17	1.58	46	1.5%	0.45 [0.09, 0.81]
Reijonsaari, 2012 [119]	2,047	1,650	264	2,338	1,762	257	1.8%	-0.17 [-0.34, 0.00]
Roos, 2014 [120]	3,810	10,993.5	29	817.3	2,349.2	22	1.2%	0.35 [-0.21, 0.91]
Rowley, 2019 [121]	9,015	2,842	97	4,654	1,447	32	1.3%	1.69 [1.23, 2.14]
Simons, 2018 [64]	7,741	4,553	55	8,061	5,112	63	1.5%	-0.07 [-0.43, 0.30]
Stacey, 2016 [65]	10,849	5,127	75	8,014	4,568	58	1.5%	0.58 [0.23, 0.93]
Tabak, 2014 [123]	5,603	3,475.8	13	4,617	3,460	16	0.9%	0.28 [-0.46, 1.01]
Thorndike, 2014 [124]	7,886	3,622	50	7,600	3,492	49	1.4%	0.08 [-0.31, 0.47]
Tudor-Locke, 2004 [69]	9,123	4,539	24	5,622	2,405	23	1.1%	0.94 [0.34, 1.55]
Vallance, 2016 [125]	5,923	3,109	41	6,885	3,576	37	1.3%	-0.29 [-0.73, 0.16]
Van Blarigan, 2019 [126]	10,047	4,461	20	12,541	5,535	19	1.0%	-0.49 [-1.13, 0.15]
Van Hoye, 2018 [72]	13.2	29.6	157	6.7	26	50	1.6%	0.23 [-0.09, 0.54]
Wyke, 2019 [77]	9,801	3,730	464	8,518	3,254	471	1.8%	0.37 [0.24, 0.50]
Yamada, 2012 [128]	3,726	1,607	40	2,267	1,837	42	1.3%	0.84 [0.38, 1.29]
Yates, 2017 [78]	-486	2,252	287	-690	2,041	272	1.8%	0.09 [-0.07, 0.26]
**Subtotal (95% CI)**			**5607**			**5037**	**80.7%**	**0.47 [0.36, 0.59]**
Heterogeneity: Tau² = 0.14; Chi² = 397.03, df = 61 (P < 0.00001); I² = 85%								
Test for overall effect: Z = 8.29 (P < 0.00001)								
**3.1.2 Alternative intervention control group**								
Cruz, 2016 [44]	10,440	4,012	13	6,430	2,613	13	0.8%	1.15 [0.31, 1.99]
Duru, 2010 [92]	9,883	14,014	34	2,426	8,429	28	1.2%	0.62 [0.11, 1.14]
Engel, 2006 [93]	150	117	24	216	87	30	1.2%	-0.64 [-1.19, -0.09]
Gell, 2015 [95]	6,867.7	2,227	41	6,189	2,297	46	1.4%	0.30 [-0.13, 0.72]
Izawa, 2012 [103]	8,609.6	3,064.5	52	5,512.9	2,571.8	51	1.4%	1.09 [0.67, 1.50]
Kawagoshi, 2015 [105]	51.3	63.7	12	12.3	25.5	15	0.8%	0.82 [0.02, 1.61]
Kolt, 2012 [76]	107.4	164.9	130	92.2	120.5	123	1.7%	0.10 [-0.14, 0.35]
Mendoza, 2015 [113]	3,080	3,254	52	138	1,950	50	1.4%	1.08 [0.67, 1.50]
Pope, 2018 [117]	5,175	2,308	12	4,746	2,045	8	0.7%	0.19 [-0.71, 1.08]
Talbot, 2003 [67]	4,337	2,903	17	3,972	2,563	17	1.0%	0.13 [-0.54, 0.80]
**Subtotal (95% CI)**			**387**			**381**	**11.6%**	**0.48 [0.12, 0.83]**
Heterogeneity: Tau² = 0.25; Chi² = 45.88, df = 9 (P < 0.00001); I² = 80%								
Test for overall effect: Z = 2.60 (P = 0.009)								
**3.1.3 Combined control group**								
Merom, 2007 [114]	52	107.17	105	48.9	126.83	209	1.7%	0.03 [-0.21, 0.26]
Ribeiro, 2014 [63]	969.7	1,464.1	101	-181.5	2,284	94	1.6%	0.60 [0.32, 0.89]
Spence, 2009 [122]	690	450.2	32	592.5	368.9	31	1.3%	0.23 [-0.26, 0.73]
Ter Hoeve, 2018 [68]	8	2.6	121	7.7	3	252	1.7%	0.10 [-0.11, 0.32]
Warren, 2014 [127]	8,371	3,069	37	7,576	2,993	69	1.4%	0.26 [-0.14, 0.66]
**Subtotal (95% CI)**			**396**			**655**	**7.7%**	**0.23 [0.01, 0.45]**
Heterogeneity: Tau² = 0.04; Chi² = 10.51, df = 4 (P = 0.03); I² = 62%								
Test for overall effect: Z = 2.08 (P = 0.04)								
**Total (95% CI)**			**6390**			**6073**	**100.0%**	**0.46 [0.36, 0.55]**
Heterogeneity: Tau² = 0.14; Chi² = 458.16, df = 76 (P < 0.00001); I² = 83%								
Test for overall effect: Z = 8.98 (P < 0.00001)								
Test for subgroup differences: Chi² = 3.75, df = 2 (P = 0.15), I² = 46.7%								

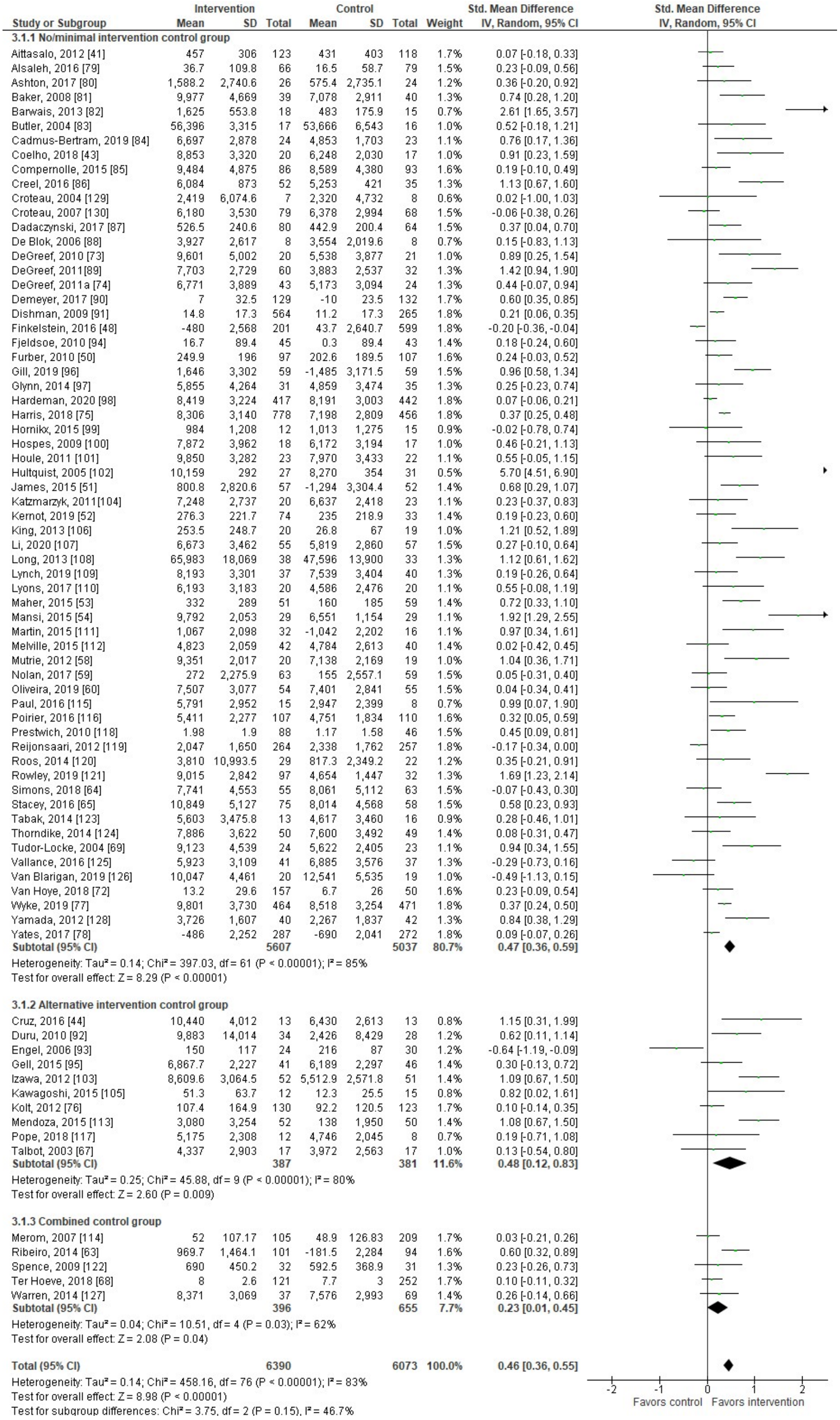

Subgroup analysis by control group type for the outcome moderate-to-vigorous physical activity.

	Intervention			Control				Std. Mean Difference
**Study or Subgroup**	**Mean**	**SD**	**Total**	**Mean**	**SD**	**Total**	**Weight**	**IV, Random, 95% CI**
**3.2.1 No/minimal intervention control group**								
Alsaleh, 2016 [79]	237.9	121.1	66	108.9	198	79	1.8%	0.77 [0.43, 1.11]
Ashton, 2017 [80]	154.1	216.1	26	26.1	210.2	24	1.0%	0.59 [0.02, 1.16]
Barnes, 2015 [42]	0.2	1.6	23	-0.2	1.5	19	0.9%	0.25 [-0.36, 0.86]
Barwais, 2013 [82]	649	494.9	18	192	221.3	15	0.7%	1.13 [0.38, 1.87]
Cadmus-Bertram, 2019 [84]	230	120	24	143	107	23	1.0%	0.75 [0.16, 1.35]
Compernolle, 2015 [85]	32.4	37.7	62	38.4	60	79	1.9%	-0.12 [-0.45, 0.22]
Creel, 2016 [86]	37	41.6	52	20.4	17.1	35	1.4%	0.48 [0.05, 0.92]
Dadaczynski, 2017 [87]	310.9	129.4	80	291.8	114	64	1.9%	0.15 [-0.17, 0.48]
DeGreef, 2010 [73]	44	38	20	24	29	21	0.9%	0.58 [-0.04, 1.21]
DeGreef, 2011[89]	23	20	60	20	25	32	1.5%	0.14 [-0.29, 0.57]
DeGreef, 2011a [74]	82	84	43	27	39	24	1.2%	0.76 [0.24, 1.28]
Demeyer, 2017 [90]	8	21.1	140	-3	17.5	140	2.3%	0.57 [0.33, 0.80]
Dishman, 2009 [91]	10.6	15.5	564	7.9	15.1	265	2.8%	0.18 [0.03, 0.32]
Eakin, 2014 [46]	163.6	155.6	119	114.6	108.6	127	2.3%	0.37 [0.11, 0.62]
Edney, 2020 [47]	108.1	74.1	272	108.9	52.6	130	2.5%	-0.01 [-0.22, 0.20]
Fjeldsoe, 2010 [94]	18.3	167.3	45	16.4	167.4	43	1.5%	0.01 [-0.41, 0.43]
Golsteijn, 2018 [133]	331	234	208	301	219	211	2.6%	0.13 [-0.06, 0.32]
Hardeman, 2020 [98]	77.3	36.5	417	76.7	35.4	442	2.8%	0.02 [-0.12, 0.15]
Harris, 2018 [75]	374	179	778	310	168	456	2.9%	0.37 [0.25, 0.48]
James, 2015 [51]	33.9	145.3	57	9.6	128.6	52	1.7%	0.18 [-0.20, 0.55]
Katzmarzyk, 2011[104]	16.3	17.3	20	16.2	17.1	23	1.0%	0.01 [-0.59, 0.60]
Kendzor, 2017 [134]	76	67	17	46	43	15	0.8%	0.51 [-0.19, 1.22]
Kernot, 2019 [52]	204.1	131	74	150	96.9	33	1.5%	0.44 [0.03, 0.86]
Keyserling, 2008 [135]	14	3.71	86	13	2.8	89	2.0%	0.30 [0.01, 0.60]
King, 2008 [137]	310.6	298.3	19	135	208.2	18	0.8%	0.66 [0.00, 1.33]
Koizumi, 2009 [138]	27.2	14.7	34	19	9.8	34	1.3%	0.65 [0.16, 1.14]
Li, 2017 [139]	64.2	70.5	17	56	60.1	17	0.8%	0.12 [-0.55, 0.80]
Li, 2020 [107]	44.7	41.2	55	31.6	32.4	57	1.7%	0.35 [-0.02, 0.73]
Lynch, 2019 [109]	249.6	159.7	40	170.1	127.9	40	1.4%	0.54 [0.10, 0.99]
Maher, 2015 [53]	528	391	51	391	371	59	1.7%	0.36 [-0.02, 0.74]
Mansi, 2015 [54]	1,469	524	29	538	254	29	0.8%	2.23 [1.57, 2.90]
Maxwell Smith, 2019 [140]	312	229.3	34	240	177.7	33	1.3%	0.35 [-0.14, 0.83]
Melville, 2015 [112]	3	2.6	42	3.1	2.1	40	1.4%	-0.04 [-0.47, 0.39]
Murawski, 2019 [57]	428.4	523.4	59	319.7	378.2	66	1.8%	0.24 [-0.11, 0.59]
Nolan, 2017 [59]	11	68.8	63	11	58.8	59	1.8%	0.00 [-0.36, 0.36]
Pekmezi, 2017 [141]	31.5	58.9	39	20.8	38.2	37	1.4%	0.21 [-0.24, 0.66]
Sharp, 2016 [144]	100	109	72	90	119	65	1.9%	0.09 [-0.25, 0.42]
Simons, 2018 [64]	27.3	26.7	55	30.1	31	63	1.7%	-0.10 [-0.46, 0.27]
Vallance, 2016 [125]	143	132	41	146	155	37	1.4%	-0.02 [-0.47, 0.42]
Van Blarigan, 2019 [126]	46.6	48.4	20	54.5	24.9	19	0.9%	-0.20 [-0.83, 0.43]
Van Hoye, 2018 [72]	17.8	49.6	157	9.6	34	50	1.9%	0.18 [-0.14, 0.49]
Wijsman, 2013 [147]	11.1	21.8	108	-0.1	15.4	105	2.1%	0.59 [0.32, 0.86]
Yates, 2017 [78]	-4.1	19	287	-5.9	17.7	272	2.7%	0.10 [-0.07, 0.26]
**Subtotal (95% CI)**			**4493**			**3541**	**69.7%**	**0.29 [0.21, 0.38]**
Heterogeneity: Tau² = 0.05; Chi² = 125.93, df = 42 (P < 0.00001); I² = 67%								
Test for overall effect: Z = 6.52 (P < 0.00001)								
**3.2.2 Alternative intervention control group**								
Cruz, 2016 [44]	57.8	32.8	13	26.7	19.6	13	0.6%	1.11 [0.28, 1.95]
Fjeldsoe, 2015 [49]	-8	262.4	104	-29.2	196	107	2.2%	0.09 [-0.18, 0.36]
Kim, 2018 [136]	51.2	4.43	41	50.25	4.4	42	1.5%	0.21 [-0.22, 0.64]
Kolt, 2012 [76]	121.2	184.4	130	111.4	110.6	123	2.3%	0.06 [-0.18, 0.31]
Pinto, 2013 [61]	214	147.3	19	97	148	24	0.9%	0.78 [0.15, 1.40]
Pinto, 2015 [62]	70.3	65.9	36	16.5	31.9	32	1.2%	1.01 [0.50, 1.52]
Pope, 2018 [117]	34.2	18.7	12	37.8	20.4	8	0.5%	-0.18 [-1.07, 0.72]
Unick, 2012 [145]	133	217	11	44.8	124.6	12	0.6%	0.49 [-0.35, 1.32]
Vandelanotte, 2018 [146]	148.8	181.1	78	79.8	77.1	46	1.7%	0.45 [0.08, 0.82]
**Subtotal (95% CI)**			**444**			**407**	**11.4%**	**0.39 [0.14, 0.65]**
Heterogeneity: Tau² = 0.08; Chi² = 21.18, df = 8 (P = 0.007); I² = 62%								
Test for overall effect: Z = 3.06 (P = 0.002)								
**3.2.3 Combined control group**								
Aittasalo, 2006 [131]	95	83.7	203	94	70.9	62	2.1%	0.01 [-0.27, 0.30]
Fischer, 2019 [132]	228.4	157.8	63	194	136	128	2.0%	0.24 [-0.06, 0.54]
Maselli, 2019 [55]	134.4	83.4	11	185.5	126.3	21	0.7%	-0.44 [-1.18, 0.30]
Merom, 2007 [114]	79	543.7	105	32.8	171.3	209	2.4%	0.13 [-0.10, 0.37]
Prestwich, 2009 [142]	1.3	1.2	60	0.97	1	94	1.9%	0.30 [-0.02, 0.63]
Ribeiro, 2014 [63]	473.5	913.2	101	58.5	484.3	94	2.1%	0.56 [0.27, 0.85]
Samuels, 2011 [143]	34.8	13.6	12	26.5	16.1	25	0.8%	0.53 [-0.17, 1.23]
Ter Hoeve, 2018 [68]	6.7	3	121	6.5	3.1	252	2.4%	0.07 [-0.15, 0.28]
Vallance, 2008 [70]	213	173	172	180	141	166	2.5%	0.21 [-0.01, 0.42]
Van der Weegen, 2015 [71]	48.2	23.8	65	42.9	25.8	134	2.0%	0.21 [-0.09, 0.51]
**Subtotal (95% CI)**			**913**			**1185**	**18.9%**	**0.20 [0.08, 0.32]**
Heterogeneity: Tau² = 0.01; Chi² = 13.68, df = 9 (P = 0.13); I² = 34%								
Test for overall effect: Z = 3.33 (P = 0.0009)								
**Total (95% CI)**			**5850**			**5133**	**100.0%**	**0.28 [0.21, 0.35]**
Heterogeneity: Tau² = 0.04; Chi² = 162.02, df = 61 (P < 0.00001); I² = 62%								
Test for overall effect: Z = 7.83 (P < 0.00001)								
Test for subgroup differences: Chi² = 2.66, df = 2 (P = 0.26), I² = 24.7%								

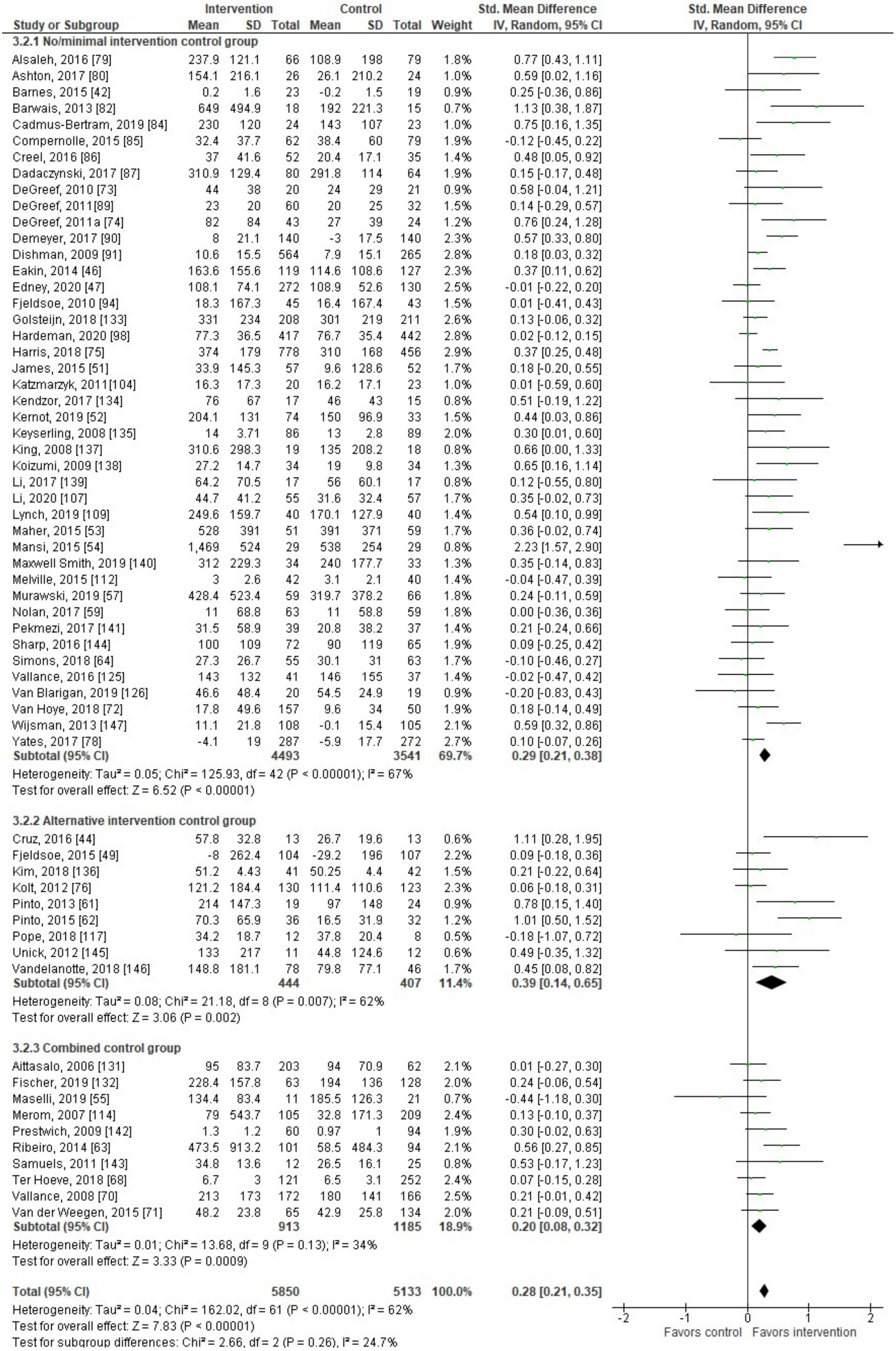

Subgroup analysis by control group type for the outcome total physical activity.

Study or Subgroup	Intervention Mean	Intervention SD	Intervention Total	Control Mean	Control SD	Control Total	Weight	Std. Mean Difference IV, Random, 95% CI
**3.3.1 No/minimal intervention control group**								
Cadmus-Bertram, 2019 [84]	1,463	489	24	1,343	395	23	2.5%	0.26 [-0.31, 0.84]
Compernolle, 2015 [85]	81.6	78	52	90.6	100.2	71	3.4%	-0.10 [-0.46, 0.26]
DeGreef, 2010 [73]	301	106	20	260	104	21	2.3%	0.38 [-0.24, 1.00]
DeGreef, 2011[89]	93	66	60	40	56	32	3.0%	0.84 [0.39, 1.28]
DeGreef, 2011a [74]	176	108	43	65	68	24	2.6%	1.15 [0.61, 1.68]
Dlugonski, 2012 [45]	28.2	15.6	22	15.4	13.9	23	2.3%	0.85 [0.24, 1.47]
Furber, 2010 [50]	366.5	270.8	97	270.9	244.4	107	3.8%	0.37 [0.09, 0.65]
Gill, 2019 [96]	2.1	25.8	59	1.37	25.7	59	3.4%	0.03 [-0.33, 0.39]
Kernot, 2019 [52]	298.9	97.7	74	288.8	68.6	33	3.2%	0.11 [-0.30, 0.52]
Keyserling, 2008 [135]	29.8	6.5	86	28.6	5.7	90	3.7%	0.20 [-0.10, 0.49]
Lane, 2015 [149]	84.9	50.3	125	80.3	32.8	159	4.0%	0.11 [-0.12, 0.35]
Lyons, 2017 [110]	117	121	20	58	33	20	2.2%	0.65 [0.01, 1.29]
Mailey, 2010 [150]	299,792	102,800	24	251,625	83,081	23	2.5%	0.51 [-0.08, 1.09]
Mansi, 2015 [54]	1,035	444	29	188	135	29	2.0%	2.55 [1.84, 3.25]
Martin, 2015 [111]	10.5	21.1	32	-8	23	16	2.3%	0.84 [0.21, 1.46]
Melville, 2015 [112]	33.5	10	42	34	12	40	3.1%	-0.04 [-0.48, 0.39]
Motl, 2011 [151]	24.7	18.8	23	12.4	14.2	25	2.4%	0.73 [0.14, 1.32]
Müller, 2016 [56]	383.4	843.4	18	377.4	842.6	21	2.3%	0.01 [-0.62, 0.64]
Prestwich, 2010 [118]	2.96	1.8	88	2.3	14.3	46	3.5%	0.08 [-0.28, 0.43]
Simons, 2018 [64]	317	135	55	359.5	146	63	3.4%	-0.30 [-0.66, 0.06]
Thorsteinsen, 2014 [153]	574	435	12	502	323	8	1.5%	0.17 [-0.72, 1.07]
Van Hoye, 2018 [72]	1.8	8.3	157	0.6	7	50	3.6%	0.15 [-0.17, 0.47]
Wyke, 2019 [77]	3,717	3,307	499	2,741	2,951	505	4.4%	0.31 [0.19, 0.44]
Yates, 2017 [78]	-32.3	94.6	287	-40.3	85	272	4.3%	0.09 [-0.08, 0.25]
**Subtotal (95% CI)**			**1948**			**1760**	**72.0%**	**0.34 [0.19, 0.50]**
Heterogeneity: Tau² = 0.09; Chi² = 95.04, df = 23 (P < 0.00001); I² = 76%								
Test for overall effect: Z = 4.40 (P < 0.0001)								
**3.3.2 Alternative intervention control group**								
Carr, 2013 [148]	171.4	234.5	25	121.8	174.6	28	2.6%	0.24 [-0.30, 0.78]
Cruz, 2016 [44]	279.5	74	13	212	53.9	13	1.7%	1.01 [0.19, 1.83]
Kolt, 2012 [76]	168.9	190.5	130	146.3	163.5	123	4.0%	0.13 [-0.12, 0.37]
Sugden, 2008 [152]	108.7	54.7	26	113.8	62.3	18	2.4%	-0.09 [-0.69, 0.51]
Suggs, 2013 [66]	49.5	39.6	79	5.8	33.9	79	3.5%	1.18 [0.84, 1.52]
Vandelanotte, 2018 [146]	387.7	377.7	78	230	164.1	46	3.4%	0.50 [0.13, 0.87]
**Subtotal (95% CI)**			**351**			**307**	**17.6%**	**0.48 [0.06, 0.91]**
Heterogeneity: Tau² = 0.22; Chi² = 29.92, df = 5 (P < 0.0001); I² = 83%								
Test for overall effect: Z = 2.23 (P = 0.03)								
**3.3.3 Combined control group**								
Aittasalo, 2006 [131]	509.5	479.4	203	555	519.7	62	3.8%	-0.09 [-0.38, 0.19]
Merom, 2007 [114]	91	243.1	105	84.2	231.8	209	4.0%	0.03 [-0.21, 0.26]
Schwerdtfeger, 2012 [154]	738.6	245.7	21	690.5	217.1	41	2.7%	0.21 [-0.32, 0.74]
**Subtotal (95% CI)**			**329**			**312**	**10.5%**	**0.00 [-0.17, 0.17]**
Heterogeneity: Tau² = 0.00; Chi² = 1.07, df = 2 (P = 0.59); I² = 0%								
Test for overall effect: Z = 0.04 (P = 0.97)								
**Total (95% CI)**			**2628**			**2379**	**100.0%**	**0.34 [0.20, 0.47]**
Heterogeneity: Tau² = 0.10; Chi² = 140.16, df = 32 (P < 0.00001); I² = 77%								
Test for overall effect: Z = 4.96 (P < 0.00001)								
Test for subgroup differences: Chi² = 10.12, df = 2 (P = 0.006), I² = 80.2%								

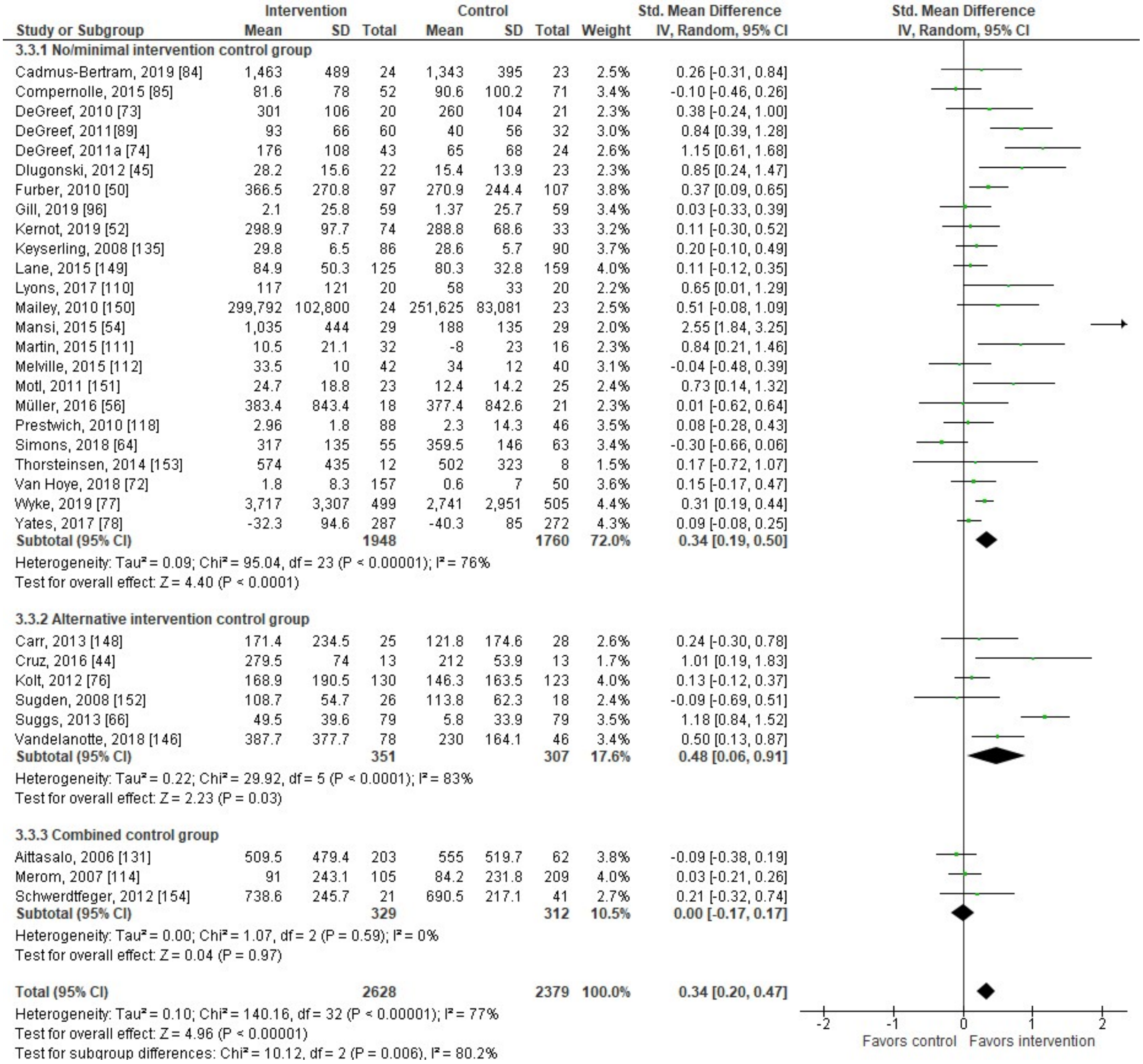

Supplement: Multimedia Appendix 9 [file jmir_v23i4e26699_app9.pdf]
