# Supplementary material for: Long-term Effectiveness of mHealth Physical Activity Interventions: Systematic Review and Meta-analysis of Randomized Controlled Trials
Source: J Med Internet Res. 2021 Apr 30;23(4):e26699. doi: 10.2196/26699 (PMC8122296; doi:10.2196/26699)
Supplement: Multimedia Appendix 10 [file jmir_v23i4e26699_app10.pdf]

Multimedia Appendix 10. Study-specific risk of bias judgments.

	Random sequence generation (selection bias)	Allocation concealment (selection bias)	Blinding of participants and personnel (performance bias)	Blinding of outcome assessment (detection bias)	Incomplete outcome data (attrition bias)	Selective outcome reporting	Other	Summary
Aittasalo 2006 [131]	🔴	🟡	🟡	🔴	🔴	🟡	🟢	High Risk
Aittasalo 2012 [41]	🟢	🟡	🟡	🔴	🟡	🟢	🟢	High Risk
Alsaleh 2016 [79]	🟢	🟢	🔴	🔴	🟢	🟢	🟢	High Risk
Ashton 2017 [80]	🟢	🟢	🟡	🟢	🟢	🟢	🟢	Low Risk
Baker 2008 [81]	🟢	🟢	🔴	🟢	🔴	🟡	🟢	High Risk
Barnes 2015 [42]	🟢	🟢	🟡	🟡	🟡	🟢	🟢	Low Risk
Barwais 2013 [82]	🟢	🟡	🔴	🟡	🟢	🟡	🟢	Low Risk
Bennett 2008 [155]	🟢	🟡	🔴	🟡	🟡	🟡	🟢	Low Risk
Butler 2004 [83]	🟡	🟡	🔴	🟢	🟢	🟡	🔴	High Risk
Cadmus-Bertram 2019 [84]	🟢	🟢	🔴	🟢	🟢	🟡	🟢	Low Risk
Carr 2013 [148]	🟢	🟢	🟡	🟡	🟡	🟢	🔴	High Risk
Coelho 2018 [43]	🟢	🔴	🔴	🔴	🟢	🟡	🔴	High Risk
Compernolle 2015 [85]	🟢	🟡	🔴	🟢	🔴	🟢	🔴	High Risk
Creel 2016 [86]	🟡	🟢	🔴	🟢	🔴	🟢	🔴	High Risk
Croteau 2004 [129]	🟢	🟡	🔴	🟢	🟢	🟢	🔴	High Risk
Croteau 2007 [130]	🟡	🟡	🟡	🟢	🟢	🟢	🔴	High Risk
Cruz 2016 [44]	🟢	🟢	🟡	🔴	🔴	🟢	🟢	High Risk
Dadaczynski 2017 [87]	🟢	🟡	🟡	🔴	🔴	🟢	🔴	High Risk
De Blok 2006 [88]	🟡	🟡	🟢	🟢	🔴	🟡	🔴	High Risk
De Greef 2010 [73]	🟡	🟢	🔴	🟢	🟢	🟡	🟢	Low Risk
De Greef 2011 [89]	🟢	🟢	🔴	🟢	🟢	🔴	🟡	High Risk
De Greef 2011 [74]	🟡	🟡	🔴	🟢	🟢	🟡	🔴	High Risk
Demeyer 2017 [90]	🟢	🟢	🔴	🟢	🟢	🔴	🟢	High Risk
Dishman 2009 [91]	🔴	🟡	🟡	🔴	🔴	🟡	🔴	High Risk
Dlugonski 2012 [45]	🟢	🟢	🔴	🔴	🟢	🟡	🔴	High Risk
Duru 2010 [92]	🟢	🟢	🔴	🟢	🟢	🔴	🟢	High Risk
Eakin 2014 [46]	🟢	🟢	🔴	🟢	🔴	🟢	🟢	High Risk
Edney 2020 [47]	🟢	🟢	🔴	🟢	🟢	🟡	🟢	Low Risk
Engel 2006 [93]	🟢	🟢	🔴	🔴	🟢	🟡	🟡	High Risk
Finkelstein 2016 [48]	🟢	🟢	🔴	🟢	🔴	🟢	🟢	High Risk
Fischer 2019 [132]	🟢	🟢	🔴	🟢	🔴	🟢	🟢	High Risk
Fjeldsoe 2010 [94]	🟢	🟢	🔴	🔴	🟢	🟡	🔴	High Risk
Fjeldsoe 2015 [49]	🟢	🟢	🔴	🟢	🔴	🟢	🟢	High Risk
Furber 2010 [50]	🟢	🟢	🟢	🔴	🟢	🟡	🟡	Low Risk
Gell 2015 [95]	🟡	🟡	🔴	🔴	🟢	🟡	🔴	High Risk
Gill 2019 [96]	🟡	🟡	🔴	🔴	🟢	🟢	🟢	High Risk
Glasgow 2012 [156]	🟢	🟡	🔴	🔴	🔴	🟢	🟡	High Risk
Glynn 2014 [97]	🟢	🟢	🟢	🟢	🟢	🟢	🟢	Low Risk
Golsteijn 2018 [133]	🟢	🟡	🔴	🟢	🔴	🟢	🟢	High Risk
Hardeman 2020 [98]	🟢	🟢	🔴	🟢	🟢	🟢	🟢	Low Risk
Harris 2018 [75]	🟢	🟢	🔴	🟢	🟢	🟢	🔴	High Risk
Hornikx 2015 [99]	🟡	🟡	🔴	🟢	🟢	🟢	🔴	High Risk
Hospes 2009 [100]	🟡	🟡	🔴	🟢	🟢	🟡	🔴	High Risk
Houle 2011 [101]	🟢	🟢	🔴	🟢	🔴	🟡	🔴	High Risk
Hultquist 2005 [102]	🟡	🟡	🟡	🔴	🟢	🟡	🔴	High Risk
Izawa 2012 [103]	🟡	🟡	🔴	🟢	🟢	🟡	🔴	High Risk
James 2015 [51]	🟢	🟢	🔴	🟢	🔴	🟢	🔴	High Risk
Kangasniemi 2015 [40]	🟡	🟡	🔴	🟢	🔴	🔴	🔴	High Risk
Katzmarzyk 2011 [104]	🟢	🟢	🔴	🟢	🔴	🟢	🔴	High Risk
Kawagoshi 2015 [105]	🟡	🟡	🔴	🟢	🔴	🟡	🟢	High Risk
Kendzor 2017 [134]	🟡	🟡	🔴	🟢	🟢	🔴	🟢	High Risk
Kernot 2019 [52]	🟢	🟢	🔴	🔴	🔴	🟢	🟢	High Risk
Keyserling 2008 [135]	🟢	🟢	🔴	🟢	🟢	🟢	🔴	High Risk
Kim 2018 [136]	🟡	🟡	🔴	🟢	🔴	🔴	🟢	High Risk
King 2008 [137]	🟢	🟡	🔴	🔴	🟢	🟡	🔴	High Risk
King 2013 [106]	🟢	🟡	🔴	🔴	🟢	🟢	🔴	High Risk
Koizumi 2009 [138]	🟡	🟡	🔴	🟢	🟢	🟡	🔴	High Risk
Kolt 2012 [76]	🟢	🟢	🟢	🔴	🔴	🟢	🟢	High Risk
Lane 2015 [149]	🟡	🟡	🔴	🔴	🔴	🟡	🔴	High Risk
Li 2017 [139]	🟡	🔴	🔴	🟢	🟢	🟢	🔴	High Risk

	Random sequence generation (selection bias)	Allocation concealment (selection bias)	Blinding of participants and personnel (performance bias)	Blinding of outcome assessment (detection bias)	Incomplete outcome data (attrition bias)	Selective outcome reporting	Other	Summary
Li 2020 [107]	green	green	red	green	green	green	green	Low Risk
Long 2013 [108]	yellow	yellow	red	green	red	yellow	green	High Risk
Lynch 2019 [109]	green	green	red	green	green	red	red	Low Risk
Lyons 2017 [110]	green	green	red	green	green	red	green	High Risk
Maher 2015 [53]	green	green	red	green	green	green	green	Low Risk
Mailey 2010 [150]	yellow	yellow	red	green	green	yellow	red	High Risk
Mansi 2015 [54]	yellow	green	red	green	green	green	green	Low Risk
Martin 2015 [111]	yellow	yellow	red	green	green	green	red	High Risk
Maselli 2019 [55]	green	green	red	red	green	yellow	red	High Risk
Maxwell-Smith 2019 [140]	green	green	red	green	green	green	green	Low Risk
Melville 2015 [112]	green	green	red	green	red	red	green	High Risk
Mendoza 2015 [113]	green	red	yellow	green	green	green	green	Low Risk
Merom 2007 [114]	green	yellow	red	red	green	yellow	red	High Risk
Motl 2011 [151]	yellow	green	red	red	green	yellow	green	High Risk
Müller 2016 [56]	green	green	red	red	green	red	green	High Risk
Murawski 2019 [57]	green	green	red	red	green	yellow	green	High Risk
Mutrie 2012 [58]	green	green	red	green	green	green	green	Low Risk
Nolan 2017 [59]	green	green	red	green	red	green	red	High Risk
Oliveira 2019 [60]	green	green	red	green	red	green	green	High Risk
Paul 2016 [115]	red	red	red	green	green	yellow	red	High Risk
Pekmezi 2017 [141]	yellow	yellow	red	green	green	red	red	High Risk
Pinto 2013 [61]	yellow	yellow	yellow	red	green	red	green	High Risk
Pinto 2015 [62]	yellow	green	red	green	green	yellow	green	Low Risk
Poirier 2016 [116]	green	red	red	green	red	green	yellow	High Risk
Pope 2018 [117]	green	red	red	green	red	yellow	red	High Risk
Prestwich 2009 [142]	green	yellow	red	red	red	yellow	red	High Risk
Prestwich 2010 [118]	green	green	red	red	green	yellow	red	High Risk
Reijonsaari 2012 [119]	green	green	green	red	red	green	green	High Risk
Ribeiro 2014 [63]	green	green	red	green	red	green	red	High Risk
Roos 2014 [120]	green	green	red	green	green	yellow	red	High Risk
Rowley 2019 [121]	yellow	red	red	green	red	yellow	red	High Risk
Samuels 2011 [143]	yellow	yellow	red	green	red	yellow	red	High Risk
Schwerdtfeger 2012 [154]	yellow	yellow	red	green	green	yellow	red	High Risk
Sharp 2016 [144]	green	yellow	red	red	red	yellow	red	High Risk
Simons 2018 [64]	green	green	red	green	red	green	green	High Risk
Spence 2009 [122]	yellow	yellow	red	red	green	yellow	red	High Risk
Stacey 2016 [65]	green	red	red	green	red	red	red	High Risk
Sugden 2008 [152]	green	yellow	red	green	green	yellow	red	High Risk
Suggs 2013 [66]	green	yellow	green	red	red	yellow	red	High Risk
Tabak 2014 [123]	green	green	red	green	green	green	green	Low Risk
Talbot 2003 [67]	green	yellow	red	green	green	yellow	red	High Risk
Ter Hoeve 2018 [68]	green	green	red	green	green	red	red	High Risk
Thorndike 2014 [124]	green	green	yellow	green	green	yellow	yellow	Low Risk
Thorsteinsen 2014 [153]	yellow	green	red	red	green	yellow	green	High Risk
Tudor-Locke 2004 [69]	yellow	yellow	red	green	red	yellow	red	High Risk
Unick 2012 [145]	yellow	yellow	red	green	green	yellow	red	High Risk
Vallance 2008 [70]	green	green	red	red	red	green	green	High Risk
Vallance 2016 [125]	green	green	red	green	green	yellow	red	High Risk
Van Blarigan 2019 [126]	green	red	red	green	green	green	green	High Risk
Vandelanotte 2018 [146]	green	green	green	red	red	green	green	High Risk
Van der Weegen 2015 [71]	green	green	red	green	green	green	red	High Risk
Van Hoye 2018 [72]	green	yellow	red	green	green	green	red	High Risk
Warren 2014 [127]	green	green	red	green	red	green	red	High Risk
Wijsman 2013 [147]	green	green	red	green	green	green	green	Low Risk

	Random sequence generation (selection bias)	Allocation concealment (selection bias)	Blinding of participants and personnel (performance bias)	Blinding of outcome assessment (detection bias)	Incomplete outcome data (attrition bias)	Selective outcome reporting	Other	Summary
Wyke 2019 [77]	🟢	🟢	🔴	🔴	🟢	🟢	🔴	High Risk
Yamada 2012 [128]	🟢	🟢	🔴	🟢	🟢	🟡	🟢	Low Risk
Yates 2017 [78]	🟢	🟢	🔴	🟢	🟢	🟢	🟢	Low Risk

Note: Table depicts risk of bias assessment per bias domain and an overall summary assessment. Summary assessment ranked as high if one domain other than "Blinding of participants and personnel" is ranked high.
🟢= low risk, 🟡= unclear risk, 🔴= high risk.
