# Supplementary material for: Long-term Effectiveness of mHealth Physical Activity Interventions: Systematic Review and Meta-analysis of Randomized Controlled Trials
Source: J Med Internet Res. 2021 Apr 30;23(4):e26699. doi: 10.2196/26699 (PMC8122296; doi:10.2196/26699)
Supplement: Multimedia Appendix 11 [file jmir_v23i4e26699_app11.pdf]

Multimedia Appendix 11. Grading of recommendations, assessment, development, and evaluation quality of evidence profile.

Quality Assessment						Summary of Findings				
						Number of Participants				
Number of Studies*	Risk of Bias	Inconsistency	Indirectness	Imprecision	Publication Bias	mHealth	Control	Effect Size as SMD (95% CI)	Signi-ficance	Quality
Walking (75 RCTs)	*Very Serious* 58/75 studies high risk of bias based on Cochrane criteria. Numerous studies with short intervention duration, small samples sizes and high attrition. As in most mHealth studies, blinding of participants and personnel is not possible.	*Not serious* Very high level of heterogenicity (I^2=84%) but range of results limited with 68/75 studies showing positive effects and CI [0.37-0.57]	*Not serious* Populations and interventions relevant	*Not serious* Low baseline risk (<5%), narrow confidence interval, sample size 12301)	*Undetected* Moderate sample size (mean=197, range=21-1442), funnel plot analysis does not suggest systematic publication bias.	6307	5994	0.47 [0.37-0.57]	*P*<.001	●●○○
MVPA (61 RCTs)	*Very Serious* 47/61 studies high risk of bias based on Cochrane criteria. Numerous studies with short intervention duration, small samples sizes and high attrition. As in most mHealth studies, blinding of participants and personell is not possible.	*Not serious* Moderate to high level of heterogenicity (I^2=63%) but range of results limited with 53/61 studies showing positive effects and CI [0.22-0.36]	*Not serious* Populations and interventions relevant	*Not serious* Low baseline risk (<5%), narrow confidence interval, sample size 10861)	*Undetected* Moderate sample size (mean=225, range=29-1442), funnel plot analysis does not suggest systematic publication bias.	5787	5074	0.29 [0.22-0.36]	*P*<.001	●●○○
Total PA (34 RCTs)	*Very Serious* 29/34 studies high risk of bias based on Cochrane criteria. Numerous studies with short intervention duration, small samples sizes and high attrition. As in most mHealth studies, blinding of participants and personnel is not possible.	*Not serious* Moderate to high level of heterogenicity (I^2=68%), moderate range of results [0.16-0.38] and 28/34 studies finding positive effects.	*Not serious* Populations and interventions relevant	*Not serious* Low baseline risk (<5%), narrow confidence interval, sample size 5144)	*Undetected* Moderate sample size (mean=189, range=15-1113), funnel plot analysis does not suggest systematic publication bias.	2702	2442	0.27 [0.16-0.38]	*P*<.001	●●○○
Energy Expenditure (8 RCTs)	*Very Serious* 7/8 studies high risk of bias based on Cochrane criteria. Numerous studies with short intervention duration, small samples sizes and high attrition. As in most mHealth studies, blinding of participants and personnel is not possible.	*Very serious* High level of heterogenicity (I^2=87%), large effect range CI [0.13-1.00], small sample.	*Not serious* Populations and interventions relevant	*Not serious* Low baseline risk (<5%), narrow confidence interval, sample size 834)	*Undetected* Small sample size (mean=128, range=37-463), funnel plot analysis does not suggest systematic publication bias.	481	353	0.57 [0.13-1.00]	*P*=.01	○○○○

Abbreviations: GRADE, Grading of recommendations, assessment, development, and evaluation; RCT, randomized controlled trials; CI, confidence intervals

* Number of studies that report results at end of intervention.
